# Myosin repertoire expansion coincides with eukaryotic diversification in the Mesoproterozoic era

**DOI:** 10.1186/s12862-017-1056-2

**Published:** 2017-09-04

**Authors:** Martin Kollmar, Stefanie Mühlhausen

**Affiliations:** 1 Group Systems Biology of Motor Proteins, Department of NMR-based Structural Biology, Max-Planck-Institute for Biophysical Chemistry, Göttingen, Germany; 2 Department of Biology and Biochemistry, The Milner Centre for Evolution, University of Bath, Bath, UK

**Keywords:** Eukaryotic evolution, Mesoproterozoic era, Last eukaryotic common ancestor, Horizontal gene transfer, Myosin

## Abstract

**Background:**

The last eukaryotic common ancestor already had an amazingly complex cell possessing genomic and cellular features such as spliceosomal introns, mitochondria, cilia-dependent motility, and a cytoskeleton together with several intracellular transport systems. In contrast to the microtubule-based dyneins and kinesins, the actin-filament associated myosins are considerably divergent in extant eukaryotes and a unifying picture of their evolution has not yet emerged.

**Results:**

Here, we manually assembled and annotated 7852 myosins from 929 eukaryotes providing an unprecedented dense sequence and taxonomic sampling. For classification we complemented phylogenetic analyses with gene structure comparisons resulting in 79 distinct myosin classes. The intron pattern analysis and the taxonomic distribution of the classes suggest two myosins in the last eukaryotic common ancestor, a class-1 prototype and another myosin, which is most likely the ancestor of all other myosin classes. The sparse distribution of class-2 and class-4 myosins outside their major lineages contradicts their presence in the last eukaryotic common ancestor but instead strongly suggests early eukaryote-eukaryote horizontal gene transfer.

**Conclusions:**

By correlating the evolution of myosin diversity with the history of Earth we found that myosin innovation occurred in independent major “burst” events in the major eukaryotic lineages. Most myosin inventions happened in the Mesoproterozoic era. In the late Neoproterozoic era, a process of extensive independent myosin loss began simultaneously with further eukaryotic diversification. Since the Cambrian explosion, myosin repertoire expansion is driven by lineage- and species-specific gene and genome duplications leading to subfunctionalization and fine-tuning of myosin functions.

**Electronic supplementary material:**

The online version of this article (10.1186/s12862-017-1056-2) contains supplementary material, which is available to authorized users.

## Background

The origin of the eukaryotes dates back to the Paleoproterozoic era (2500 to 1600 Ma) [1–3]. The evidence are microfossils showing complex morphology, complex cell wall ultrastructure, and multicellular forms [4]. While the taxonomic classification of these records is unclear, fossils that can be assigned to extant taxonomic groups start to appear in the late Mesoproterozoic (~1200 Ma) [5]. In contrast, molecular estimates often underestimate clade divergence times and usually date the origin of the eukaryotes to 1100–1000 Ma and the radiation of the crown groups to ~850 Ma [6, 7]. However, several recent studies have demonstrated agreement with paleontological data suggesting that previous molecular clock studies were mislead by inadequate molecular data (mostly low taxonomic and gene sampling), poor treatment of fossil calibrations, and overly simplistic treatment of the heterogeneous rates of molecular evolution [3, 8–11]. The increasing availability of multi-gene data from diverse lineages also led to stabilization of the relationships among eukaryotes culminating in four major groups: Amorphea (Opisthokonta and Amoebozoa; also termed Unikonta), Excavata, SAR (Stramenopiles, Alveolata, and Rhizaria) and Haptophyta, and plants (Glaucophyta, Rhodophyta, and Viridiplantae) [3, 12–15]. The placement of many divergent protists (e.g. Jakobida, Cryptophyta, Diplomonada) and the exact relation of the four major groups remain controversial.

The last eukaryotic common ancestor (LECA) already had an amazingly complex cell compared to prokaryotes, and possessed genomic features such as spliceosomal introns and cellular features such as mitochondria, a standard nucleus, an endo-membrane system interconnected by a complicated vesicular machinery, cilia-dependent motility, and a cytoskeleton together with several intracellular transport systems [16–18]. All these features require numerous functionally interacting proteins and coordinated biochemical activities that all must have appeared before the LECA further diverged. Intracellular movement involves three types of cytoskeletal components: actin filaments, intermediate filaments, and microtubules. Actin and tubulin have prokaryotic homologs [19, 20]. Increasing evidence suggests that lamin, the nuclear type of intermediate filaments, was also present in the LECA and might have homologs in the prokaryotic domains [21]. In contrast, ATP-driven activity by motor proteins alongside the cytoskeletal filaments is unique to eukaryotes. Myosins move along actin while the microtubule track is used by dynein and kinesin motors. The LECA contained at least nine different dyneins, of which eight are associated with axonemal motility [22], and most probably 13 types of kinesins involved in meiosis, mitosis, cilia/flagella functions, and intracellular vesicle transport [23]. Myosins are best known for their functions in muscle contraction, generation of membrane tension and transport of organelles and vesicles [24, 25]. In contrast to dyneins and kinesins, myosins are extremely divergent in extant eukaryotes with 30 to 40 defined myosin types, and a unifying picture of their evolution from a common set in the LECA to major eukaryotic branches has not yet emerged [26–29]. Here, we addressed this question by manually curating 7852 myosins from 929 species presenting first insights into the myosin repertoires of multiple lineages, which until now have not been analysed in great detail, and by providing an unprecedented dense taxonomic sampling that allows reconstruction of myosin gain and loss at high resolution. We evaluated the influence of missing data and integrated gene structure conservation in myosin classification and evolution. Based on these data and analyses, we were able to show that myosin repertoire expansion, loss of myosin functions, and myosin diversification by duplication mainly happened in successive steps during eukaryotic evolution.

## Results and Discussion

### Myosin identification and classification

**Fig. 1:**
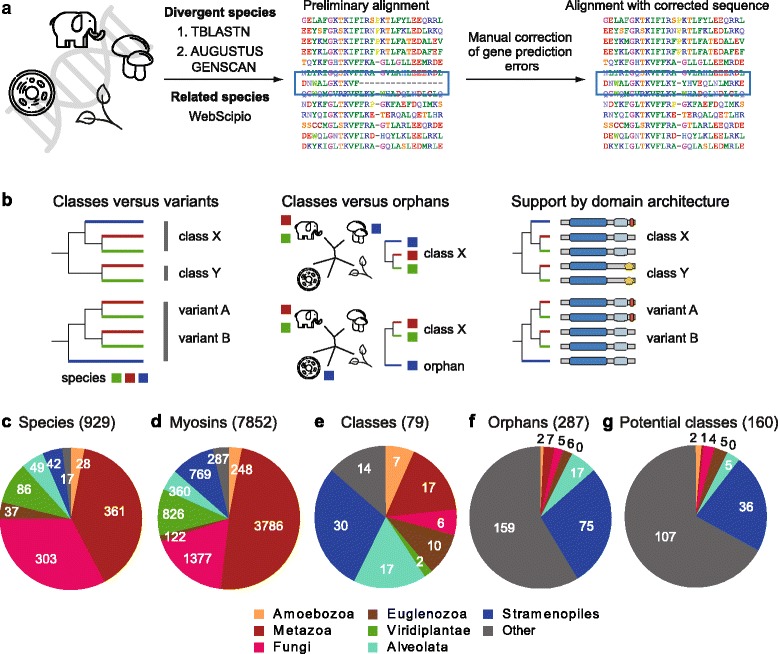
Dataset statistics. (**a**) Scheme of the myosin identification and manual gene reconstruction process. (**b**) Rationale for choosing appropriate nodes for myosin classification. (**c**) Number of species within selected major taxa. Only taxa with more than five species in subtaxa were selected. The remaining species were grouped as “others”. Although the analysed species are dominated by metazoans, fungi and plants, myosin repertoires were also identified for 173 species from other taxa. (**d**) Number of annotated myosins per taxon. Again, most myosins were derived from metazoans, fungi and plants, but there are also 1786 myosins from other species. (**e**) Number of myosin classes per taxon. Please note that some classes are shared between the selected taxa, and thus the total number of classes is lower than the sum of the classes shown in the pie chart. Although the analysis is dominated by metazoan, fungal and plant myosins, the myosin divergence is similarly complex in other taxa. (**f**) Number of “orphans” (currently unclassified myosins) per taxon. Most of the orphans belong to taxa with low taxonomic sampling, e.g. the genomes of only one or two species of the respective taxon are available. (**g**) If the 271 orphans were classified based on phylogenetic grouping and domain architecture, this would result in further 160 classes, of which most would belong to so far underrepresented taxa

The myosin protein family is known to be particularly diverse and to be characterised by multiple and independent gene losses that happened throughout eukaryotic evolution. Thus, it is not possible to choose representative species for obtaining a comprehensive picture of myosin evolution. Instead, we performed deep sequence and taxonomic sampling covering both as many major lineages and related species as possible, resulting in a final dataset of 7852 myosins from 929 species (Fig. 1, Additional file 1: Text, Additional file 1: Figures S1-S3). In order to minimize the effects of missing or wrong sequence data on phylogenetic tree computations we extensively manually corrected automatic gene predictions. Every effort has been made to not only correctly predict and reconstruct myosin motor domains but also to improve tail domain sequences to get the best representation of myosin domain architectures. However, tail domains are usually less conserved than motor domains and some myosins with unique tail architecture might still contain incorrect sequence (Additional file 1: Figures S3-S5).

**Fig. 2:**
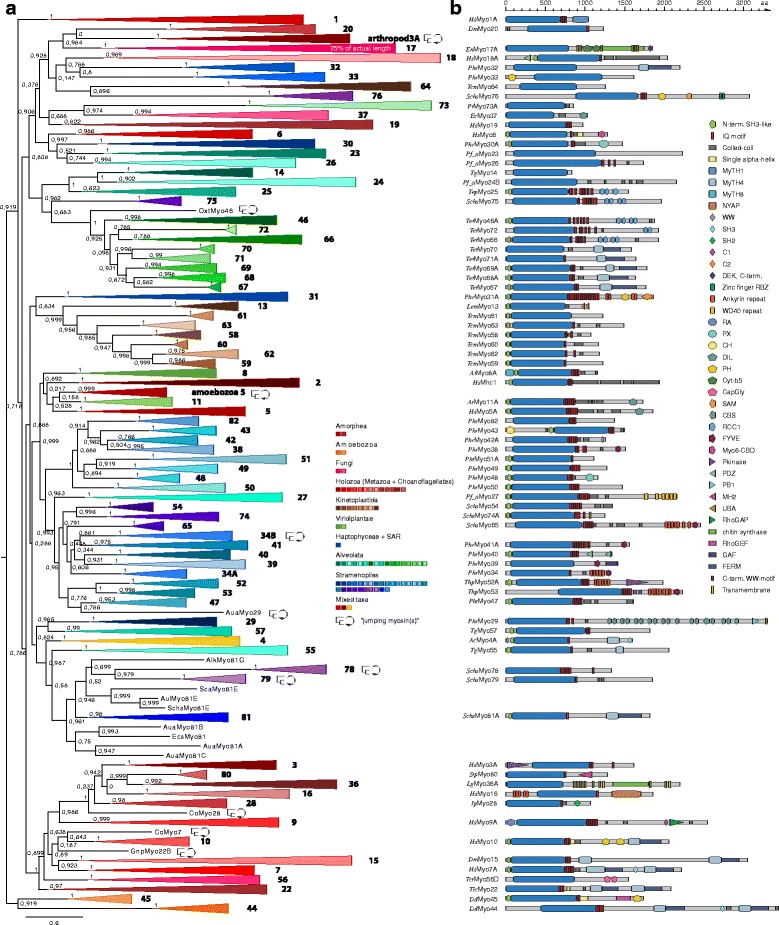
Myosin phylogeny and domain architecture. (**a**) Maximum-likelihood topology generated under the JTT + Γ model as implemented in FastTree. The tree is based on dataset6 applying a 90% sequence identity cut-off and removing orphans and some very divergent myosins resulting in 3104 myosin motor domain sequences (Additional file 1: Text). Although the entire myosin dataset is somewhat biased against metazoan and fungi species, the data used for the tree reconstruction is relatively balanced with not even two times more amorphean sequences as sequences from all other taxa. All branches containing only myosin members of a single class have been collapsed for better presentation. Some “jumping myosins”, for example the arthropod class-3A myosins (former class-21), do not group with the other members of their class in the tree of this dataset. Further trees of slightly different datasets are shown in Additional file 1: Figures S6 and S7. The scale bar represents the estimated number of amino acid substitutions per site. Myosin classes are coloured by class, not by taxa, but we used similar colours for taxon-specific classes as far as possible. (**b**) Scheme showing the domain architectures of selected members of the 79 myosin classes drawn to scale. The sequence name of a selected member of each class is given in the motor domain of the respective myosin. Regions not having assigned a defined domain do not necessarily indicate variable regions but rather missing domain definitions and might be highly conserved within the classes. Members have been chosen from well-known model species. However, they do not represent the full diversity of the domain architectures present in the respective classes. A key to all domain names and symbols but the motor domain is given on the right. The name of the representative myosin (species abbreviation + class and variant) is given in the myosin motor domain. Domain abbreviations are given in Additional file 1: Text. Species abbreviations are available in Additional file 2: Table S1

Initial myosin classification was based on motor domain phylogeny. The myosin superfamily is particularly complex, compared to other cytoskeletal protein families, necessitating stringent criteria for choosing appropriate nodes to distinguish classes from variants (Figs. 1 and 2, Additional file 1: Text). For example, setting a parent node of two classes in the phylogenetic tree as new class-defining node will turn different myosin subfamilies (classes) into subfamily variants (same class). Variants result from ancient gene duplication and there is usually a species with a single class member that diverged before the duplication event. Variants are also expected to have similar domain architectures while classes usually acquired new domains. Thus, we compared the domain architectures and species phylogenies of the leaves in the tree and combined myosins into a class until choosing parent nodes would considerably break species relations and myosin architecture similarity. Classes should have high bootstrap support and be stable independent of changes to the dataset. We observed that a few single myosins and some small groups of myosins group differently in trees from different datasets. These “jumping myosins” are very divergent members of their classes and they mutually influence their phylogenetic grouping. The “correct” placement of one subgroup seems to lead to misplacement of other groups. To obtain stable phylogenetic groupings, “missing links” coming from higher taxonomic sampling of the respective sequences/groups are needed. Here, we classified these “jumping myosins” by analysing trees of full-length myosins and by comparing gene structures (Fig. 2, Additional file 1: Figures S6 and S7). Readers interested in the full details of the classification process are recommended to read the Additional file 1: Text.

### Intron position conservation as an additional and independent criterion for myosin classification

**Fig. 3:**
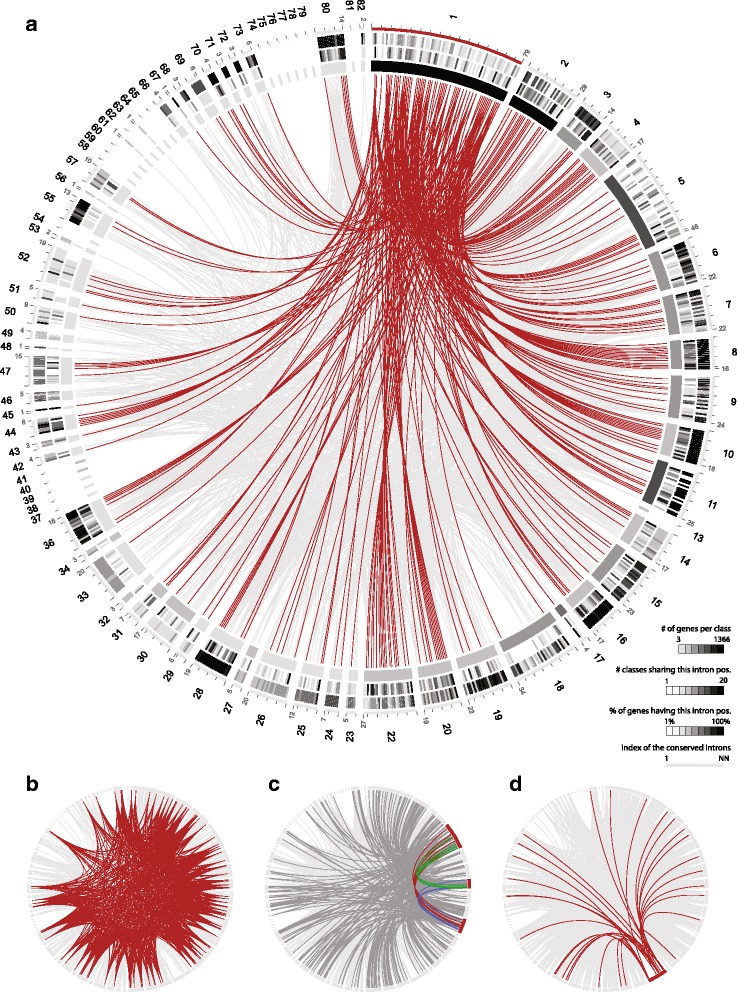
Conservation of intron positions within myosin genes. To determine and present patterns of intron position conservation across the myosin classes we plotted all myosin classes in a circular layout using Circos [58]. Orphan myosins represent up to 160 independent classes (see Fig. 1e) and are therefore omitted from this analysis. Myosin classes are represented by bands whose length equals the number of introns conserved in that particular class. Intron positions shared between classes are connected. (**a**) From outside to inside, the tracks represent (each corresponding to the respective myosin class): index of conserved intron positions (ticks every five intron positions); percentage of genes containing an intron position (one bin per intron position); number of classes sharing an intron position (one bin per intron position); number of sequences. All intron positions shared with class-1 are highlighted. (**b**) The seven intron positions shared by at least ten classes are highlighted. They most likely represent intron positions present in the ur-myosin. (**c**) The plot shows the intron positions shared within class-5, −8, and −11 myosin and with any other class (dark grey). Intron positions common to class-5 and class-8 are coloured green, those common to class-5 and class-11 are coloured red, and those common to class-8 and class-11 are coloured blue. (**d**) All intron positions shared with class-18 myosins are highlighted. All plots were generated with Circos [58]

All sequenced eukaryotes contain at least a few spliceosomal introns. Many studies comparing gene structures across ancient eukaryotic lineages suggest an intron-rich eukaryotic ancestor and describe intron loss to have happened at a substantially higher rate than intron gain [30–32]. Therefore, we assumed that gene structure conservation could provide additional protein sequence-independent information for myosin classification. Fortunately, the myosin motor domain is one of the largest known protein domains [33]. This considerably increases the chance to observe intron position conservation even in genes from species with very low intron densities. Myosin gene structures were reconstructed with WebScipio [34]. The regions coding for the motor domains were than compared with GenePainter [35], which resolves conserved intron positions at nucleotide resolution. To obtain class-specific intron position patterns we wanted to exclude that the intron patterns are dominated by genes with low intron densities (e.g. the intron-poor fungal myosin genes) or by multiple (so far) unique intron positions (e.g. from branches with low taxonomic sampling). Given the high intron loss rates in all eukaryotic lineages it is also clear that most intron positions are only present in a minority of the available sequences. Therefore, to become part of a class-specific intron position pattern an intron position needs to be present in a minimum number of genes. This number depends on the number of sequences available for a myosin class. These intron positions were termed “conserved intron positions”. The gene structure comparison resulted in 349 (421 including orphans) conserved intron positions across all myosin classes, of which 156 (47%; 221 respectively 52% including orphans) are shared between at least two myosin classes (Fig. 3 and Additional file 1: Figures S8-S10). Accordingly, 193 (200 including orphans) conserved intron positions are unique to a single class. Still, some classes contain only single exon genes (e.g. class-37 and classes-74 to −79) or not enough genes with introns to determine intron patterns (Fig. 3a). The conservation of the intron positions across myosins of the same class is in agreement with the phylogenetic tree-, species- and domain architecture-based class assignment. The gene structure comparison also does not show any common introns – apart from the generally conserved introns – between the class-5 and class-11, and the class-2 and class-18 myosins (Fig. 3c and d). The intron position conservation also supports the class assignment of the “jumping myosins” (Additional file 1: Text).

### Myosin diversity across eukaryotes

Myosins have been identified in almost all eukaryotes sequenced so far. Still, a few species do not contain myosin genes. We propose that the red algae *Porphyridium purpureum* and *Chondrus crispus* (rhodophytes), and the diplomonad *Spironucleus salmonicida* are species without myosins. Previously, we have described the absence of myosins in the unicellular red algae *Cyanidioschyzon merolae* (rhodophyte), the flagellated protozoan parasite *Giardia lamblia* (diplomonad), and the protozoan parasite *Trichomonas vaginalis* (parabasalid) [27]. For *Giardia* and *Trichomonas* the genomes of several strains are available. Thus, we can exclude that the lack of myosins is due to genome assembly gaps. The absence of myosins could be branch-specific (Diplomonadida, Parabasalia) or species-specific. To date, this cannot be conclusively revealed as only single species of these branches have been sequenced. At the very least red algae are not myosin-free in general, which is demonstrated by *Galdieria sulfuraria* and its myosin gene [27].

The myosins group into 79 classes, of which 70 currently have at least five members (Additional file 1: Figure S1C). two hundred eighty-seven sequences (termed orphans) from 69 species still remain unclassified (Fig. 1) although some of these orphans are close homologs to established classes. Examples are the lophotrochozoan orphans that have the same domain architecture as the newly defined class-80 myosins, which contain most prominently a MH2 domain (MAD homology 2; also called DWB domain: domain B in dwarfin family proteins) at the C-terminus. However, in all trees of motor domain sequences these sequences are polyphyletic and form a sister group to or group together with the class-36 myosins. Improved taxonomic sampling will help to unambiguously resolve their class membership. Other examples are three choanoflagellate orphan myosins, which are closely related to the class-3 myosins and even share the N-terminal phospho-kinase domain. Because the node of the supposed last common ancestor is not strongly supported and because they do not form a sister group to the class-3 myosins in all trees, we suggest to not yet terming these three choanoflagellate orphans class-3 myosins. Other orphans are mainly specific to species unique for their branch (e.g. *Cyanophora paradoxa*, *Guillardia theta*, *Aureococcus anophagefferens*) or specific to branches consisting of only two related species (e.g. *Ectocarpus siliculosus + Saccharina japonica*, *Emiliania huxleyi + Prymnesium parvum*, *Monosiga brevicollis + Salpingoeca rosetta*). Assigning classes to the 287 orphan myosins by merging within-species gene duplicates and cross-species homologs would result in 160 additional, distinct myosin types (Fig. 1, Additional file 1: Figure S7). Accordingly, it seems likely that the current number of myosin classes will at least triplicate as soon as more species of the respective branches become sequenced.

We based the class numbering on our previous analysis [27]. Three previously distinct, but taxonomically restricted classes (part of the “jumping myosins”, see Additional file 1: Text for more details) are now joined to broader distributed classes: the previously arthropod-specific class-21 myosins are now a subgroup of the class-3 myosins; the previously nematode-specific class-12 and the previously vertebrate-specific class-35 myosins are now subgroups of the class-15 myosins. The free class numbers were not reused to avoid confusion with previous literature. In total 47 new classes have been established (Fig. 2, Additional file 1: Figures S6 and S7, and Additional file 1: Text). There are two new classes within Metazoa: the mollusc myosins containing a chitin synthase and multiple transmembrane domains in the tail [36] are now grouped together with rotifer and annelid homologs into class-36. The new class-80 myosins have not been described so far. Members of this class are currently restricted to Ambulacraria (echinoderms and hemichordates). Closely related myosins are available in annelids and molluscs (see notes about orphan myosins above) so that the phylogenetic distribution of this class might extend in the future.

In addition to establishing new classes, we both extended the taxonomic distribution of many classes to earlier branching lineages and refined the distribution within established branches. For example, we identified class-6, class-18 and class-28 myosins in the Ichthyosporean *Capsaspora owczarzaki* thus timing the origin of these classes back to the last common holozoan ancestor. Also, we confirmed the presence of class-22 myosins in several fungal lineages [37] and found strong support for orthology with a group of amoebozoan myosins including the former *Dictyostelium* MyoI (now Myo22). Thus, we date the origin of class-22 back to the last common ancestor of the Amorphea. It is now without doubt that these amoebozoan myosins are class-22 myosins, and neither form an independent class as proposed in [29] nor belong to the class-7 myosins. While the class-19 myosins have previously not been identified in hexapods [27, 38], we were now able to identify and reconstruct class-19 myosins in Hymenoptera and Orthopteroidea. This indicates that class-19 myosins have been lost independently in most insects. The identification of a class-19 myosin in *Apis mellifera* (not detected in our previous analyses although an almost complete genome assembly was already available at that time) also shows that continuous re-analysis of species’ myosin repertoires will occasionally reveal additional, presently not detectable myosins.

### Two myosins in the last eukaryotic common ancestor

**Fig. 4:**
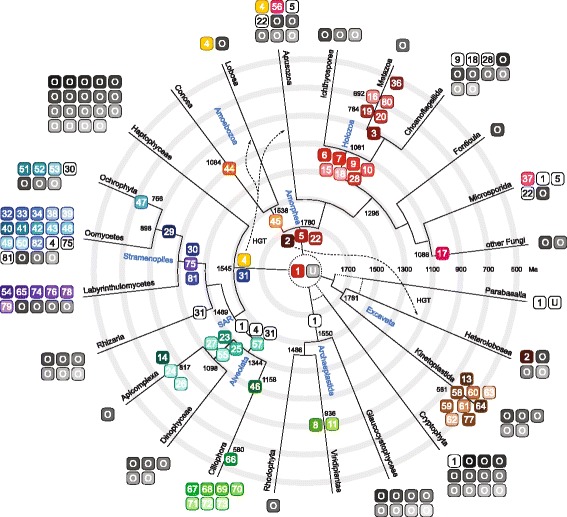
Reconstructed phylogeny of the major eukaryotic lineages, for which myosin data were available. The topology is based on a comprehensive taxon-rich study using multiple fossil records for generating a time-resolved phylogenetic tree [3] with insertions showing the most likely phylogenetic positions of Apusozoa [13], Fonticula [67], Microsporida [68], and Labyrinthulomycetes [69]. The eukaryotic root is in the center of the tree. The exact branching of the major eukaryotic supergroups is not yet completely established. Branchings still controversial are indicated by dotted lines. Numbers at nodes designate divergence time estimates and were obtained from [3] and TimeTree [43]. Numbers at branches denote divergence times of splits that are not shown because of space limitations. Putative horizontal gene transfer (HGT) events are shown by dashed arrows. Myosin class inventions were placed at nodes and are represented by filled boxes with class names while orphan myosins indicating potential further classes are shown as boxes with “O”. White boxes mark myosin loss events. Myosin classes and orphans, whose ancestry could not be assigned to nodes with known divergence times, were placed at branch ends. The supposed second myosin prototype in the LECA is indicated by an “U” in the center of the tree

To reconstruct the evolution of the myosin family, we plotted myosin class gain events onto the most commonly agreed tree of the eukaryotes [3, 12–15] (Fig. 4). Accordingly, the almost ubiquitous distribution of the class-1 myosins strongly suggests that a class-1 prototype motor was present in the LECA as proposed earlier [27]. Did the other classes evolve independently from this prototype class-1 myosins across the major domains? Class-1 myosins contain a unique and almost invariant proline insertion at the base of the lever helix of (Additional file 1: Figure S13), which makes it very unlikely that new classes evolved from class-1 myosins several times. In the latter case of multiple independent duplications, one would expect this proline to be retained (and possibly mutated) in at least one of the new classes or one of the orphans. This insertion is, however, not found in any other myosin. Given the length of the motor domain independent loss of the proline insertion seems highly unlikely. Also, a new class that evolved late from class-1 myosins would most likely have an intron position pattern more closely related to the class-1 intron pattern than to any other intron pattern. Such a closely related intron pattern is, however, also not found (Fig. 3, Additional file 1: Figure S10). These considerations suggest that the LECA must have contained another prototype myosin, from which all other classes evolved (Fig. 4). Which myosin was first, the class-1 myosin prototype or the other myosin prototype? Because all intron positions conserved in more than 8 classes are also present in class-1 myosins (Additional file 1: Figure S8), it is most likely that the ur-myosin had a gene structure with a class-1 myosin intron pattern and that the other prototype myosin appeared by duplication of the class-1 myosin prototype. In the alternative scenario, in which the class-1 prototype would have resulted from a duplication of the other myosin prototype, at least a few intron positions conserved in several myosin classes except class-1 would have been expected. Such introns are, however, not found. The class-1 specific proline insertion might have been gained in the class-1 myosin prototype after the other myosin prototype appeared, or the other prototype myosin lost the insertion before further gene duplication events happened.

### Eukaryote-eukaryote horizontal gene transfer

The only other classes with members present in more than one major lineage are class-2 and class-4. Both classes are ubiquitous in one lineage (class-2 myosins in Amorphea and class-4 myosins in Rhizaria, Haptophyceae and Stramenopiles) and are otherwise narrowly distributed in isolated, late-diverging branches (Fig. 4). This distribution can be explained by two scenarios: The first suggests appearance of class-2 and class-4 in Amorphea and SAR/Haptophyceae, respectively, followed by horizontal gene transfer (HGT, Fig. 4). In the second scenario both classes would have been present in the LECA and subsequently been lost independently in many lineages (Additional file 1: Figure S14). *Naegleria* species contain three class-2 myosins that all group with the amoebozoan class-2 myosins in the phylogenetic trees. In contrast, five of the six heterolobosean class-1 myosins group basal to all amorphean class-1 myosins. Therefore, the phylogenetic data suggest an origin of the class-2 myosins in the last common amorphean ancestor, and gain of the class-2 myosins in heteroloboseans by HGT from an early Amoebozoa. The alternative scenario of a class-2 myosin in the LECA is not in agreement with the phylogenetic grouping of the heterolobosean homologs and would require at least three independent class-2 myosin loss events, two loss events in the ancestors of the kinetoplastids and parabasalids and one in the ancestor of the Diaphoretickes (SAR + Archaeplastida) if the Diaphoretickes is considered a monophyletic taxon (Additional file 1: Figure S14). Even more loss events would have to be considered if other similarly likely early and independent branchings of the SAR and Archaeplastida were assumed [3, 12, 14]. Given the importance of class-2 myosins in cytokinesis in extant species [39] and given that non-muscle class-2 myosins are the only invariant myosins in all amorphean species it seems likely that their ancestor had a similarly complex machinery. This means that this machinery would have been lost multiple times independently of each other, if class-2 myosins were present in the LECA. Alternatively, class-2 myosins might have had a different function during early eukaryotic evolution and their role in cytokinesis had been established later independently in the ancestors of the Amorphea and the Heterolobosea.

Class-4 myosins are present in Stramenopiles, Rhizaria and Haptophyceae and, in addition, in the unrelated Lobosa (Amoebozoa) and Apusozoa taxa (only a single apusozoan species, *Thecamonas trahens*, has been sequenced so far; Fig. 4). The restricted distribution of class-4 myosins outside the SAR/Haptophyceae branch alongside with their phylogenetic grouping to rhizarian class-4 myosins and their domain architecture shared with rhizarian class-4 myosins suggests that an early lobosan or apusozoan species obtained a class-4 prototype by HGT from an ancient Rhizaria. Subsequently, the ancient Lobosa and Apusozoa shared the class-4 myosin via another HGT event. In an alternative scenario, a class-4 myosin would have been present in the LECA and subsequently been lost in multiple lineages (Additional file 1: Figure S14). This scenario, however, is not compatible with the phylogenetic grouping of the class-4 myosins, and would also include multiple tail domain changes in haptophycean and Stramenopiles class-4 myosins while the rhizarian, lobosean and aposozoan class-4 myosin domain architectures remained conserved. A phylogenomic analysis of the *Acanthamoeba castellanii* (Lobosa) and *Naegleria gruberi* genomes has identified hundreds of genes arisen through inter-kingdom HGT [40]. Although a genome-wide analysis of inter-domain HGT between eukaryotes is still missing, it seems possible that these phagotrophic protozoans also acquired a significant number of eukaryotic genes from phylogenetically unrelated species [41]. Assuming that the phagotrophic lifestyle had already been present in ancient loboseans, apusozoans and heteroloboseans this could explain HGT of class-2 and class-4 myosins.

The phylogenetic distribution of class-2 and class-4 myosins suggests that the HGT events in ancient loboseans, apusozoans and heteroloboseans already occurred more than 1 billion years ago. The only obvious example in our dataset suggesting gain of myosins by relatively recent HGT is the marine diatom *Nitzschia* (Stramenopiles: Bacillariophyta). *Nitzschia* is supposed to have incorporated thousands of algal genes [42], which could explain the presence of an algae-like class-11 myosin gene (Additional file 1: Figure S15A). More surprising was the identification of not-yet described HGT of heterolobosean-related genes, a class-1 myosin and three myosins related to orphan myosins from *Naegleria*, indicating integration of genetic information from multiple species from several domains into the *Nitzschia* genome (Additional file 1: Figure S15A).

### Could there have been more myosins in the last eukaryotic common ancestor?

Other studies suggested three [28] and six [29] myosin subtypes in the LECA: two myosin-1-like subtypes, and four ancestral myosins representing class-2, class-4, class-5, and class-6 myosins. According to our data, one of the myosin-1 subtypes (containing the SH3 domain C-terminal to the TH1 domain) and the class-2 and class-4 myosins are restricted to isolated late-diverging lineages apart from their main occurrence, indicating a late origin by duplication (from the universal class-1 myosin) and origin by HGT in case of class-2 and class-4 myosins, as described above. The proposed deep branching of class-6 myosins, indicated by the grouping of haptophyte myosins to class-6 myosins [29], is not supported by our data. Reasons why those few sequences from a single haptophyte (*E.huxleyi*), which miss the characteristic class-6 myosin motor domain loops and tail domains, should be grouped to class-6 myosins restricted to Holozoa were not given. While previous analyses always showed independent branchings for class-5 and -11 myosins, our data reveal considerable phylogenetic support for a common origin of class-5 and class-11 myosins in several of the trees (see for example the tree in Additional file 1: Figure S6). However, because an ancestral DIL domain-containing myosin is not yet supported by gene structure data and the phylogenetic grouping of class-5 and class-11 is inconsistent, we refrain from proposing a class-5 prototype in the LECA based on the current data. Proposing a common origin for the class-5 and class-11 myosins solely based on the shared tail domain architecture [28] seems arbitrary because many other domains are also shared between classes from different major taxa. Similarly, although MyTH4-FERM domains are present in many classes, their origin could be related to only two or three independent domain fusion events at the origin of the Amorphea and SAR. Assuming a MyTH4-FERM domain containing myosin in the LECA would accordingly entail multiple independent loss events in other major branches. The molecular phylogenetic data and gene structure comparisons currently do not support a myosin-rich LECA. Instead, our data suggest that myosin diversification started after the four major eukaryotic domains, Amorphea, Excavata, SAR and Haptophyta, and plants (Archaeplastida), had been established in early eukaryotic evolution.

### The timing of myosin gain and loss shows a burst of myosin innovation in the Mesoproterozoic era

The presence of at least 79 myosin classes in extant species alongside with two classes in the last common eukaryotic ancestor raises several questions. New classes could have emerged continuously over time or multiple classes could have appeared in “burst” events in-between the relatively short time from the formation of a lineage to the further split of this lineage. The invention of new classes might have happened at similar rates in the various major eukaryotic lineages, and myosin class evolution might coincide with major events in Earth history. New classes might also be related to major eukaryotic innovations. To correlate the evolution of myosin diversity with time we enhanced the tree of the eukaryotes with divergence time estimates from a comprehensive taxon-rich study using multiple fossil records for generating a time-resolved phylogenetic tree [3] (Fig. 4). Almost identical results are obtained when using the TimeTree Of Life divergence time estimates or the median/mean time estimates of all studies available from the TimeTree webpage that have included the respective branching [43] (Additional file 1: Figure S16A).

**Fig. 5:**
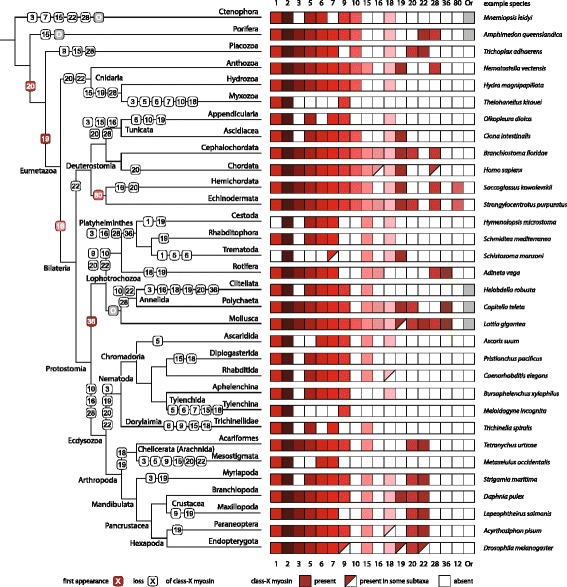
Myosin gain and loss plotted onto the most widely accepted phylogenetic tree of the metazoa. Early metazoan evolution has been adapted from [70]. The mollusc and annelid orphan myosins have identical domain architectures as class-12 myosins but have not yet been classified due to their polyphyly in phylogenetic trees. Thus, the origin of the class-12 might be considerably older than currently assigned (solid arrow). The *Mnemiopsis leidyi* orphan might represent an extremely divergent class-1 myosin but could not unambiguously be classified because part of the motor domain is missing. The *Amphimedon queenslandica* orphan myosin contains a C-terminal Rap-GAP domain, which is only present in another orphan from *Capsaspra owczarzaki*. The classes were colored using the same color scheme as in Figs. 1 and 4

Independently of whether two (class-1, unknown myosin) or three (class-1, class-2, class-4) myosins are assumed for the LECA, early eukaryotic evolution in the Mesoproterozoic era (1600–1000 Ma) is characterized by multiple myosin inventions in all major lineages (Fig. 4). The most prominent bursts happened in the ancestor of the Holozoa (Ichthyosporea, Metazoa, Choanoflagellida), the last common ancestor of the Apicomplexa and Dinophyceae (dinoflagellates), at the origin of the Stramenopiles, and, although later in time, at the origin of the Kinetoplastids. Reconstructing these early myosin innovations requires sufficiently deep taxonomic sampling, which we are the first to provide. For example, the last common holozoan ancestor acquired seven new classes (classes-6, −7, −9, −10, −15, −18, −28) resulting in a set of 11 myosin subtypes (classes-1, −2, −5, −6, −7, −9, −10, −15, −18, −22, −28; Fig. 4). The subsequent evolution towards bilateria was accompanied by only one or two new myosins at each split (Fig. 5). Notably, the origins of these five additional myosins are based on single/three species representatives of the respective taxa (Ctenophora, Porifera, Placozoa, Cnidaria). Due to the limited taxonomic sampling it seems likely that these myosin inventions will have to be assigned to earlier branchings, if not to the origin of the Holozoa, as soon as further sequenced genomes become available. In contrast to these sporadic myosin inventions in early metazoans, massive and independent myosin loss events characterize the further evolution of the metazoan myosins, and the dense taxonomic sampling now allows tracing of their evolution at high resolution (Fig. 5). The only myosins shared by all sequenced metazoans are the class-2 myosins. In contrast to HGT of class-2 and class-4 myosins between early eukaryotes, the scattered distribution of the metazoan-specific myosin classes can be explained best by multiple and independent loss events.

A burst of myosin innovation is also found in the ancestor of the Apicomplexa and Dinophyceae, which became apparent through the expanded taxonomic sampling of alveolates that provides not only strong support for our previous class assignments [27], but also demonstrates the early origin of the newly defined class-46, class-55, and class-57 myosins. Back in 2007 members of these classes were already identified [27] but not yet classified because of their limited taxonomic distribution. These ancient classes have most probably been missed in other studies because the first sequenced Apicomplexa incidentally lost many myosin genes (Additional file 1: Figure S15B). Class-26 myosins have recently been found in the dinophytes *Vitrella brassicaformis* and *Chromera velia* (data not shown but available at CyMoBase) demonstrating that improved species sampling in the future will lead to more ancient origins of many classes. Similarly, the kinetoplastids except *Trypanosoma cruzei* were long thought to contain only single class-1 and class-13 myosins. Therefore, the myosin repertoire expansion in *T.cruzei* [27] has long been regarded as species-specific. However, by identifying orthologs in the early diverging kinetoplastid *Bodo saltans* [44] and in further *Trypanosoma* relatives we can now confidently predate the myosin burst to at least the kinetoplastid ancestor.

The Stramenopiles are probably as divergent as the Holozoa/Metazoa, but gene-rich and taxonomically broad phylogenetic studies are rare and only a few dozen genomes have been sequenced so far. Accordingly, divergence time estimates for major phyla such as the Oomycetes and Ochrophyta differ by as much as 400 million years [3, 45]. Fossil records are rare, and given the Stramenopiles’ underrepresentation in taxonomically broader studies we suppose that even the oldest reported divergence times considerably underestimate their early evolution. Currently, six myosin classes are common to most Stramenopiles (classes-1, −4, −30, −31, −75, −81). More classes are specific to Labrinthulomycetes, Oomycota, and Ochrophyta, and dozens of orphan myosins have not yet been classified (Fig. 4 and Additional file 1: Figure S15A). The further evolution within these major branches resembles the metazoan myosin evolution with massive and independent myosin loss events. Therefore, we propose that analysis of further genomes will lead to an increased set of myosin classes in the last Stramenopiles common ancestor and more loss events in major subbranches, similar to the situation in the Holozoa, Alveolata, and Kinetoplastida.

**Fig. 6:**
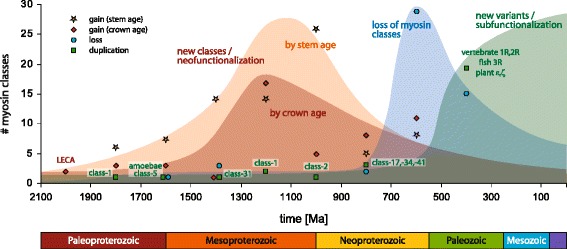
Myosin evolution in view of the history of Earth. To correlate major events in myosin evolution with geological times, we determined the density of events within time intervals of 200 million years. We distinguish three types of events: 1) Myosin gain events, which represent the appearance of new classes of myosins with novel domain architectures correlated with new cellular functions; 2) myosin loss events; 3) myosin duplications representing the generation of variants of the same myosin class, having identical or very similar domain architectures and most likely leading to subfunctionalization. For each time interval we summed up the gain and loss events denoted in Figs. 4 and 5; Additional file 1: Figure S15. Events, which could not be attributed to dated splits, were ignored. Depending on the taxonomic sampling of the study, the time intervals for subsequent speciations vary considerably across the tree of the eukaryotes (Fig. 4), and thus the timing can be very different for assigning events to stem or crown age. Therefore, we plotted the earliest possible appearance of a myosin class (stem age, data obtained from Additional file 1: Figure S16B; represented by stars) and, as a more conservative estimation, the latest possible date of myosin invention (crown age, data obtained from Fig. 4; diamonds) for comparison. The density of myosin loss events is displayed by circles. Myosin variants were identified by inspecting the phylogenetic trees and are represented by squares (vertebrate duplications can easily be referred from Additional file 1: Figure S11). Before the Paleozoic, there are only a few myosin duplications within myosin classes resulting in myosin variants, and the respective myosin classes are indicated for orientation. Myosin duplications in the Paleozoic are mainly the result of whole genome duplications (WGDs) affecting all myosin classes present in the respective ancient species. Therefore, not the affected classes but only the WGDs are indicated. All numbers represent rough estimates with respect to the accuracy and the lack of many divergence time estimates. Shaded areas were drawn around the estimated densities of events for simplified orientation

Our data showing a Mesoproterozoic origin in myosin invention and a late Proterozoic start of myosin loss events (Fig. 6) are in contrast to the recent hypothesis based on a LECA containing six myosins [29]. Although myosin classes are not entirely comparable, in the former study [29] 25 myosin gain but also 64 loss events happened up to a similar branching depth as shown in Fig. 4, assuming a root of the eukaryotes at the unikont/bikont split. Placing the root at similarly likely other branches [3, 12, 14] would not result in more gain, but even more loss events. The lower number of gain events compared to our findings is a result of the considerably lower taxonomic sampling compared to our study. However, most of the loss events resulted from proposing a myosin-rich LECA, which we suppose to be less likely than a LECA with only two myosins.

## Conclusions

As an alternative to sequence based classifications, myosins have also been grouped according to biochemical and mechanical properties which resulted in four groups from movers to strain sensors [46]. Because functional data are only available for eight sequence-based myosin classes, it remains unclear whether the remaining myosin classes fit into these four biochemical groups or whether they show completely different properties. At least the sequence data indicate that there are myosins with disrupted ATP-hydrolysis (non-functional P-loops and switches), with disrupted information transfer (non-functional relay-helix and –loop), with strongly reduced or completely missing actin-binding capacity, with disrupted converter domain, and without a lever. There are sequence insertions in almost all surface-loop regions, and these structural extensions might also considerably influence the biochemical and mechanical properties.

Although myosin research started with muscle myosin and the mechano-chemical characteristics of the muscle myosin heavy chain strongly influence our thinking about myosins, the class-2 myosins nevertheless comprise only a single class. From a sequence perspective there are also myosins without homologous regions to the functional loops of class-2 myosins who only have the residues determining the fold in common with other myosins. Here, we determined all sequences with homology to the myosin motor domain as a folding unit independent of whether newly identified myosins show similarity in the major functional loops. Of course, most sequences suggest a functional P-loop, switch-I, switch-II, relay-helix and –loop, and lever.

Reconstructing myosin evolution and classifying myosins strongly depend on gene and taxonomic sampling. To circumvent the problem of finding representative species, we identified, reconstructed and annotated 7852 myosins in 929 sequenced species. In all taxa there are species with very different numbers of myosins (ranging from species with one or a few myosins to species with dozens of myosins grouping into many classes), so that a selection of species would not resemble the myosin diversity within taxa but bias the analysis. Of course, also the current dataset only represents a transient state. Future analyses of more sequenced genomes will certainly lead to a revision in many aspects. In terms of myosin subtypes, our data suggest that the number of assigned myosin classes (currently 79) will at least triplicate. This is a very conservative estimate and myosin diversity will certainly increase further if haptophytes, rhizarians, glaucophytes, and cryptophytes show similar divergence as for example holozoans and stramenopiles. Genomes for several early-branching eukaryotes such as the jakobids and malawimonads are presently not available at all, and their sequencing and analysis is expected to result in the identification of further myosin diversity. Increased sampling density might occasionally lead to class fusions in the future, similar to the former nematode-specific class-12 myosins, which we showed here to belong to class-15 myosins. In terms of myosin evolution, we could show that many classes are more ancient than previously thought. However, the taxonomic sampling in many lineages (e.g. Euglenozoa, Ciliophora and Stramenopiles) is not yet sufficient to reveal the origin of many myosin subtypes. In concordance with the early burst of myosin innovation found in branches with dense taxonomic sampling, we predict a Mesoproterozoic origin of most of the classes found in lineages with currently low taxonomic sampling as well as an early origin for most of the currently unclassified myosins.

The overall high taxonomic sampling of our present study also uncovered extensive myosin loss. Myosin loss happened in more recent eras than previously thought, often included loss of multiple myosins at once, and happened independently in all major eukaryotic lineages (Figs. 4 to 6 and Additional file 1: Figure S15). We anticipate that further increase of taxonomic sampling density in already broadly covered taxa, such as the Metazoa, Apicomplexa, and Oomycota, will not reveal many new classes, if at all, but will uncover higher diversity in myosin inventories due to different selections of myosins that were lost.

Beyond multiplying myosin diversity our data reveal further remarkable and surprising results. The phylogenetic analyses and gene structure comparisons strongly suggest only two myosins in the LECA, a class-1 myosin and a second myosin, which is not present in extant species anymore. The sparse distribution of class-2 and class-4 myosins outside their major lineages contradicts their presence in the last eukaryotic common ancestor but instead strongly suggests early eukaryote-eukaryote HGT. In addition, the respective species involved are phagotrophic amoebae, which have a high potential for HGT. Early eukaryote-eukaryote HGT is extremely difficult to detect [41] and these myosin HGT events would be, to our knowledge, the first examples of eukaryote-eukaryote HGT in early diverging eukaryotes. Mapping the origin of myosin classes onto a time-resolved phylogenetic tree showed bursts of myosin innovation in all major eukaryotic branches largely coinciding with the Mesoproterozoic era (Fig. 6). Although the timing of many myosin inventions is still unclear, it seems that myosin innovation fades out during the Neoproterozoic. The burst of myosin classes in all major taxa during the Mesoproterozoic era is followed by massive and independent myosin losses after lineage splits during the late Neoproterozoic era. Myosin losses still happened in the Phanerozoic eon towards extant species but less dramatically than during the Neoproterozoic era. Myosin evolution within the Phanerozoic eon is mainly characterized by massive gene duplications. Myosin gene duplications are the result of whole genome duplications (WGDs) such as the 1R and 2R WGDs in the ancestor of the vertebrates (Additional file 1: Figure S11) and the many WGDs that happened in plant evolution [47]. In addition, myosins were duplicated in late-diverging branches (e.g. duplication of the class-3 and class-7 myosins in the ancestor of arthropods and insects, respectively) and in (according to current sequence data) single species (e.g. duplication of muscle myosin heavy chain genes in the leech *Helobdella robusta* and the owl limpet *Lottia gigantea* [48]; see the myosin inventory table at CyMoBase for more examples). Together, these gene duplications indicate extensive subfunctionalization (Fig. 6).

The observed myosin burst events suggest that they coincide with major cellular innovations. However, only amorphean, apicomplexan and plant myosins have been studied in detail so far and functional data are missing for most of the myosin classes. Although several domains are shared between myosins of the major lineages, the domain combinations are unique, with few exceptions. The diversity of tail domain architectures suggests that myosins were adapted to different cellular functions in the major eukaryotic domains. Our data provide the foundation for many future biochemical, structural, and cellular studies of myosins and acto-myosin-based cytoskeletal dynamics.

## Methods

### Identification and annotation of the myosin heavy chain genes

Building on the myosin repertoire of our previous study [27] we followed two strategies to identify and assemble further myosin sequences. A) We reconstructed homologs in species belonging to taxa for which complete myosin repertoires have already been determined with WebScipio [34] using the myosins of the closest related species as query sequences and adjusting the search parameters to allow the correct or almost correct reconstruction of protein homologs down to about 80% sequence identity (−-min_score = 0.1 --min_identity = 0.3 --max_mismatch = 0 [allowing any number of mismatches] --multiple_results --exhaust_align_size = 15,000 --exhaust_gap_size = 100 --max_move_exon = 10 --gap_to_close = 10). In addition, we performed TBLASTN searches in the respective genomes with myosins from different classes. With this strategy, we minimized the risk to miss more divergent myosin homologs, which might have been derived by species-specific inventions, or myosins which are not present in the query species’ myosin repertoire due to species-specific gene loss events or due to species-specific assembly gaps. B) We obtained myosin heavy chain genes in new or divergent taxa, which could not be reconstructed with the WebScipio approach, essentially as described [27]. Shortly, we identified myosin genes in TBLASTN searches starting with the protein sequence of the *Dictyostelium discoideum* class-2 myosin motor domain (Additional file 1: Figure S1). We then submitted the respective genomic regions covering the search hits to AUGUSTUS [49] to obtain preliminary gene predictions. However, feature sets are only available for a few species and therefore almost all predictions contained incorrect sequences and/or missed exons. Sometimes, AUGUSTUS completely failed to identify even a single of the suspected coding regions of the putative myosin gene. In those cases the homologous regions determined by TBLASTN were taken as starting point for manual myosin gene reconstruction. Wrong and missing sequence regions became apparent when comparing the predicted protein sequences to other, already corrected myosin sequences in the multiple sequence alignment. Missing exons were manually added by inspecting the three-reading-frame translations of the respective genomic DNA regions, and sequences wrongly predicted as exonic were identified and manually removed (Additional file 1: Figure S2). This approach was necessary especially for multi-exon genes, but even single-exon genes were often mispredicted with wrongly assigned introns and starting-methionines. Divergent regions within the motor domains and within the tail domains were reconstructed by simultaneously manually comparing the three-reading-frame translations of the respective genomic DNA regions of homologous myosins of related species (Additional file 1: Figure S2). Translations conserved in all respective species were considered as exonic. Potential exon borders needed to be conserved with respect to reading frame and splice-site pattern (see Additional file 1: Text for more details). Comparison with available Pfam [33] protein domain profiles also helped in correctly reconstructing the myosin tail regions in cases where only few comparative myosin sequence data were available. This domain profile-based approach helped to resolve the gene prediction errors for example in *Monosiga brevicollis* and *Salpingoeca rosetta* myosins (the only available choanoflagellates), and in *Ectocarpus siliculosus* and *Saccharina japonica* myosins (the only available Phaeophyceae). In cases where comparative genomic data were not available (e.g. in the case of the haptophyte *Emiliania huxleyi*) or where sequence regions were too divergent (e.g. the tail regions not showing homology to any annotated domains), we searched in the available EST and transcriptome shotgun assembly (TSA) data. This helped, for example, to resolve many mispredicted regions in *Emiliania huxleyi* myosins (by comparison with the TSA data of *Prymnesium parvum*) and in arthropod and echinoderm class-3 and class-15 myosin tails.

In addition to identifying new sequences, we have updated previously incomplete sequences and filled sequence gaps by analysing newer genome assemblies wherever possible. For consistency, we updated all sequences derived from cDNA sequencing to match the genomic DNA-encoded sequences. Examples are the mouse, rat and thale cress sequences from the 1990s.

Many myosins contain alternative splice variants. Here, we included the 5′ exons from each cluster of mutually exclusively spliced exons, and retained the differentially included exons as far as they could be determined (see Additional file 1: Text for more details).

### Incomplete genes and pseudogenes

In concordance with our previous analysis [27] we termed incomplete sequences “Partials” and “Fragments”, if up to 5% or more than 5%, respectively, of the supposed full-length sequences were missing due to genome assembly gaps or incomplete EST/TSA data. The “Partials” and “Fragments” status was assigned to the myosin motor domains and the tail regions separately (see Additional file 1: Figure S3 for examples). This allows assessing the completeness and reliability of the data. For example, a complete tail region of a TSA-derived myosin indicates that the gene reconstructions of orthologous myosins from related species are most probably of high reliability, independently of whether the corresponding motor domain of the TSA-derived myosin is also complete or fragmented. For the phylogenetic reconstructions we excluded all partial and fragmented motor domains, because the phylogeny might be affected by incomplete sequences. Still, these “Partials” and “Fragments” are very important for correcting gene predictions and for defining myosin repertoires. Five sequences from our dataset were termed pseudogenes, because they contain more in-frame stop codons, frame-shifts and missing sequences than sequencing and genome assembly problems could account for. An example is the human Mhc20 myosin (*MYH16* gene) which corresponds to the superfast myosin in other mammals.

### Generating the multiple sequence alignment

Comparison of the available >60 myosin motor domain crystal structures showed that all conserved secondary structure elements are present in all structures. Thus, it is highly likely that these elements are present in all myosins and that the core parts of these elements are conserved in length (i.e. there cannot be any insertion or deletion within the middle of α-helices and β-strands because these would disrupt all spatial interactions of the subsequent C-terminal parts of these secondary structural elements). To our knowledge, none of the available alignment software is able to keep such secondary structural elements as uninterrupted blocks. Therefore, we decided to build on our previous structure-guided multiple sequence alignment [27]. This alignment was built on the crystal structure of *Dictyostelium* Myo1E [50] containing the following secondary structure (according to DSSP, numbers denote the length of the element in amino acids, α = alpha-helix, β = beta-strand, no-key = loop): 3(α)–5–13(α)–3–4(β)–4–4(β)–10–7(α)–4–3(α)–4–15(α)–3–6(β)–6(P-loop)–15(α)–6–20(α)–2(β)–11(switch-I)–8(β)–5–9(β)–3–4(α)–8–2(β)–7(α)–4–7(α)–5–3(α)–1–5(α)–10–14(α)–4–17(α)–4–4(β)–8–4(β)–2–11(α)–3–9(α)–16–2(β)–2–31(α)–8–6(β)–8(switch-II)–2(β)–1–32(α)–13–5(α)–7–10(α)–5–11(α)–6–2(3)–13–6(β)–2–6(β)–3–6(α)–4–8(α)–4–6(α)–15–16(α)–3–8(β)–12–11(α)–1–9(α)–3–3(β)–1–11(α)–11–10(α)–5–3(α)–4(β)–4–3(β)–3–8(α). Most loops are conserved in length across almost all myosins and the loop sequences were kept as block with the preceding secondary structural element. In several cases such as the P-loop and switch-I and -II regions, subsequent secondary structural elements were kept together with the interrupting loop as combined uninterrupted blocks. Such an alignment with larger uninterrupted regions is a prerequisite for identifying and correcting gene prediction errors, and – once established – is easy to both maintain and extend.

Newly predicted myosin heavy chain sequences were added individually to the existing structure-guided multiple sequence alignment [27]. The work-flow for adding a new sequence was as follows: we first determined the closest myosin homolog with a BLASTP search against all myosins included in CyMoBase (updated on a regular basis), then pre-aligned the new sequence to the closest homolog using ClustalW [51], and finally added the pre-aligned sequence to the multiple sequence alignment of all myosins. Subsequently, we verified the correct alignment of every new sequence by manually adjusting the pre-aligned sequence if necessary. We validated the sequences as described above by manually removing wrongly predicted sequence regions and filling gaps (Additional file 1: Figure S2). Sequences derived from low-coverage genomes often contain many gaps. Automatic alignment software is not aware of these gaps and thus often generates global alignments instead of local alignments with gap regions. We on the other hand maintained the integrity of exons preceding and following these gaps, and added alignment gaps accordingly. In case newly added myosins contained species-specific extended loops we adjusted the entire full myosin alignment accordingly. Similarly, divergent sequence regions were re-aligned as soon as further related sequence data became available (Additional file 1: Text). The final myosin motor domain alignment used in this study contains 3490 alignment positions and is available at Figshare.

To exclude that our manually generated alignment leads to biased phylogenetic trees, we generated a MAFFT alignment (13,907 alignment positions), which results in similar trees (Additional file 1: Text). However, the MAFFT alignment contains only a few alignment blocks longer than 5 aa precluding its use in correcting gene predictions and in supporting gene structure comparisons.

### Domain and sequence motif prediction

Protein domains were predicted using HMMER3 [52] against the Pfam v.28.0 database [33] accepting all domains with E > 0.001. Transmembrane helices were predicted with TMHMM v.2 [53], single α-helices (SAH) with Waggawagga [54], coiled-coil regions with coils [55], and sequence motifs with PROSITE [56]. Domain ranges were corrected and domains manually added to the myosin schemes shown in Fig. 2, if additional domains were predicted in orthologous myosins and if these domains were supported by homology in the multiple sequence alignment. Domains, or parts of domains, could have been missed because of the applied E-value cut-off, or because they had not been identified at all. The latter happens if sequences are too divergent with respect to the Pfam domain profiles, which are generated based on seed alignments of small sets of supposed representative domain family members. Here, especially the N-terminal SH3-like, the MyTH4, and the FERM domains are often not recognized using Pfam profiles although sequence homology is striking when manually inspecting the myosin sequence alignment. This has already been shown in detail before [27, 47].

### Preparing datasets for phylogenetic and intron conservation analyses

The dataset used in the phylogenetic and intron conservation analyses consists of 7748 myosins from 919 species. The genomes and TSAs of several taxonomically important species became available at a later date so that these were only included in the qualitative analysis. These species include the amoebae *Balamuthia mandrillans*, the stramenopiles *Nitzschia sp. ChengR-2003*, *Thalassiosira rotula*, *Hemiaulus sinensis*, *Leptocylindrus danicus*, *Cylindrotheca closterium*, and the rhizarian *Plasmodiophora brassicae*. Their myosin repertoires were annotated by BLAST searches against all other myosins, and used for better dating myosin class invention and loss events. The total dataset thus contains 7852 myosins from 929 species.

The dataset of 7748 myosin sequences was too large for most Bayesian and Maximum-Likelihood phylogenetic analyses. However, reducing the dataset, other than by removing identical sequences, introduces specific bias: A) Decreasing the number of sequences reduces sequence variability, i.e. a single sequence does not represent the variation within ten closely related sequences. B) Reducing the length of the alignment by removing “poorly aligned positions” decreases class separation. Our approach of aligning the sequences in uninterrupted blocks of secondary structural elements allows residues with different chemical properties at same alignment positions and thus takes compensatory mutations in spatially close regions into account. These positions are, however, regarded as “poorly aligned” by respective software.

We generated a basic dataset from all myosin motor domains by a) removing “Fragments”, “Partials”, and pseudogenes, b) removing the divergent ascomycote class-17B and the class-77 myosins, c) removing the extremely divergent *Schs*MyoE orphan myosin, and d) applying a 90% redundancy cut-off using CD-Hit [57] to reduce bias from Amorphean myosins which form the largest group in the dataset. Building on this basic dataset, we generated further datasets by a) removing all orphans, b) adding all class-7, −10, −15, −22 myosins, c) adding all class-3 myosins, d) adding all class-3, −16, −28, −36, −80 myosins, e) adding the choanoflagellate orphans (which group closely to the class-3 myosins), f) independent and combined removal of the Panagrolaimoidea class-15 myosins, and the jumping *Oid*Myo9, *Thkl*Myo9, and *Bx*Myo7 myosins, and g) combinations thereof (Additional file 1: Text).

### Detection of conserved introns

Intron conservation in the myosin motor domains was analyzed with GenePainter v.2 [35], applied separately for each myosin class. GenePainter maps gene structures onto protein sequences from multiple sequence alignments. Introns separating codons at different nucleotides are treated as different intron positions. The divergent loop 1 and loop 2 regions were excluded from the analysis because of the ambiguous protein sequence alignment in these regions. To account for the different sequence sampling in each class, we applied different cut-offs for intron conservation depending on the number of sequences per class: i) For less than 50 sequences, introns were required to occur in at least 10% of the sequences, ii) for up to 100 sequences in 7.5%, iii) for up to 400 sequences in 5%, and iv) for more than 400 sequences, conservation in at least 1% of all sequences was required. To identify intron positions shared between classes, the class-wise exon-intron patterns were compared, and common intron positions were visualized using Circos [58].

### Computing and visualising phylogenetic trees

Phylogenetic trees were generated using the Maximum-Likelihood method as implemented in FastTree v. 2.1.10 [59] with estimated proportion of invariable sites and bootstrapping (1000 replicates). ProtTest v.3.2 failed to run on the basic dataset and the other, larger datasets. We tested alignments with 50, 60, and 70% sequence identity cut-offs generated with CD-Hit. These tests suggested the LG + Γ model to be the most appropriate. However, this finding cannot be extrapolated to the larger datasets. Therefore, we generated phylogenetic trees for every dataset using the JTT + Γ, the WAG + Γ, and the LG + Γ amino acid substitution models as implemented in FastTree.

To exclude that the approximations to handle large datasets as implemented in FastTree considerably influence myosin tree reconstruction we computed a phylogenetic tree with RAxML-HPC-Hybrid v. 8.2.8 [60] using the high-performance parallel computing implementation at CIPRES [61]. The datasets as described above are far too large for obtaining trees within months of computation time [62]. To generate a small enough but still representative dataset, we reduced the redundancy within the basic dataset to 50% with CD-Hit. It should be noted, that the proportion of orphan myosins within this dataset is 18.5%, compared to 5.8% within the basic dataset (90% redundancy cut-off). The resulting alignment consists of 788 myosin sequences and 3053 alignment positions. The amino acid substitution model was set to LG + Γ + I and the option to halt bootstrapping automatically was turned on (RAxML stopped after 252 bootstraps replicates). Although we tried various Bayesian tree reconstruction approaches, these failed to converge on the basic dataset within months of computation time.

The intron conservation tree was generated using MrBayes v. 3.2.1 [63] with binary data and datatype “restriction”. Two independent runs with 10,000,000 generations, four chains, and a random starting tree were performed. Trees were sampled every 1.000th generation and the first 25% of the trees were discarded as “burn-in” before generating a consensus tree. Phylogenetic trees were visualized with FigTree v. 1.3.1 [64].

## Additional files

Additional file 1: This document contains extensive additional information on the myosin classification strategy, comparison of alternative software for alignment and phylogenetic tree generation, myosin naming conventions, description of the datasets used for tree generation, and all additional figures. (PDF 30652 kb)

Additional file 2: List of species, species abbreviations, and species taxonomy, for which myosins were identified and assembled. (XLS 298 kb)
