# Supplementary material for: Myosin repertoire expansion coincides with eukaryotic diversification in the Mesoproterozoic era
Source: BMC Evol Biol. 2017 Sep 4;17:211. doi: 10.1186/s12862-017-1056-2 (PMC5583752; doi:10.1186/s12862-017-1056-2)
Supplement: Supplementary file 1 — This document contains extensive additional information on the myosin classification strategy, comparison of alternative software for alignment and phylogenetic tree generation, myosin naming conventions, description of the datasets used for tree generation, and all additional figures. (PDF 30652 kb) [file 12862_2017_1056_MOESM1_ESM.pdf]

Supplementary Information

Myosin repertoire expansion coincides with eukaryotic diversification in the Mesoproterozoic era

Martin Kollmar[1§] and Stefanie Mühlhausen[1,2]

[1] Group Systems Biology of Motor Proteins, Department of NMR-based Structural Biology, Max-Planck-Institute for Biophysical Chemistry, Göttingen, Germany

[2] Department of Biology and Biochemistry, The Milner Centre for Evolution, University of Bath, Bath, United Kingdom

[§]Corresponding author

Email addresses:

MK: mako@nmr.mpibpc.mpg.de

SM: stmu@nmr.mpibpc.mpg.de

Contents

1. Supplementary Text 3

1.1. Myosin classification strategies 3

1.2. Combining molecular phylogeny, species phylogeny, domain architecture analyses and gene structure comparisons for myosin classification 4

1.3. The advantages of high taxonomic sampling compared to choosing representative species 6

1.4. Myosin classification by molecular phylogeny and species phylogeny 7

1.5. Divergent class members mutually influence their grouping causing “jumping” from within classes to outside of classes 9

1.6. Domain architectures might differ between myosins of the same class 10

1.7. Myosins tail domains 11

1.8. Intron position conservation supports the phylogeny-based classification of the “jumping myosins” 13

1.9. Evolution of intron position patterns 14

1.10. Myosin subfamily variants from gene and genome duplications 15

1.11. Correcting ambiguous exon borders and transcription start sites 16

1.12. Comparing our manual sequence alignment to a MAFFT-generated alignment 17

1.13. Comparing the FastTree generated trees to a RAxML generated tree 18

1.14. Naming myosins 19

1.15. Alternatively spliced myosins 20

1.16. Data availability 20

1.17. Description of the myosin datasets used for phylogenetic tree reconstructions 20

1.18. Domain abbreviations 22

2. References 23

3. Supplementary Figures 25

1. Supplementary Text

1.1. Myosin classification strategies

In most previous studies, myosins were classified based on phylogenetic trees computed from their motor domain sequences [1–5]. The rationale for assigning classes has been twofold: i) the parent node with the highest support by bootstrapping replicates (distance-based methods and Maximum-Likelihood approaches) or posterior probability estimates (Bayesian analyses) and ii) the congruence of myosin domain architectures. Problems arise when defining the parent node. In some early studies, parent nodes combined myosins with identical or almost identical domain architectures [1, 2]. We previously used the nodes with the deepest taxonomic sampling that do not combine two completely unrelated taxa [3]. Others classified myosins only by domain architectures [4], or only by myosin phylogeny [5]. This resulted in many classes containing myosins with different domain architectures and from unrelated taxa. Most of the 17 classes defined 16 years ago [2] were re-used in all later studies despite the differences in class assignment definitions. On the other hand, no commonly agreed on classification of new myosins emerged. Instead, classifications are now overlapping, diverging and contradicting.

From the beginning of myosin classification until now it has always been tried to bring domain architecture-based classification in congruence with phylogeny-based classification. Back then, a new class has been assigned to every new myosin with new domain architecture. Myosins with identical domain architectures were treated as subtypes of the same class. This rationale has been fostered by a study proposing the coevolution of the motor, the neck and the tail domains [6]. However, using the rather macroscopic domain features for classification might be as similarly misleading as reconstructing species phylogenies only by a few morphological characters. Myosins are multi-domain proteins and tail domains could have independently been acquired and/or rearranged. Domain definitions themselves impose another drawback on this approach. Domains are usually defined by comparison with domain database profiles, which are, however, inherently biased by the available sequence data. Therefore, divergent domain homologs are not identified, leaving the respective sequences "domain-free". The domain architecture problem has already been evident in the last commonly agreed classification [2] and has led to several inconsistencies: myosins with identical domain architectures have been split into different classes (e.g. the class-12 and class-15 myosins, and the class-5 and class-11 myosins), and myosins with

different architectures were grouped into the same class (e.g. class-1). Furthermore, single sequences with unique domain architectures were treated inconsistently: metazoan myosins were favourably designated a class number while unique myosins from other eukaryotic lineages were not classified further and termed "orphans" [2].

Together with the highly metazoan/fungi/plant-biased genome sequencing this generated a metazoan-centric view on myosin diversity. This trend is still present in the most recent study reporting eight to eighteen classes in Metazoa (and unicellular Holozoa) and up to six classes in fungi, but only two or three classes in alveolates, rhizarians, cryptophytes and kinetoplastids [5]. We, on the other hand, have already shown years ago that myosin diversity in other eukaryotic lineages might be as extensive as in metazoans although we did not designate a new class to each myosin with a new domain architecture [3]. Thus, in the present study we had to resolve problems in sequence and taxonomic sampling, and we had to develop guidelines for incorporating molecular phylogeny, species phylogeny, and domain architecture data into a consistent classification scheme. In addition, we introduce gene structure conservation as additional and independent information in resolving ambiguous class assignments.

1.2. Combining molecular phylogeny, species phylogeny, domain architecture analyses and gene structure comparisons for myosin classification

In protein family trees, the topology of each subfamily should coincide with the species phylogeny allowing distinction between subfamilies and subfamily variants derived by taxon-specific gene duplications. Subfamily variants typically have a single subfamily homolog in a common ancestor of the respective species, similar domain architectures, and they originated by duplication followed by subfunctionalization. Proteins of distinct subfamilies usually acquired new functions and accordingly most likely have different domain architectures. As long as phylogenetic analyses are consistent (e.g. all subfamilies present and in agreement with taxonomic relationships) as for example in coronin [7], dynactin [8], dynein [9], and WASP family proteins [10], a representative selection of species from all taxa might be sufficient. However, the previous research on myosin phylogeny has shown that the myosins represent a particularly complex protein family characterized by dozens of different domain architectures and the absence of “expected” classes in many branches. In particular, the

absence of myosins complicates molecular phylogenetic reconstructions and the interpretation of the evolution of myosin repertoires. The best solution to overcome these problems is using deep sequence and taxonomic sampling.

Previous myosin analyses have shown some puzzling results contradicting the congruence of molecular phylogeny, species phylogeny and domain architecture. For example, the class-12 and class-15 myosins have identical C-terminal tail domain architectures, but they never grouped together in phylogenetic trees whatever advanced reconstruction approach had been applied. Most puzzlingly, the class-12 myosins were always restricted to nematodes, the only major metazoan lineage missing class-15 myosins. In other cases, myosins were joined into a single class although they did not share a single tail domain and although additional evidence from the species phylogeny was missing [5]. With high sequence and taxonomic sampling we intended to overcome many of these problems by identifying "missing links" to improve phylogenetic groupings and by higher species sampling to better distinguish subfamilies and subfamily variants. In addition, every effort has been made to correctly predict and reconstruct myosin tail domain sequences to get the best representation of myosin domain architectures. In addition to these established criteria, we reconstructed and compared gene structures to use intron position conservation as additional and independent distinguishing feature for myosin classification.

Even if myosins could be unambiguously classified, high taxonomic sampling is necessary to plot the history of myosin evolution from the ancient origin of each class to extant species' myosin repertoires. There are examples of species whose myosin repertoires suggested rich myosin diversity for the respective branch, and examples which suggest only a small set of myosins within the respective taxon. An example for a rich myosin diversity, at first hand, were the Cnidarians: the first sequenced cnidarian, *Nematostella vectensis* [11], contains an extensive myosin repertoire with 17 myosins in 12 classes. However, the very recently sequenced myxosporean *Thelohanellus kitauei* [12] contains only 4 myosins in 3 classes. On the other hand, there is no reason to believe that branches with currently limited myosin diversity do not contain species with larger myosin repertoires. To get the best possible overview on myosin diversity in currently sequenced species, we therefore analysed as many available genomes from as many taxa as possible to obtain a taxonomically balanced view on myosin evolution both at the scale of the major eukaryotic kingdoms and at high resolution within late-branching taxa.

In the next sections we will report on our efforts to generate sequence and taxon-rich data, before we discuss the results of the myosin classification based on sequence and species phylogenies. Next, we describe the correlation of tree-based classification and domain architecture analysis. We end the myosin classification sections with a description of the gene structure comparisons and the consequences for myosin classification.

1.3. The advantages of high taxonomic sampling compared to choosing representative species

Using comparative genomics strategies we generated a dataset of 7852 myosins from 929 eukaryotes, with 7339 sequences derived from 723 whole genome sequencing (WGS) projects (Fig. 1, Figs. S1-S3). Of these myosins, 6431 could be reconstructed in full-length. 6989 myosins could be reconstructed with complete motor domains. Eukaryotic genome sequencing is highly biased by economical, ecological and biotechnological interests, as well as by taxon-specific initiatives [13, 14]. This has resulted in the under-representation of many evolutionary important lineages, such as Apusozoa, Placozoa, and Cryptophyta. In addition, myosin inventories are also very different between closely related species, as we have already shown for several metazoan taxa [3, 15, 16]. From the available sequenced metazoans only molluscs encode the full set of myosins found in metazoans (classes-1, -2, -3, -5, -6, -7, -9, -10, -15, -16, -18, -19, -20, -22, -28, -36, -80). All other metazoans have lost myosin classes during their evolution. Thus, myosin diversity within metazoans cannot be represented by a few model organisms such as human, *Drosophila*, and *C.elegans*. Instead, deep taxonomic sampling of all lineages is necessary to reveal a comprehensive picture. Even taxa suspected to have invariant myosin repertoires such as the fungi have considerable divergent myosin inventories. For this reason, we refrained from analysing only single representative genomes of major taxa. Instead, we determined the myosin repertoires of dozens of closely related species, given enough genomes were available.

In addition to species-specific gain and loss events, incomplete genome assemblies with assembly gaps and errors prevent the selection of "typical species" for the reconstruction of taxon-specific myosin inventories. Amongst those incomplete genomes are, for example, the ones of the chicken (*Gallus gallus*) and the roundworm nematode *Brugia malayi* that contain only tail fragments of the class-15B myosin and the class-1D myosin, respectively. In addition, the second class-1 myosin Myo1A, which is conserved in nematodes and also

present in *Brugia pahangi*, is completely missing in the available *B. malayi* genome assemblies. Without comparing myosin inventories between closely related species, it cannot be assessed whether all myosins are present in a certain genome assembly and whether all myosins have been reconstructed without gaps.

A broad taxonomic sampling is not only important for comparing myosin inventories but also for reconstructing reliable motor domain phylogenies for myosin classification (see below). Missing and fragmented motor domains strongly influence phylogenetic groupings and support for branches. This prompted us to investigate as many genomes as possible and to subsequently exclude fragmented sequences from the phylogenetic analyses. The large fraction of short myosin fragments in others' data [5] together with considerably lower sequence and taxonomic sampling is most probably the main reason for many differences in phylogenetic groupings.

1.4. Myosin classification by molecular phylogeny and species phylogeny

As outlined in the classification strategy section, choosing another parent node in the phylogenetic tree will turn different myosin subfamilies (classes) into subfamily variants (same class). Thus, defining criteria for choosing appropriate nodes for class assignments is most important in addition to generating robust and unbiased phylogenetic trees. By generating phylogenetic trees of the myosin motor domain we observed that a few single myosins and some small groups of myosins group differently in trees from different datasets. We did not want to remove/add sequences until a supposedly "best" tree is obtained. Instead, we identified these myosins that seem to "jump" around and classified them using a different approach (see below).

For myosin classification, we defined that a group of myosins must be present as monophyletic group in all trees generated, and that this group must be supported by bootstrap support of at least 95% in most of the trees in order to be termed a 'class' (Fig. 2; Figs. S6 and S7). In fact, most of the classes are supported by 100% bootstrap support. In addition, we required and verified that the topology of the myosins within each class closely resembles the phylogeny of the respective species. A good indication for having chosen appropriate nodes for classification is that the classes remain monophyletic while the topology of the classes with respect to each other changes between the different trees. To exclude that we mis-assigned subfamily variants into classes, we searched for inner-more nodes with similar high

bootstrap support (>95%). At such nodes we would expect to find myosins from species that split before those species whose myosins are in the child nodes. However, this was not found but could be caused by missing species in the dataset. In these cases, we validated that the domain architectures are similar for all myosins within a class but are very different to the myosins of the sister class. This rationale is very obvious, for example, for separating the metazoan class-36, which contain multiple transmembrane and a chitin synthase domain, and class-82 myosins, which contain a C-terminal MH2 domain (Fig. 2), and also applies to most of the other classes. Still, there are several groups of classes with shared tail domains that consistently group together and have high bootstrap support values at the parent nodes. For instance, most amorphean MyTH4 domain-containing myosins strongly group together (classes-7, -10, -15 and -22; Fig. 2 and Fig. S6), and there are clusters of ciliate- and kinetoplastid-specific classes.

It had been proposed that the major eukaryotic lineages independently developed multiple unique myosins with divergent domain architectures [3]. As a result, multiple classes from each lineage are expected to cluster in the phylogenetic trees. Independent of the assignment of the classes, this is exactly what we find in all phylogenetic trees: within the cluster of amorphean myosins not a single myosin from other eukaryotic kingdoms is found, and similarly there are major nodes restricted to SAR, Stramenopiles, ciliates and excavates (Fig. 2; Figs. S6 and S7). Similar taxon-specific groupings are also apparent in others' analyses independent of their taxonomic bias. However, in these analyses the myosins from under-represented lineages (e.g. Stramenopiles, Alveolata, Rhizaria) have been regarded as class variants instead of distinct classes although they often have very different domain architectures [1, 5]. Following this rationale one could also group, supported by partially shared domain architectures, class-7, -10, -15, and -22 myosins into a single class. Similar to these taxon-specific clusters, the myosins from species of underrepresented lineages such as haptophytes, Rhizaria, cryptophytes and glaucocystophytes each group together. From these sparse data it is not clear whether the corresponding myosins are distinct subfamilies (as in the other taxon-specific clusters of classes) or subfamily variants, and therefore these myosins were not classified but termed "orphans". In conclusion, increased taxonomic sampling strikingly strengthened the robustness of the phylogenetic reconstructions and the separation of classes. Nevertheless, classifying myosins representing very divergent class members remains challenging.

1.5. Divergent class members mutually influence their grouping causing "jumping" from within classes to outside of classes

We have shown in detail in a large-scale analysis of the tubulin protein family that the branching of specific divergent tubulins and the grouping of tubulin subfamilies depends on dataset size and phylogenetic reconstruction method [17]. We find a similar dependency in our myosin analysis. While about 99% of the myosins group consistently in all trees, the following sequences/subgroups either group with their class or separately: *Bx*Myo7 (nematode), *Co*Myo7 (Ichthyosporea), *Oid*Myo9 (urochordate), *Thki*Myo9 (cnidarian), *Gnp*Myo22B (Monoblepharidomycota), *Co*Myo28, arthropod Myo3A (formerly "Myo21", see below), amoebae class-5 myosins, choanoflagellate Myo15, nematode class-15 myosins (formerly "Myo12"), vertebrate Myo15B myosins (formerly "Myo35"), platyhelminths Myo19, *Aua*Myo29 (Pelagophyceae), Myo34B, and Labrinthulomycete class-78 and class-79 myosins. From now on we refer to these myosins as "jumping myosins".

Where these sequences group depends on sequence number, alignment size, and sequence selection used in the tree reconstructions. For instance, reducing the number of class-15 myosins in the dataset causes the nematode class-15 myosins and the vertebrate Myo15B to group as separate classes. When including all available class-15 myosins, the vertebrate Myo15B myosins group sister to the vertebrate Myo15A myosins while the nematode Myo15 myosins still group separately. In this case the vertebrate class-15 myosins grouping would be in accordance with the whole genome duplications (WGDs) that happened at the origin of the vertebrates (called 2R). When excluding the four Panagrolaimoidea (a phylum within the Nematoda) class-15 myosins, which are the most divergent nematode Myo15 myosins, the remaining nematode Myo15 group together with *Bx*Myo7, the platyhelminths Myo15, and the amoebae Myo44 as a sister group to all other class-15 myosins (Figs. S6 and S7). Subsequent removal of *Bx*Myo7 causes the amoebae Myo44 to group sister to the Myo45, and all class-15 myosins to group together.

The choanoflagellate orphan myosins usually group close to the class-3, -28, and -36 myosins. Removing these myosins from the dataset leads to misplacement of the arthropod Myo3A myosins (former class-21 myosins) as separate group next to the class-17 and class-20 myosins (Fig. 2). On the other hand, removing the choanoflagellate orphan myosins leads to grouping of *Bx*Myo7 with the other nematode class-7 myosins, the nematode and platyhelminths class-15 myosins as sister to all other bilaterian Myo15, and the amoaebae

Myo44 as separate class next to the amoebae Myo45 (Fig. 2). These examples demonstrate that the “jumping myosins" are very divergent members of their classes and that they mutually influence their phylogenetic grouping. The “correct” placement of one subgroup seems to lead to misplacement of other groups. To obtain stable phylogenetic groupings, "missing links" coming from higher taxonomic sampling of the respective sequences/groups are needed.

We classified these "jumping myosins" based on their grouping in the majority of the reconstructed phylogenetic trees of the motor domain sequences, their grouping in the phylogenetic tree of the full-length sequences, their domain architecture, and their intron position conservation (see below). In contrast to the “jumping myosins”, the orphan myosins never group together with myosins of assigned classes. Orphans other than the choanoflagellate class-3 myosin-like proteins (see below), don’t share domain architectures with any class.

The “jumping myosin” problem has not been observed in previous analyses because these sequences were either always misplaced or were simply not included in the studies. Examples for misplacing are the nematode Myo15, which group as the separate former class-12 [2, 3], and the *Drosophila* Myo3A, which group to class-16 [5] or together with other insect Myo3A as the separate former class-21 [3]. Examples for excluding “jumping myosins” from the study are the nematode Myo15 [4, 5] and the vertebrate Myo15B [1, 4, 5]. The other “jumping myosins” listed above do not belong to model organisms and were not present in previous analyses.

1.6. Domain architectures might differ between myosins of the same class

Using the TBLASTN sequence similarity search approach we noticed that the myosin tail regions usually have lower sequence similarity than the motor domain regions. We conclude that the similarity difference between motor and tail domain sequences is an inherent characteristic of almost all myosin classes (Fig. S4). Notable exceptions are the fungal class-17 myosins that have highly conserved chitin synthase domains. While the majority of the myosins of each class have similar domain architectures, there are myosins in each class that have considerably divergent tail domains. For example, there are class-2 myosins with very short coiled-coil regions that are highly unlikely to be able to assemble into filaments, and there are class-8 and class-11 myosins without MyTH8 and DIL domains, respectively

[18]. The variations in tail lengths and domain architectures might involve up to 2000 residues (e.g. the class-15 and class-29 myosins). Thus, by identifying more class members, it becomes increasingly likely to observe a divergent domain organisation. This not only causes considerable difficulties for the gene reconstruction process requiring every divergent tail region to be evaluated and proven by data from further genome and/or transcriptome data, but also limits the use of domain architecture data in myosin classification.

Here, we used domain architecture comparisons only to support sequence-based classifications. First, we verified that all myosins within a class have similar domain architectures. If myosins have divergent domain architectures, these should be consistent with the respective species phylogeny. For example, given the long divergence times of Stramenopiles, Kinetoplastids and Amorpheans, it is acceptable that the kinetoplastid class-1 myosins and some Stramenopiles Myo1 have domain additions to the consensus architecture of motor domain, IQ-motif and MyTH1 domain (Fig. S5). Similarly, the extension of yeast class-1 myosin tails by C-terminal VCA domains, compared to metazoan Myo1, is the result of a taxon-specific domain gain event. The variation in class-15 domain architectures can be explained by branch-specific domain gain and loss events. Most class-15 myosins, including the choanoflagellate, Ichtyosporea and cnidarian homologs, have SH3-like N-terminal domains and a C-terminal tail characterised by two copies of tandem MyTH4-FERM domains interrupted by an SH3 domain. Deviating from this domain architecture, hexapods (but not crustaceans) lost the SH3 domain, and annelids, molluscs, vertebrates, and some but not all nematodes lack the N-terminal SH3-like domain. Vertebrates have instead very long N-terminal extensions of low complexity. Artificially joined classes are expected to have bipartite distributions of domain architectures, but this is not found. However, there are some classes with identical domain architectures such as the class-7 and class-15 myosins, and some ciliate myosin classes. These are separate classes according to the molecular sequence data but would be joined into single classes based on their domain architectures.

1.7. Myosins tail domains

In total, the classified myosins comprise 42 domains with known profiles of which 20 are shared by at least two classes (Fig. 2). Another four domains are shared with orphan myosins. Next to many of the domains present in classified myosins, the orphans contain an additional eleven domains. Many of the domains reported in other analyses [4, 5] are not present in our

data most likely because of our efforts in correcting wrongly predicted exons in the tail regions by which we resolved artificial gene fusions. The N-terminal SH3-like domain and the IQ motif C-terminal to the motor domain are present in most classes and all eukaryotic lineages, confirming their origin in the last common eukaryotic ancestor [3]. The domains present in N-terminal extensions, such as the PH, PDZ, RA, WW, Pkinase, CH domains and the ankyrin repeats, are also present in the C-terminal tail regions of other myosins. Also, many domains exist in tandem duplications and in multiple combinations with other domains. All this indicates that these class-specific domain architectures appeared by independent domain fusion events, rather than by divergent evolution of ancestral myosins containing one, or a specific combination, of the respective domains.

As examples for multiple independent domain acquisitions we analysed the class-1 myosins and the MyTH4 domain-containing myosins in more detail (Fig. S5). Class-1 myosins usually contain, from N- to C-terminus, the motor domain, one or more IQ motifs for binding calmodulin(s), and a TH1 (tail homology 1) domain. The TH1 domain is build of a central beta sheet adopting a PH (pleckstrin-homology) domain-like fold with alpha-helical and beta sheet extensions at its N- and C-termini, respectively [19]. The PH-like core domain is not identified as PH domain by domain profile searches indicating extreme divergence or the convergent evolution of a similar fold. There have been many lineage-specific domain gain events such as the insertion of a WW-domain into the N-terminus of the TH1 domain in euglenozoan class-1 myosins, the independent gain of FYVE, VHP, WW, and CA domains in euglenozoan, stramenopiles, and fungal class-1 myosins, respectively, the fusion with 700 aa-long N-terminal tails containing two WW domains in Heterolobosea class-1 myosins, the insertion of the post-IQ region in three subtypes of the holozoan class-1 myosins, multiple tandem duplications of the TH1 domain in Heterolobosea class-1 myosins, and the independent insertion of domains into the loop-1 sequence in Ichtyosporea, Diptera, and Amoeba class-1 myosins (Fig. S5). An example for complex rearrangements is the loss of the entire C-terminal tail (including the IQ motif) in a class-1 myosin variant in the last amoebozoan common ancestor, followed by the acquisition of a short, 30 aa tail region including a C-terminal CaaX box sequence [20]. The limited taxonomic distribution of the TH1 domain in combination with other domains in addition to the myosin domain suggests that the TH1 domain evolved from a tail sequence fused to the last common ancestral class-1 myosin and that the myosin part was subsequently and independently lost in several lineages, e.g. plants, ciliates, and Parabasalia (Fig. S12A).

It had been proposed that the earliest eukaryote contained a myosin fused to a MyTH4-FERM cassette [4] and the "invariable coexistence" of the MyTH4 and FERM domains has been repeatedly stated ever since [21, 22]. However, the class-4 and class-55 myosins, as well as many orphans, unambiguously contain MyTH4 domains without accompanying FERM domains (Fig. 2). Both FERM and MyTH4 domains also occur independently of each other in non-myosin proteins: the FERM domain is, for example, also present in the founding members of the domain, the protein 4.1, ezrin, radixin, and moesin proteins, and the MyTH4 domain occurs, for example, also in the "rho GTPase-activating protein 39" proteins (Fig. S12B). The latter is characterized by two N-terminal WW domains and a RhoGAP domain C-terminal to the MyTH4 domain, and present in Amorphea and Parabasalia. Presuming a common origin of all myosins with a MyTH4-FERM cassette, one would expect the respective myosin classes to phylogenetically group together and to share - at least partially - a common gene structure. However, both assumptions are not supported by the available data. Similarly, the available data do not support the independent acquisition of the other thirteen domains shared by several myosin classes. The data do support, however, the common origin of class-1 myosins. The taxonomically broad distribution of myosin-independent MyTH4 and FERM domain containing proteins (Fig. S12B) instead supports independent fusion of ancestral myosin domains with these ubiquitous domains in many lineages.

1.8. Intron position conservation supports the phylogeny-based classification of the "jumping myosins"

The intron position conservation supports the class assignment of the "jumping myosins". For instance, the gene structures of the vertebrate Myo15A and Myo15B (formerly "Myo35") motor domains are identical, most of the nematode Myo15 (formerly "Myo12") intron positions are shared with the vertebrate Myo15 genes but not with genes of other classes, the arthropod Myo3A (formerly "Myo21") intron positions match the class-3 intron pattern, and *Bx*Myo7 and the platyhelminths Myo19 homologs have identical intron positions as other members of these classes. Only seven of the eleven amoebozoan Myo5A myosin genes have intron positions within their motor domain regions. One of these intron positions is specific for class-5 myosins and not present in class-11 myosins. The *Dictyostelium discoideum* Myo5A, for example, does not have introns within the motor domain. These examples

strongly support our earlier conclusions that single species' myosins are not representative and that considerable species sampling is needed for comprehensive classification. Similar to using class-specific intron patterns to support phylogeny-based classifications, identical or coinciding class-specific intron patterns might also reveal the artificial split of myosins into multiple classes that instead should be grouped into a single class. The comparison of the intron patterns showed that all classes have unique, non-coinciding patterns. The intron patterns provide additional support for separating the Ciliophora myosins into eight distinct classes. These classes have completely divergent intron patterns, although, in part, similar domain architectures.

1.9. Evolution of intron position patterns

A phylogenetic tree reconstructed from the class-specific intron patterns showed that the class-1 intron pattern is basal to all other patterns and that class-3, -16, -28, -36, and -80 myosins (class-28 cluster) and class-14 and -24 myosins (class-14 cluster) have strongly related patterns (Fig. S10). There is weak support for clusters of the class-7, -9 and -15, the class-32 and -39, and the class-57 and -73 intron patterns (Fig. S10). With the exception of these classes, the intron position patterns of all other classes are equally related to each other. This suggests a very ancient origin of all classes, at least as ancient as the origin of the class-14 and -28 clusters. Although intron loss and gain rates may vary between lineages, there is no indication that homologs preferably lose different introns after duplication. Many intron positions are deeply conserved within classes, in some cases with the last common ancestor of the corresponding taxa dating back more than 800 Ma.

Seven intron positions are conserved in at least ten classes and were most likely present in the ur-myosin gene (Fig. 3B). The class-1 myosins have 79 conserved intron positions, of which 52 are shared with 57 of the other classes (79% of the classes containing at least a single conserved intron position; Fig. 3A). This suggests that the ur-myosin must have had a class-1-like myosin gene structure. In addition, the ur-myosin gene was most probably extremely intron-rich. The gene structure comparison also provides hints for dating the emergence of new classes. For example, the split between the plant class-8 and class-11 myosins might have happened shortly after split of the Rhodophyta (~1500 Ma) or later before the viridiplantae started to split (~1000 Ma), or sometime in-between. The class-8 and class-11 myosins share only four of at least 16 possible intron positions, and these positions

belong to the ones shared by most classes (Fig. 3C). If the ancient class-8 and class-11 myosins emerged shortly before the split of the viridiplantae, their intron position patterns would likely be still very similar after the split into chlorophytes and streptophytes. However, it seems highly unlikely that the 206 class-8 myosins and the 621 class-11 myosins independently of each other lost exactly those introns by chance, which the myosins of the respective other class retained. More likely, the early evolved ancient class-8 and class-11 myosins mutually lost the class-specific introns of the respective other class so that the class-specific intron position patterns were already well established before the split of the viridiplantae. These considerations rather suggest an early origin of the class-8 and class-11 myosins.

Similarly, the class-5 myosins share more intron positions with Amorphean-specific classes than with classes from other taxa. This suggests that the class-5 myosins originated from an ancestral Amorphean myosin (Fig. 3C). The class-18 gene structures do not share any specific homology with the class-2 gene structures (Fig. 3D). This finding is in contrast to the results of other researchers, who found molecular phylogenetic support for a common ancestry of class-2 and class-18 myosins [1, 5]. In summary, the gene structure data available today strongly supports the monophyly of most of the assigned classes. However, most intron position patterns do not specifically group with respect to major eukaryotic taxa indicating that more data are needed to unambiguously reveal the evolution of the myosin classes with respect to the early evolution of the eukaryotes. This is not surprising given the little information contained in gene structures compared to amino acid sequences. But the gene structures provided useful information to distinguish subfamilies from subfamily variants and to help resolving ambiguous molecular phylogenetic results such as the “jumping myosins”.

1.10. Myosin subfamily variants from gene and genome duplications

Only recently, extensive taxonomic and sequence sampling revealed the coincidence of plant myosin repertoire expansion with whole-genome duplication events allowing us to attribute the emergence of new myosin variants to specific speciation events [18]. To simplify further research and conclusions about functional homology we (re-)named all plant myosins so that orthologous and paralogous relationships become obvious. Here, we used the same approach and named (or renamed) all myosins such that genes with orthologous relationship can be distinguished from gene duplicates (paralogs). The best documented metazoan genome

duplications are the so-called 2R whole-genome duplications at the origin of the vertebrates [23], and further whole-genome duplications at the origin of the fish [24] and salmonids [25]. Whole-genome duplication events are usually followed by extensive gene loss, which we also found to be true for vertebrate myosins. Tetrapod myosins occur at most as two-copy genes. In contrast, most tetrapod myosins have duplicates in the analyzed fish (Fig. S11), indicating that extensive myosin gene loss happened after the 2R event but before the divergence of the fish lineage. Metazoan class-1 myosins separate into four distinct subgroups as observed earlier [5, 26, 27] that we named "A" to "D". Class-2 myosins distinguish into the non-muscle and the muscle myosins. Animals follow two major ways to generate multiple muscle myosin isoforms: i) mutually exclusive splicing, which is the common form in many invertebrate species [15], and ii) multiple muscle myosin genes as in nematodes and chordates. While the mammalian skeletal muscle myosin genes are arranged in a cluster of tandemly arrayed gene duplicates [28], the corresponding fish myosins are spread over multiple chromosomes [29]. In addition, the fish skeletal muscle myosins duplicated independently of each other in the various fish lineages so that orthologous relationships between fish skeletal muscle myosin genes cannot be inferred for the current dataset (Fig. S11).

Whole-genome duplications outside animals and plants have, to our knowledge, only been reported for yeast [30], *Hortaea werneckii* [31], *Rhizopus oryzae* [32], and *Paramecium* species [33]. Other known extensive duplicated regions in protozoans have been attributed to genomic segment duplications [34]. These events might explain several of the observed species- and lineage-specific myosin duplications. For instance, class-5 myosin duplication happened in the last amoebozoan common ancestor and the myosin variants in the extant amoebae have been named accordingly. However, simply comparing myosin variants is generally not enough to draw reliable conclusions on the myosin variants' functions. For example, a subtype A gene of any given species might be closer related to the subtype B gene of another species than to its subtype A gene.

1.11. Correcting ambiguous exon borders and transcription start sites

Exon borders can be misinterpreted if several splice site positions are possible. To resolve those cases, we reconstructed gene structures of all myosins derived from whole-genome sequencing projects using Scipio v.1.5 [35] and compared the gene structures class-wise with GenePainter v.2 [36]. The main splice sites are those conserved between all related species.

Myosins with differing splice sites were re-analysed and corrected accordingly. These corrections mainly affected splice donor sites. Gene prediction software searched for GT---AG splice sites, while we could validate for many introns by gene structure comparison that GC---AG splice sites are the main (and most likely correct) splice sites. Similarly, the starting methionine can often not unambiguously be deduced from single sequences. Especially one-exon genes might contain pseudo start-codons in upstream sequences, and so-called full-length cDNAs and TSA-derived sequences might not be complete but miss upstream sequences. In multi-exon genes, the first coding exon is usually not correctly identified by gene prediction software if it is a short exon or separated from the following exon by thousands of base pairs. For example, the first coding exon of a subgroup of fungal and metazoan class-1 myosins consists of just the starting methionine, and the first exons of vertebrate class-5, class-7 and class-10 myosins comprise only seven to nine residues and are separated from the second exons by up to 80,000 base pairs. These exons are not recognized by gene prediction software. Therefore, we manually determined the starting methionines and first exons by comparing gene structures and sequences from closely related species.

1.12. Comparing our manual sequence alignment to a MAFFT-generated alignment

To exclude that our structure-based alignment is biased in any way, we generated an alignment of the final, manually corrected sequence data using MAFFT v. 7.299b (2016/Jun/29) [37] for comparison. This alignment consists of the following blocks counted for class-1 myosin motor domains (block-length in amino acids, number of blocks in brackets): 1(357), 2(65), 3(33), 4(9), 5(7), 6(1), 7(1), 8(1). Thus, more than half of the motor domain sequence consists of alignment blocks of only one amino acid, and only 10 blocks consist of uninterrupted sequence regions of five or more amino acids. In contrast, the myosin motor domain structure contains 15 secondary structural elements with lengths of at least ten amino acids including the 32 amino acid long uninterrupted relay-helix, and 36 elements with lengths of five of more amino acids. Phylogenetic analysis (see main methods section for more details) of the MAFFT-alignment showed that only 53 myosins are completely mis-aligned. This demonstrates that MAFFT is able to align the myosin motor domains globally, but that the alignment is strongly disturbed locally. The MAFFT alignment of the motor domain (>13,900 alignment positions) is therefore 3 times as large as our structure-guided

alignment as result of extensively added alignment gaps. All secondary structural elements are highly fragmented into multiple short elements. Given that insertions at the respective positions are highly unlikely, those many gap positions are misleading and distract from seeing the overall conservation of the myosin structures. The size of the alignment does not allow to manually inspecting all sequence regions, but it seems that each of the interruptions is caused by a minority of the sequences (<20%, corresponds to <1,500 myosin sequences), and that the sequences causing the interruptions vary from structural element to element. Thus, it is not possible to just exclude a few or some hundred myosin sequences to get an alignment with considerably longer uninterrupted blocks. The phylogenetic tree based on this MAFFT-alignment showed the same topology of the myosin classes as the trees generated from our structure-guided alignment, including the mis-placing of the previously defined "jumping"-myosins. This demonstrates that local misalignment of part of the sequence does not considerably influence the phylogenetic grouping, and that our manually generated structure-guided alignment is at least as robust as the MAFFT-alignment. However, it would be impossible to identify and correct gene prediction errors based on such a highly fragmented MAFFT-alignment or to inspect the alignment to identify the conservation of a certain amino acid within a certain secondary structural element.

1.13. Comparing the FastTree generated trees to a RAxML generated tree

The FastTree generated trees were identical except for the placing of the jumping myosins and the topology of the myosin classes. FastTree uses some approximations for inferring phylogentic trees in order to handle very large alignments in a reasonable amount of time. To exclude software-dependent classifications, we generated a RAxML tree, which is, however, based on a considerably smaller dataset (redundancy within the basic dataset reduced to 50% with CD-Hit resulting in 788 myosin sequences). It should be noted, that the proportion of orphan myosins within this dataset is 18.5%, compared to 5.8% within the basic dataset (90% redundancy cut-off). The RAxML phylogenetic tree showed almost the same class-topology as the FastTree generated trees including placing the "jumping myosins" within their classes. This indicates that the tree topology is independent of both the tree reconstruction algorithm and redundancy within the alignment. However, the bootstrap support for most branches including most class-defining branches is below 50% in the RAxML generated tree. This is no surprise given the high diversity within the dataset (about 20% of the data are unclassified

unique orphan myosins). Apart from the low bootstrap support, the RAxML generated tree is not suitable for independent and unbiased myosin classification as the underlying data had to be greatly reduced. Many well-known myosin classes such as class-10 and class-13 myosins are represented by single representatives only. Still, although being less robust due to smaller dataset size, RAxML results in the same tree topology as FastTree. Similarity of RAxML and FastTree trees has been shown earlier for other datasets [38].

1.14. Naming myosins

Our naming scheme is, apart from the 17 classes defined 16 years ago [2], independent of other groups' naming schemes. This is because we are the first using a taxonomic sampling deep enough to differentiate distinct myosin types and subtypes. This allowed us to separate distinct myosin types into different classes while joining different subtypes into single classes. Instead of trying to merge classifications based on different sampling approaches, we extended our classification first presented in [3]. We also feel that our dataset is more complete than others', as we verified the myosin repertoires at the genomic DNA level instead of solely relying on gene prediction datasets, and thus ensured not to miss myosins. Our previously suggested nomenclature is general and extendible [3]. However, we could not resolve paralogous relationships within many lineages at that time, as the taxonomic sampling was not deep enough. For example, only recently we observed by extensively sampling plant genomes that myosins follow most known whole-genome duplication events in plant evolution [18]. We resolved orthologous and paralogous relationships of plant myosins unambiguously and renamed all plant myosins accordingly. As a result, these relationships become obvious from the naming and functional homology can more easily be concluded. Similarly, we named and renamed all other myosins so that orthologous and paralogous relationships become obvious.

The classification and naming of myosins with status "Fragment", "Partial", and pseudogene was unambiguous if orthologs of closely related species were available. In case of species, which are single representatives of their branch, orthologous myosins were often not available and the "Fragments" and "Partials" termed orphans.

1.15. Alternatively spliced myosins

Many myosin genes contain alternatively spliced exons. The most prominent cases are the metazoan muscle myosin heavy chain genes encoding mutually exclusively spliced exons and differentially included exons [15, 39]. Alternative splicing has also been reported for several other mammalian [40–43] and Drosophila [35] unconventional myosins, although systematic analyses are still missing. Most of these alternative splicing events affect the N-terminal or C-terminal tail regions. In plants, alternative splicing most likely only affects untranslated regions [18]. In the present analysis, we included the 5' exons from each cluster of mutually exclusively spliced exons, and retained the differentially included exons as far as they could be determined.

1.16. Data availability

Sequences, domain and motif predictions, and gene structure reconstructions are available at CyMoBase (http://www.cymobase.org, [44]). CyMoBase allows searching the data for specific myosin sequences, entire classes, individual species or taxa, as single selectors or in combinations. In addition, CyMoBase provides a BLASTP server allowing searching sequences by sequence homology. The results view also lists references to genome sequencing centres and citations of genome sequence analyses for every matching species. Gene structure visualizations are provided for each sequence, including a reference to the genome assembly used for reconstruction (see Fig. S3 for an example). Each gene structure is linked to WebScipio for in-depth inspection at the nucleotide level.

1.17. Description of the myosin datasets used for phylogenetic tree reconstructions

The phylogenetic trees corresponding to the following datasets are available at Figshare (https://doi.org/10.6084/m9.figshare.4565155.v1). File naming convention:

_jtt usage of the 'Jones-Taylor-Thornton' model
_wag usage of the 'Whelan and Goldman' model
_lg usage of the 'Le and Gascuel' model
_gb application of gblocks with parameters for less stringent selection of blocks

The datasets are based on the following data:

dataset1

7748 myosin motor domains were obtained from CyMoBase. Then, all fragmented motor domains and pseudogenes were removed resulting in 7313 myosin motor domains (=> dataset1). This dataset shows that all partial motor domains are well classified even if some sequence regions are missing.

dataset2

Dataset1 was taken and all partial motor domains removed: 6899 myosin motor domains.

dataset3

Dataset2 was taken and CD-hit with a similarity cutoff of 90% applied to reduce redundancy of highly similar sequences: 3376 myosin motor domains.

dataset4

Dataset3 was taken, and the very divergent ascomycote class-17B myosins, the class-77 myosins, and the SchsMyo_E orphan myosin were removed. The three *Amoebidium parasiticum* (Ichthyosporea) Myo10 myosins (although fragments and partials) were added to support better grouping of other basal Myo10 sequences: 3309 myosin motor domains.

dataset5

Dataset4 was taken, the *Amoebidium* Myo10, and the divergent OidMyo9 (*Oikopleura dioica*), ThkiMyo9 (*Thelohanellus kitauei*), BxMyo7 (*Bursaphelenchus xylophilus*), and the Panagroleimoidea RhabMyo15 (*Rhabditophanes* sp. KR3021), PrtMyo15 (*Parastrongyloides trichosuri*), StrMyo15 (*Strongyloides ratti*), StpaMyo15 (*Strongyloides papillosus*) and StsMyo15 (*Strongyloides stercoralis*) were removed: 3298 myosin motor domains

dataset6

Dataset5 was taken and all orphan myosins removed: 3104 myosin motor domains

dataset7

Dataset6 was taken and the 5 lophotrochozoan orphan myosins, which are Myo12 candidates, were added: 3109 myosin motor domains

dataset8

Dataset7 was taken and all (also partials to include all the basal grouping myosins) class-7, class-9, class-10, class-15 and class-22 myosins, and the AcMyo_A (*Acanthamoeba castellanii*) and the AopMyo_A (*Amoebidium parasiticum*) orphan myosins were added: 3552 myosin motor domains

dataset9

Dataset8 was taken and the Panagroleimoidea RhabMyo15 (*Rhabditophanes* sp. KR3021), PrtMyo15 (*Parastrongyloides trichosuri*), StrMyo15 (*Strongyloides ratti*), StpaMyo15 (*Strongyloides papillosus*) and StsMyo15 (*Strongyloides stercoralis*), and the TecMyo15 (contains only tail sequence) were removed: 3546 myosin motor domains

dataset10

Dataset9 was taken and all class-17 myosins were removed: 3332 myosin motor domains

1.18. Domain abbreviations

C1, protein kinase C conserved region 1; CBS, cystathionine-beta-synthase; CH, Calponin homology domain; Cyt-b5, cytochrome b5-like Heme/Steroid binding domain; DIL, dilute; FERM, band 4.1, ezrin, radixin, and moesin; FYVE, zinc finger in Fab1, YOTB/ZK632.12, Vac1, and EEA1; GAF, domain present in phytochromes and cGMP-specific phosphodiesterases; IQ motif, isoleucine-glutamine motif; MyTH1, myosin tail homology 1; MyTH4, myosin tail homology 4; PB1, Phox and Bem1p domain; PDZ, PDZ domain; PH, pleckstrin homology; Pkinase, protein kinase domain; PX, phox domain; RA, Ras association (RalGDS/AF-6) domain; RCC1, regulator of chromosome condensation; RhoGAP, Rho GTPase-activating protein; RhoGEF, Rho GDP/GTP exchange factor; SAM, sterile alpha motif; SH2, src homology 2; SH3, src homology 3; UBA, ubiquitin associated domain; WD40, WD (tryptophan-aspartate) or beta-transducin repeats; WW, tryptophan-tryptophan motif domain.

2. References

1. Foth BJ, Goedecke MC, Soldati D. New insights into myosin evolution and classification. Proc Natl Acad Sci U S A. 2006;103:3681–6.

2. Hodge T, Cope MJ. A myosin family tree. J Cell Sci. 2000;113 Pt 19:3353–4.

3. Odronitz F, Kollmar M. Drawing the tree of eukaryotic life based on the analysis of 2,269 manually annotated myosins from 328 species. Genome Biol. 2007;8:R196.

4. Richards TA, Cavalier-Smith T. Myosin domain evolution and the primary divergence of eukaryotes. Nature. 2005;436:1113–8.

5. Sebé-Pedrós A, Grau-Bové X, Richards TA, Ruiz-Trillo I. Evolution and classification of myosins, a paneukaryotic whole-genome approach. Genome Biol Evol. 2014;6:290–305.

6. Korn ED. Coevolution of head, neck, and tail domains of myosin heavy chains. Proc Natl Acad Sci U S A. 2000;97:12559–64.

7. Eckert C, Hammesfahr B, Kollmar M. A holistic phylogeny of the coronin gene family reveals an ancient origin of the tandem-coronin, defines a new subfamily, and predicts protein function. BMC Evol Biol. 2011;11:268.

8. Hammesfahr B, Kollmar M. Evolution of the eukaryotic dynactin complex, the activator of cytoplasmic dynein. BMC Evol Biol. 2012;12:95.

9. Kollmar M. Fine-Tuning Motile Cilia and Flagella: Evolution of the Dynein Motor Proteins from Plants to Humans at High Resolution. Mol Biol Evol. 2016;33:3249–67.

10. Kollmar M, Lbik D, Enge S. Evolution of the eukaryotic ARP2/3 activators of the WASP family: WASP, WAVE, WASH, and WHAMM, and the proposed new family members WAWH and WAML. BMC Res Notes. 2012;5:88.

11. Putnam NH, Srivastava M, Hellsten U, Dirks B, Chapman J, Salamov A, et al. Sea anemone genome reveals ancestral eumetazoan gene repertoire and genomic organization. Science. 2007;317:86–94.

12. Yang Y, Xiong J, Zhou Z, Huo F, Miao W, Ran C, et al. The genome of the myxosporean Thelohanellus kitauei shows adaptations to nutrient acquisition within its fish host. Genome Biol Evol. 2014;6:3182–98.

13. del Campo J, Sieracki ME, Molestina R, Keeling P, Massana R, Ruiz-Trillo I. The others: our biased perspective of eukaryotic genomes. Trends Ecol Evol. 2014;29:252–9.

14. Kollmar M, Kollmar L, Hammesfahr B, Simm D. diArk – the database for eukaryotic genome and transcriptome assemblies in 2014. Nucleic Acids Res. 2015;43:D1107–12.

15. Kollmar M, Hatje K. Shared Gene Structures and Clusters of Mutually Exclusive Spliced Exons within the Metazoan Muscle Myosin Heavy Chain Genes. PLoS ONE. 2014;9:e88111.

16. Odronitz F, Becker S, Kollmar M. Reconstructing the phylogeny of 21 completely sequenced arthropod species based on their motor proteins. BMC Genomics. 2009;10:173.

17. Findeisen P, Mühlhausen S, Dempewolf S, Hertzog J, Zietlow A, Carlomagno T, et al. Six Subgroups and Extensive Recent Duplications Characterize the Evolution of the Eukaryotic Tubulin Protein Family. Genome Biol Evol. 2014;6:2274–88.

18. Mühlhausen S, Kollmar M. Whole genome duplication events in plant evolution reconstructed and predicted using myosin motor proteins. BMC Evol Biol. 2013;13:202.

19. Lu Q, Li J, Ye F, Zhang M. Structure of myosin-1c tail bound to calmodulin provides insights into calcium-mediated conformational coupling. Nat Struct Mol Biol. 2015;22:81–8.

20. Kollmar M. Thirteen is enough: the myosins of Dictyostelium discoideum and their light chains. BMC Genomics. 2006;7:183.

21. Hirano Y, Hatano T, Takahashi A, Toriyama M, Inagaki N, Hakoshima T. Structural basis of cargo recognition by the myosin-X MyTH4-FERM domain. EMBO J. 2011;30:2734–47.

22. Wu L, Pan L, Wei Z, Zhang M. Structure of MyTH4-FERM domains in myosin VIIa tail bound to cargo. Science. 2011;331:757–60.

23. Van de Peer Y, Maere S, Meyer A. 2R or not 2R is not the question anymore. Nat Rev Genet. 2010;11:166.

24. Steinke D, Hoegg S, Brinkmann H, Meyer A. Three rounds (1R/2R/3R) of genome duplications and the evolution of the glycolytic pathway in vertebrates. BMC Biol. 2006;4:16.

25. Berthelot C, Brunet F, Chalopin D, Juanchich A, Bernard M, Noël B, et al. The rainbow trout genome provides novel insights into evolution after whole-genome duplication in vertebrates. Nat Commun. 2014;5:3657.

26. Gillespie PG, Albanesi JP, Bahler M, Bement WM, Berg JS, Burgess DR, et al. Myosin-I nomenclature. J Cell Biol. 2001;155:703–4.

27. Hofmann WA, Richards TA, de Lanerolle P. Ancient animal ancestry for nuclear myosin. J Cell Sci. 2009;122 Pt 5:636–43.

28. Yoon SJ, Seiler SH, Kucherlapati R, Leinwand L. Organization of the human skeletal myosin heavy chain gene cluster. Proc Natl Acad Sci U S A. 1992;89:12078–82.

29. Ikeda D, Ono Y, Snell P, Edwards YJK, Elgar G, Watabe S. Divergent evolution of the myosin heavy chain gene family in fish and tetrapods: evidence from comparative genomic analysis. Physiol Genomics. 2007;32:1–15.

30. Wolfe KH, Shields DC. Molecular evidence for an ancient duplication of the entire yeast genome. Nature. 1997;387:708–13.

31. Lenassi M, Gostinčar C, Jackman S, Turk M, Sadowski I, Nislow C, et al. Whole genome duplication and enrichment of metal cation transporters revealed by de novo genome

sequencing of extremely halotolerant black yeast Hortaea werneckii. PloS One. 2013;8:e71328.

32. Ma L-J, Ibrahim AS, Skory C, Grabherr MG, Burger G, Butler M, et al. Genomic analysis of the basal lineage fungus Rhizopus oryzae reveals a whole-genome duplication. PLoS Genet. 2009;5:e1000549.

33. Aury J-M, Jaillon O, Duret L, Noel B, Jubin C, Porcel BM, et al. Global trends of whole-genome duplications revealed by the ciliate Paramecium tetraurelia. Nature. 2006;444:171–8.

34. Martens C, Vandepoele K, Peer YV de. Whole-genome analysis reveals molecular innovations and evolutionary transitions in chromalveolate species. Proc Natl Acad Sci. 2008;105:3427–32.

35. Hatje K, Hammesfahr B, Kollmar M. WebScipio: Reconstructing alternative splice variants of eukaryotic proteins. Nucleic Acids Res. 2013;41 Web Server issue:W504-509.

36. Mühlhausen S, Hellkamp M, Kollmar M. GenePainter v. 2.0 resolves the taxonomic distribution of intron positions. Bioinformatics. 2015;31:1302–4.

37. Katoh K, Standley DM. MAFFT multiple sequence alignment software version 7: improvements in performance and usability. Mol Biol Evol. 2013;30:772–80.

38. Liu K, Linder CR, Warnow T. RAxML and FastTree: Comparing Two Methods for Large-Scale Maximum Likelihood Phylogeny Estimation. PLoS ONE. 2011;6.

39. Arner A, Löfgren M, Morano I. Smooth, slow and smart muscle motors. J Muscle Res Cell Motil. 2003;24:165–73.

40. Grewal PK, Jones AM, Maconochie M, Lemmers RJ, Frants RR, Hewitt JE. Cloning of the murine unconventional myosin gene Myo9b and identification of alternative splicing. Gene. 1999;240:389–98.

41. Mercer JA, Seperack PK, Strobel MC, Copeland NG, Jenkins NA. Novel myosin heavy chain encoded by murine dilute coat colour locus. Nature. 1991;349:709–13.

42. Nal N, Ahmed ZM, Erkal E, Alper OM, Lüleci G, Dinç O, et al. Mutational spectrum of MYO15A: the large N-terminal extension of myosin XVA is required for hearing. Hum Mutat. 2007;28:1014–9.

43. Weil D, Levy G, Sahly I, Levi-Acobas F, Blanchard S, El-Amraoui A, et al. Human myosin VIIA responsible for the Usher 1B syndrome: a predicted membrane-associated motor protein expressed in developing sensory epithelia. Proc Natl Acad Sci U S A. 1996;93:3232–7.

44. Odronitz F, Kollmar M. Pfarao: a web application for protein family analysis customized for cytoskeletal and motor proteins (CyMoBase). BMC Genomics. 2006;7:300.

3. Supplementary Figures

A Assembly and alignment of newly identified myosins

1. Obtain the genomic region of a putative myosin gene according to TBLASTN results, see example below
2. A) Data from related species not available: Submit genomic region to gene prediction programs (e.g. AUGUSTUS, Genscan)
   B) Data from related species available: Use myosins from related species as query in WebScipio
3. Take predicted myosin sequence and BLASTP against CyMoBase`s myosins to determine closest homolog in existing data
4. Pre-align predicted myosin sequence against closest myosin homolog with ClustalW
5. Evaluate entire aligned sequence for gene prediction problems (e.g. exonic region missing, intronic region mispredicted as exon, wrong splice sites, genome assembly problems), see Fig. S2 for an example
6. Manually adjust the pre-aligned sequence against the alignment of all myosins

B Identification of new myosins: e.g. TBLASTN in Symbiodinium minutum (Dinophyceae)

query sequence:
motor domain of DdMhc (*Dictyostelium discoideum*)

query sequence:
motor domain of TgMyo55 (*Toxoplasma gondii*)

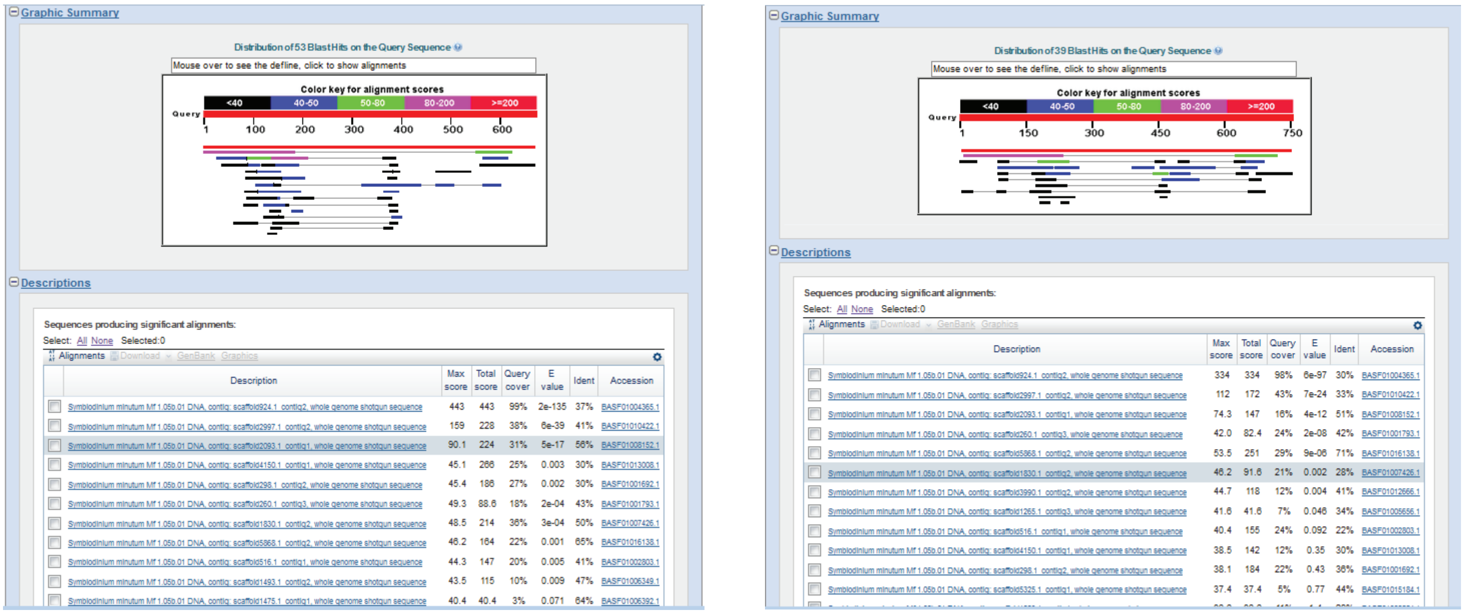

Although this is not at all obvious from these BLAST searches, *S.minutum* contains **at least 18 myosins** (see Fig. S3)!

C Number of identified and reconstructed myosins per class and species

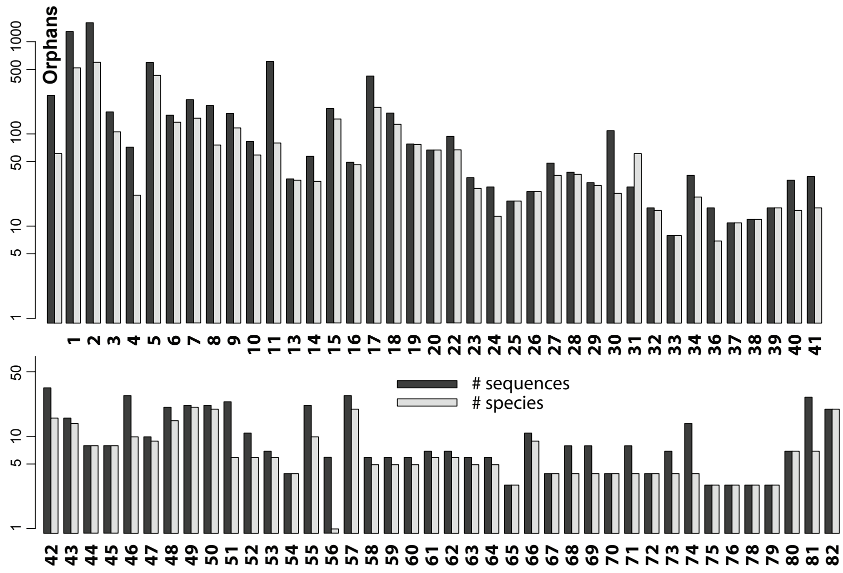

Fig. S1: Myosin identification and sequence assembly process. A) Short overview of the myosin identification, assembly and alignment process. B) The examples show that many TBLASTN searches have to be performed for each species using different myosins as query sequence, because more divergent myosins might not be revealed by a single TBLASTN search. C) Distribution of the identified and assembled myosins across classes.

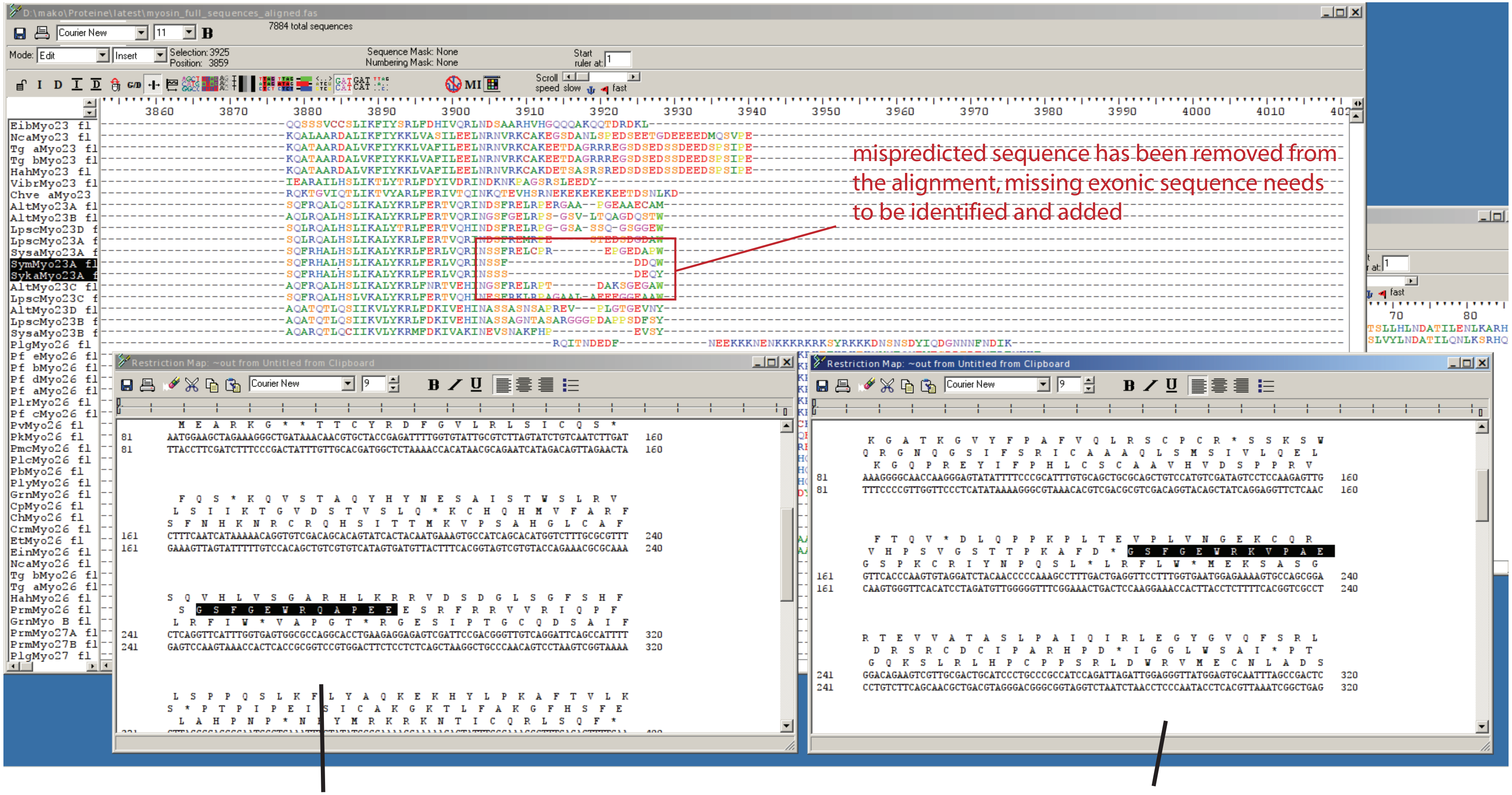

Fig. S2: Identification of exons by comparing three-reading-frame translations of genomic regions of related species. Gene predictions have been obtained for the Myo23A homologs of *Symbiodinium minutum* and *Symbiodinium kawagutii*. These were aligned to the likely closest related sequence (the Myo23A homolog from *Symbiodinium sp. A1*, which was obtained from TSA data) and obviously wrong sequence removed. This status is shown in the screenshot of the alignment. Note, the removed sequences do not correspond to any gene structure information, the deletion is performed just based on protein sequence similarity and thus further sequences might be deleted from the edges of the gap to rebuild correct splice sites. Subsequently, the corresponding genomic regions of the two genes were obtained and translated into all 3 reading frames (shown in the two windows at the bottom). The *S.minutum* genomic region is shorter than the corresponding region of *S.kawagutii* facilitating the identification of potential exons, for which homologous regions were subsequently be searched for in the *S.kawagutii* genomic region. The highlighted regions are homologous in both species and homologous to the corresponding sequences of other Myo23 myosins (see alignment). To correctly build a gene structure, the "SF" and "SS" amino acids on the right side of the gap in the alignment need to be removed and the "G" in the highlighted regions are not part of the final sequence because the intron splits the codon, which codes for Ser in the spliced DNA. Exon identification in *Symbiodinium* is considerably complicated because of the extensive use of GA---AG intron splice sites in these species.

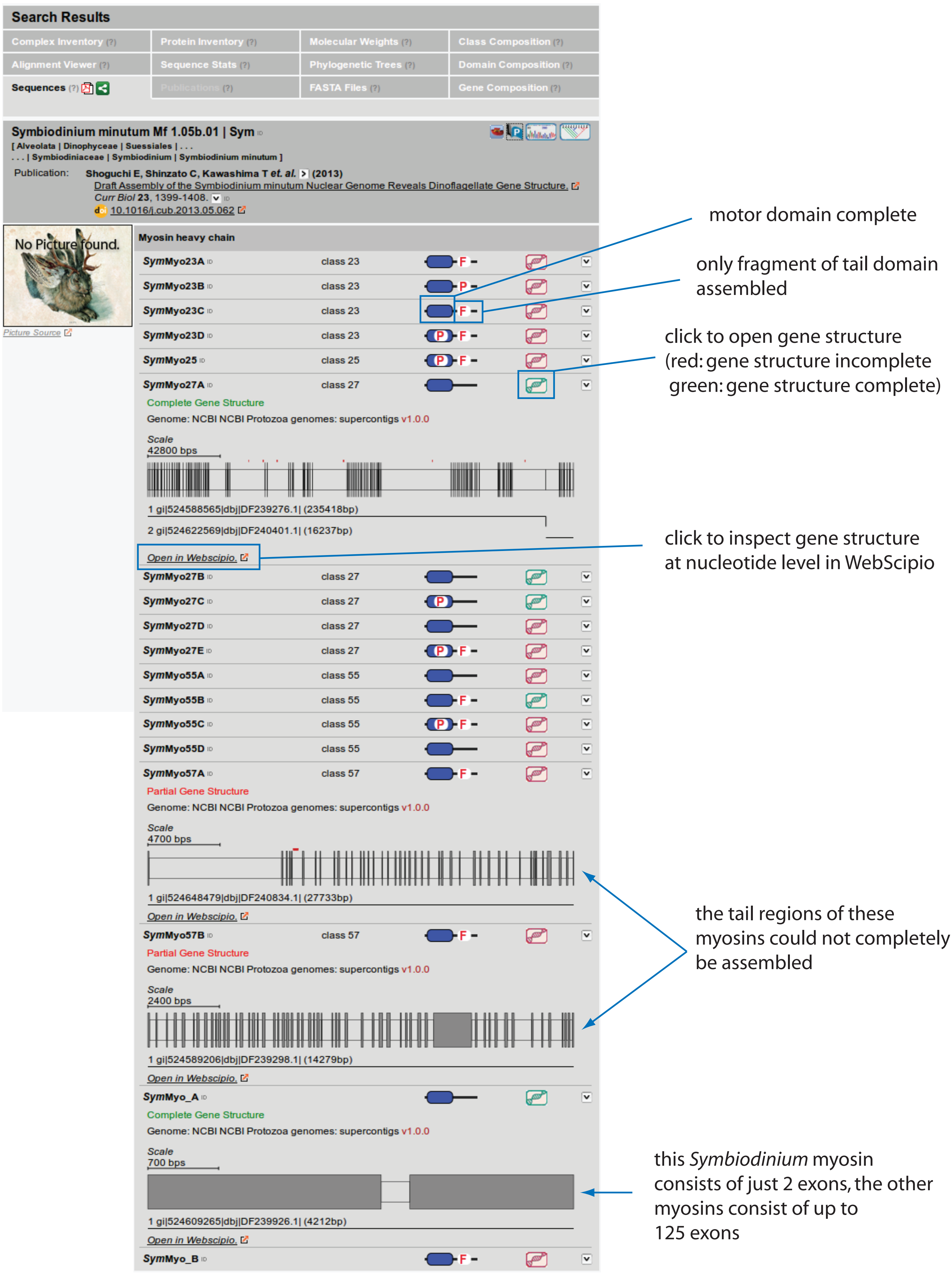

Fig. S3: Sequence Result View of CyMoBase showing the 18 *Symbiodinium minutum* myosins. Several of the gene structures have been opened to demonstrate the complexity of the gene structures, which is not visible from the BLAST result. The tail regions are very divergent and could only partly be assembled.

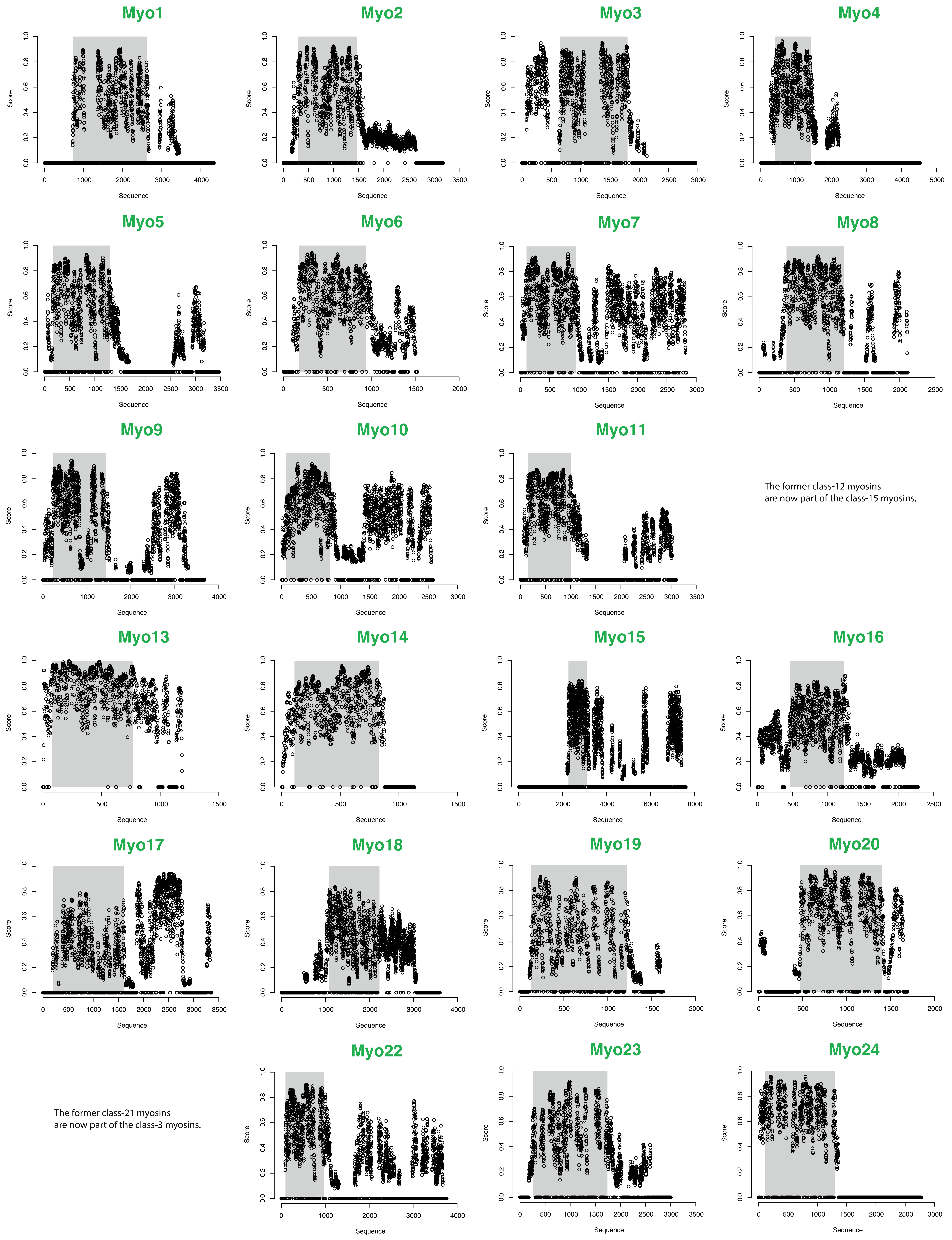
Myo1
Myo2
Myo3
Myo4
Myo5
Myo6
Myo7
Myo8
Myo9
Myo10
Myo11
The former class-12 myosins
are now part of the class-15 myosins.
Myo13
Myo14
Myo15
Myo16
Myo17
Myo18
Myo19
Myo20
The former class-21 myosins
are now part of the class-3 myosins.
Myo22
Myo23
Myo24
Score
Sequence

Myo25

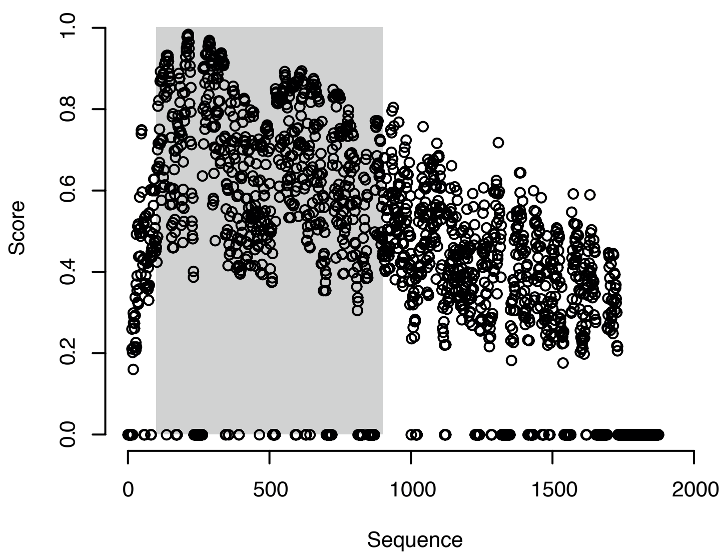

Myo26

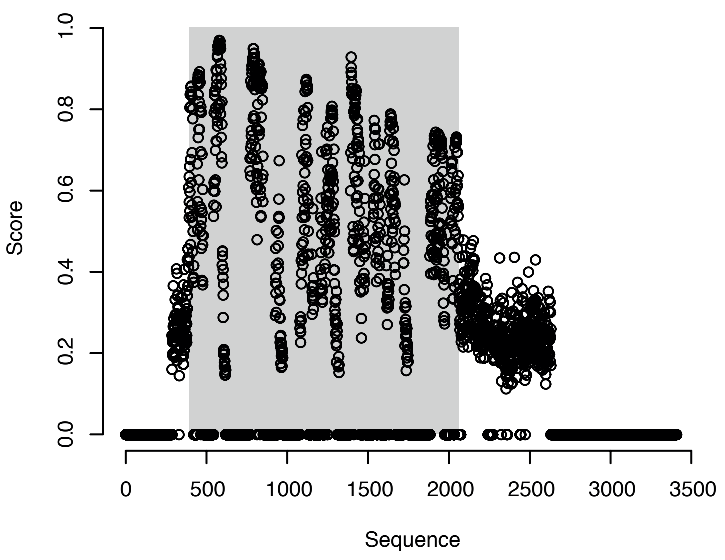

Myo27

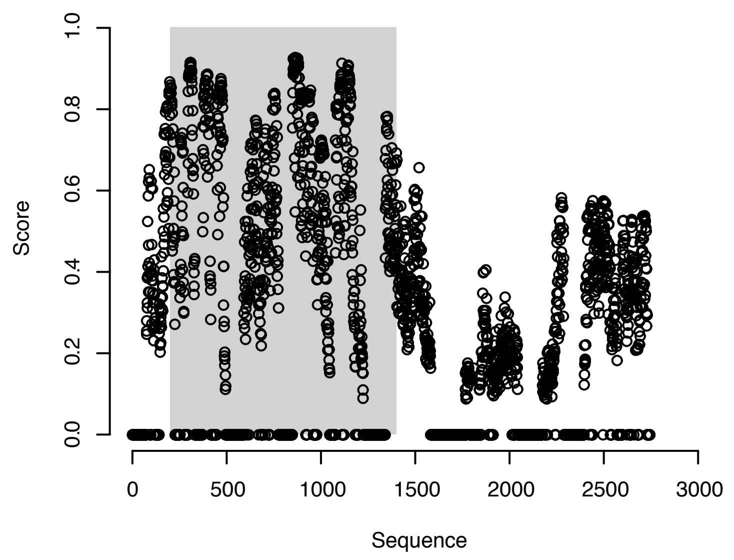

Myo28

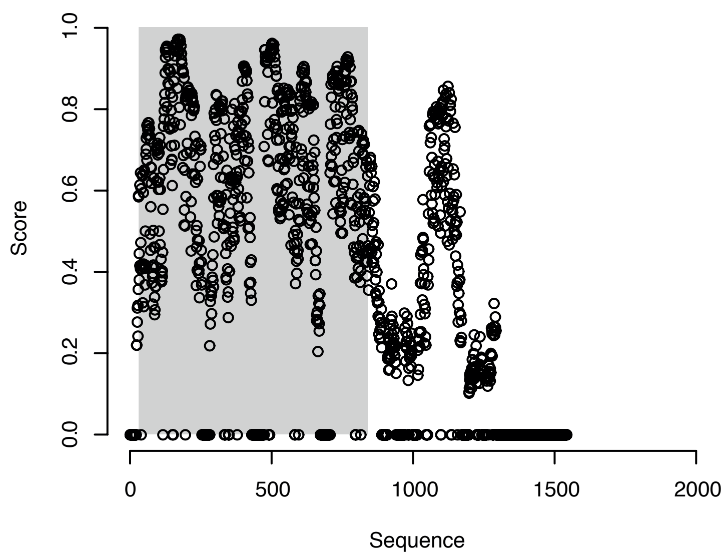

Myo29

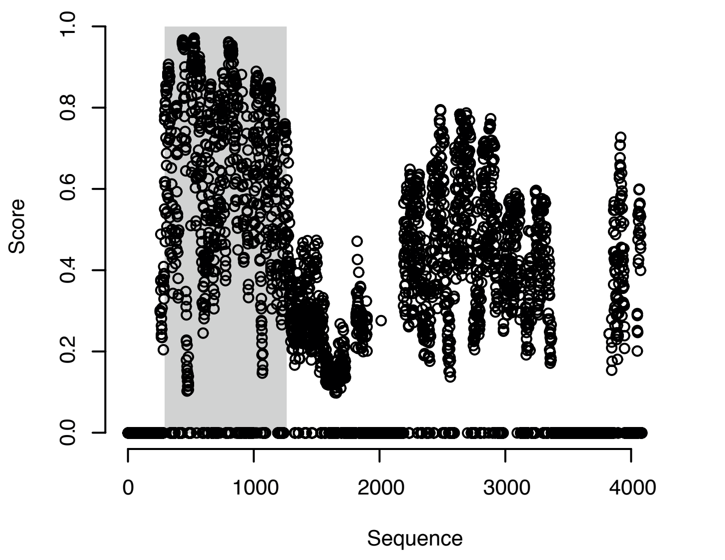

Myo30

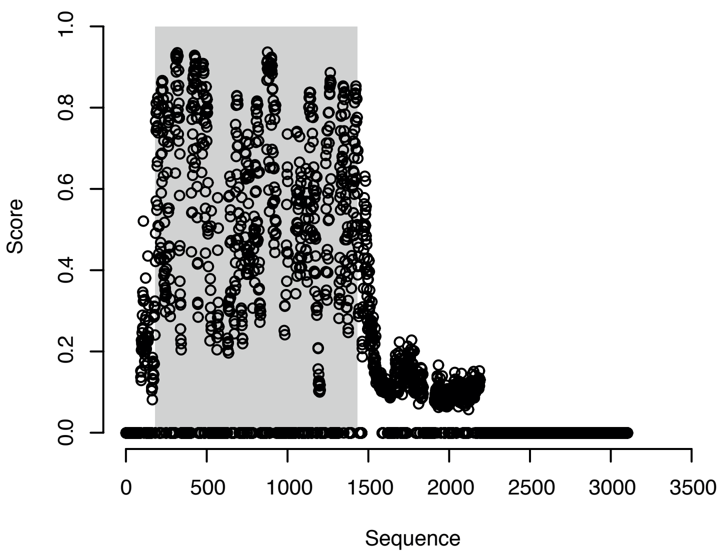

Myo31

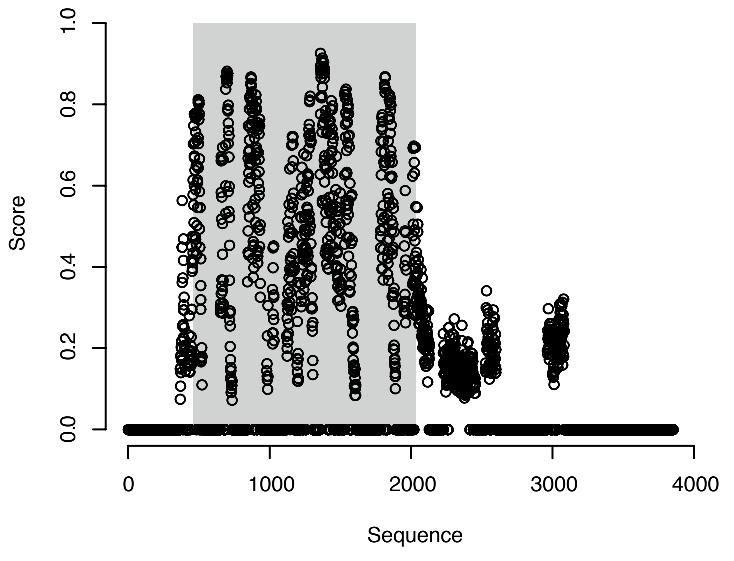

Myo32

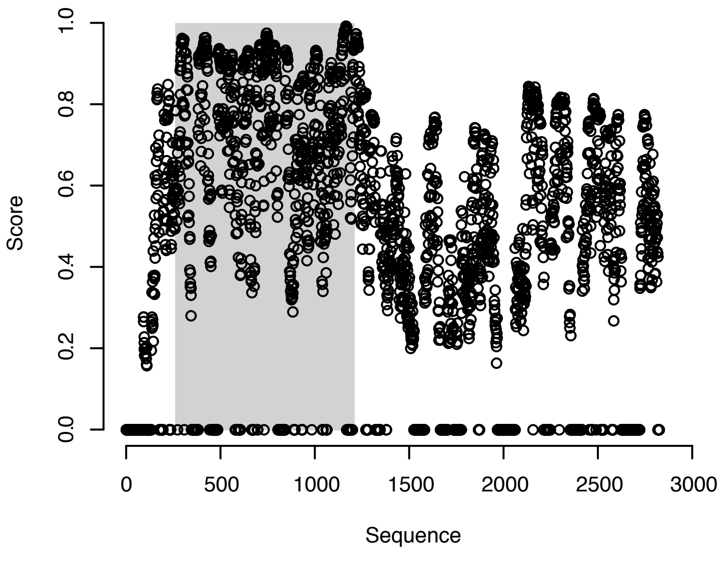

Myo33

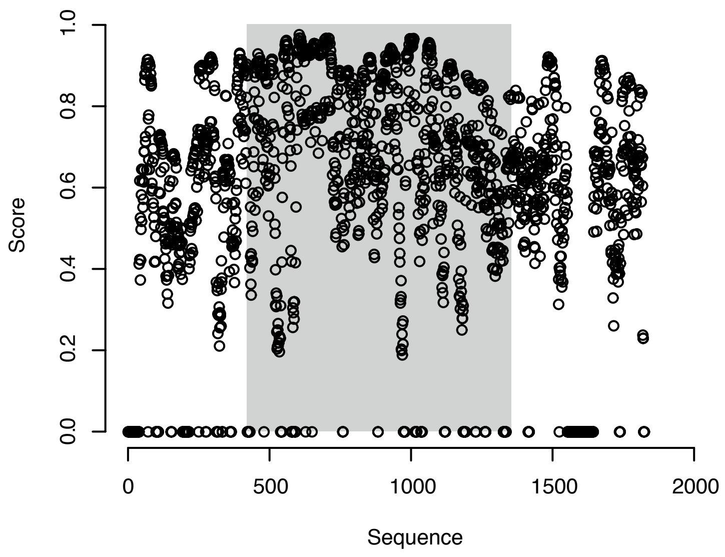

Myo34

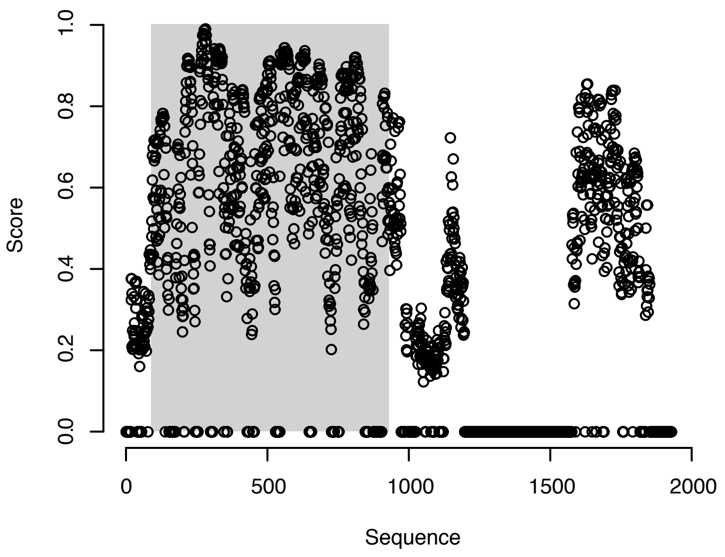

The former class-35 myosins
are now part of the class-15 myosins.

Myo36

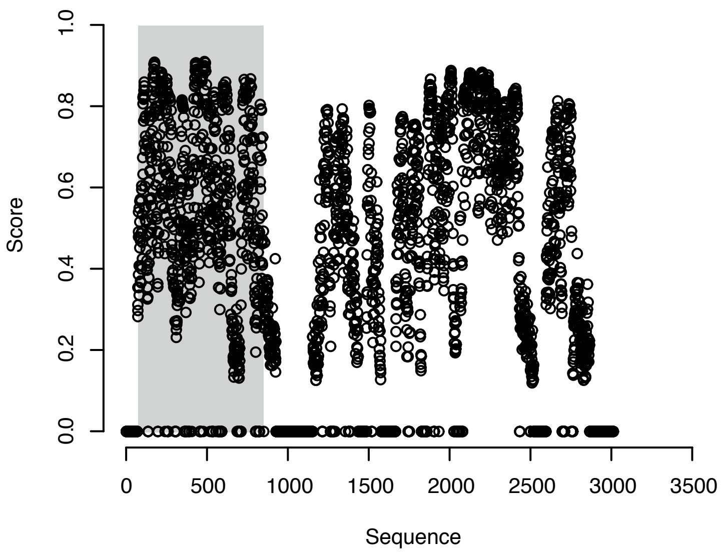

Myo37

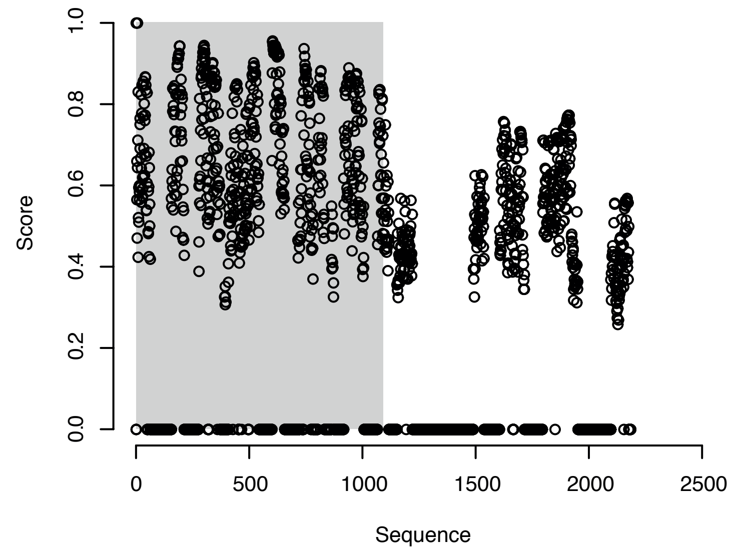

Myo38

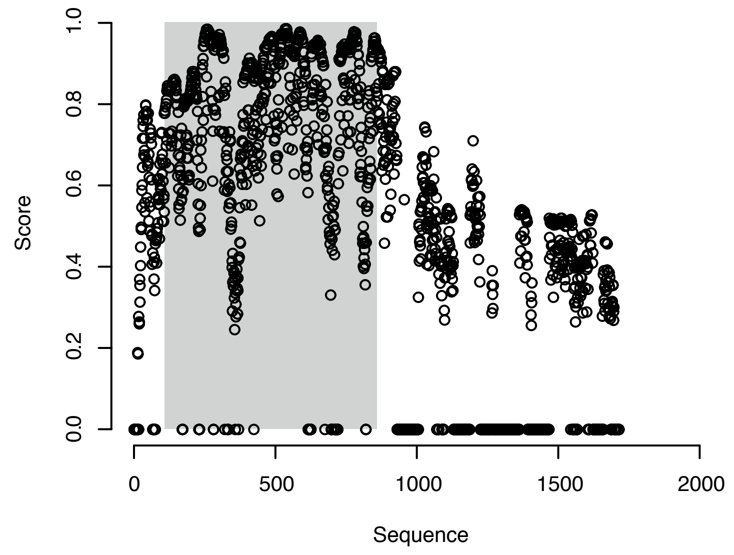

Myo39

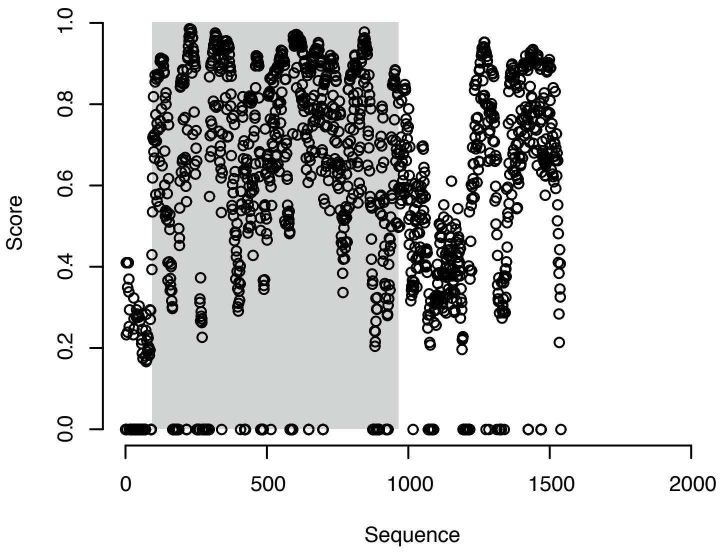

Myo40

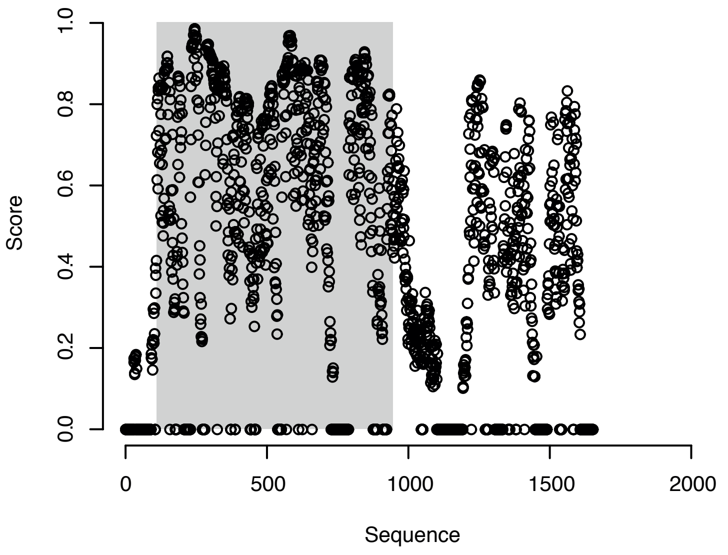

Myo41

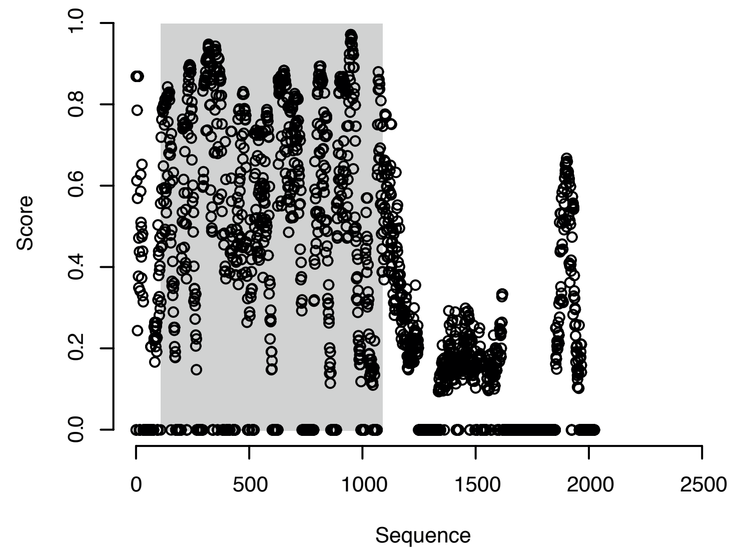

Myo42

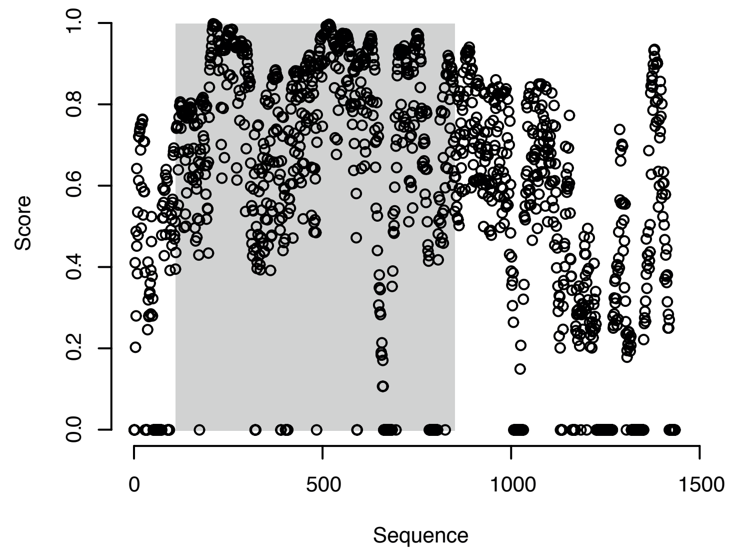

Myo43

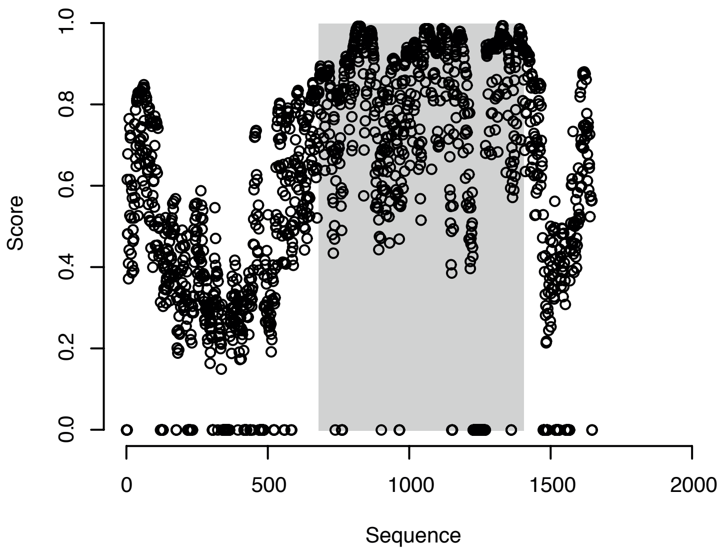

Myo44

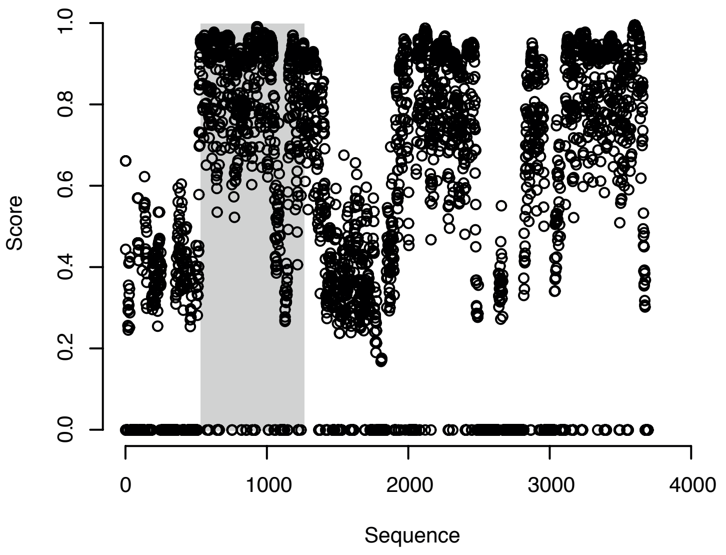

Myo45

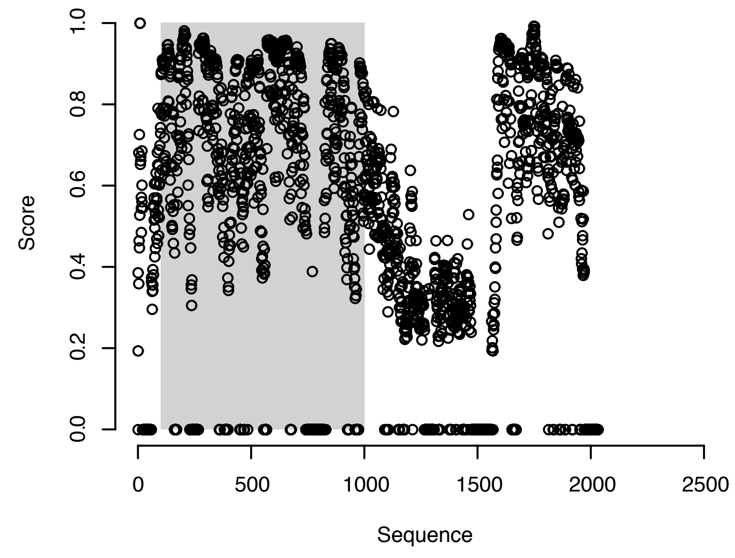

Myo46

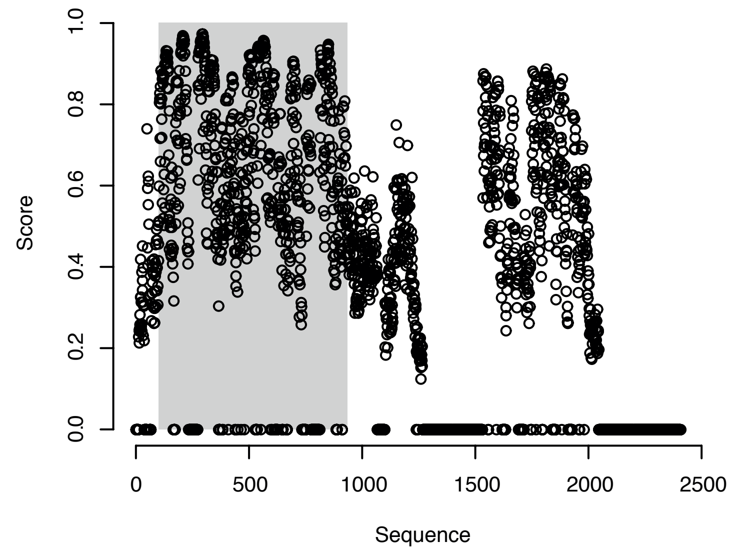

Myo47

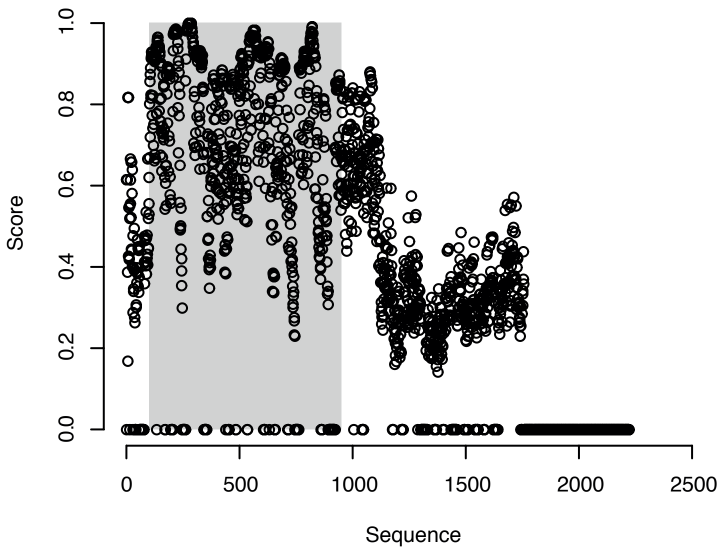

Myo48

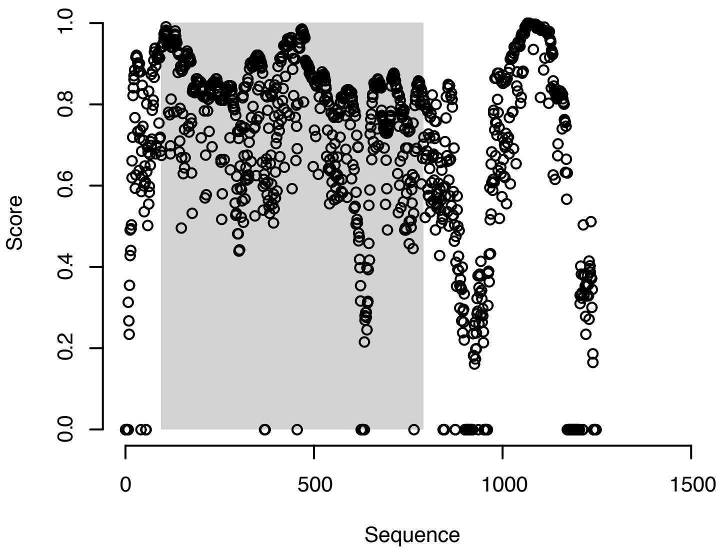

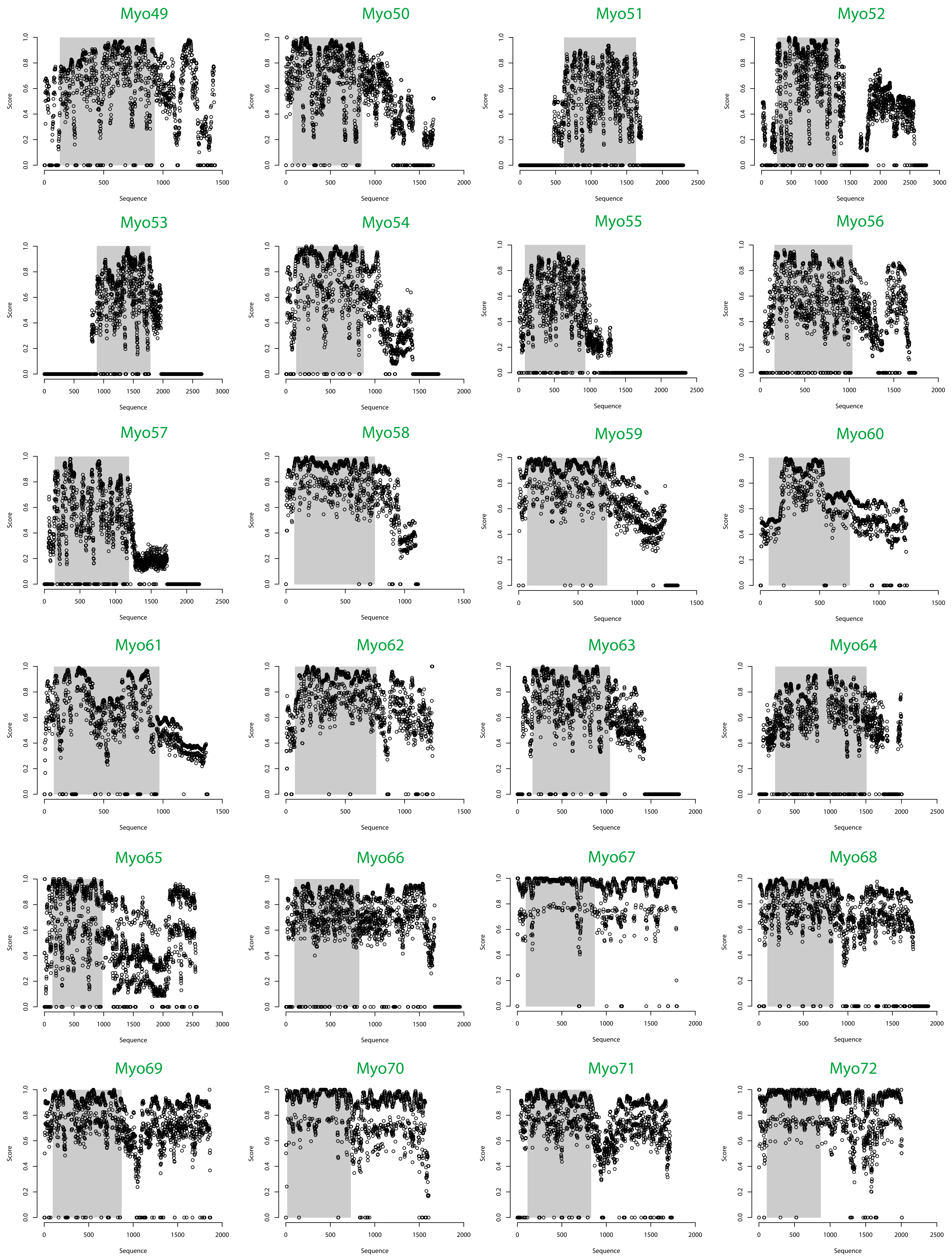
Myo49
Myo50
Myo51
Myo52
Myo53
Myo54
Myo55
Myo56
Myo57
Myo58
Myo59
Myo60
Myo61
Myo62
Myo63
Myo64
Myo65
Myo66
Myo67
Myo68
Myo69
Myo70
Myo71
Myo72
Score
Sequence

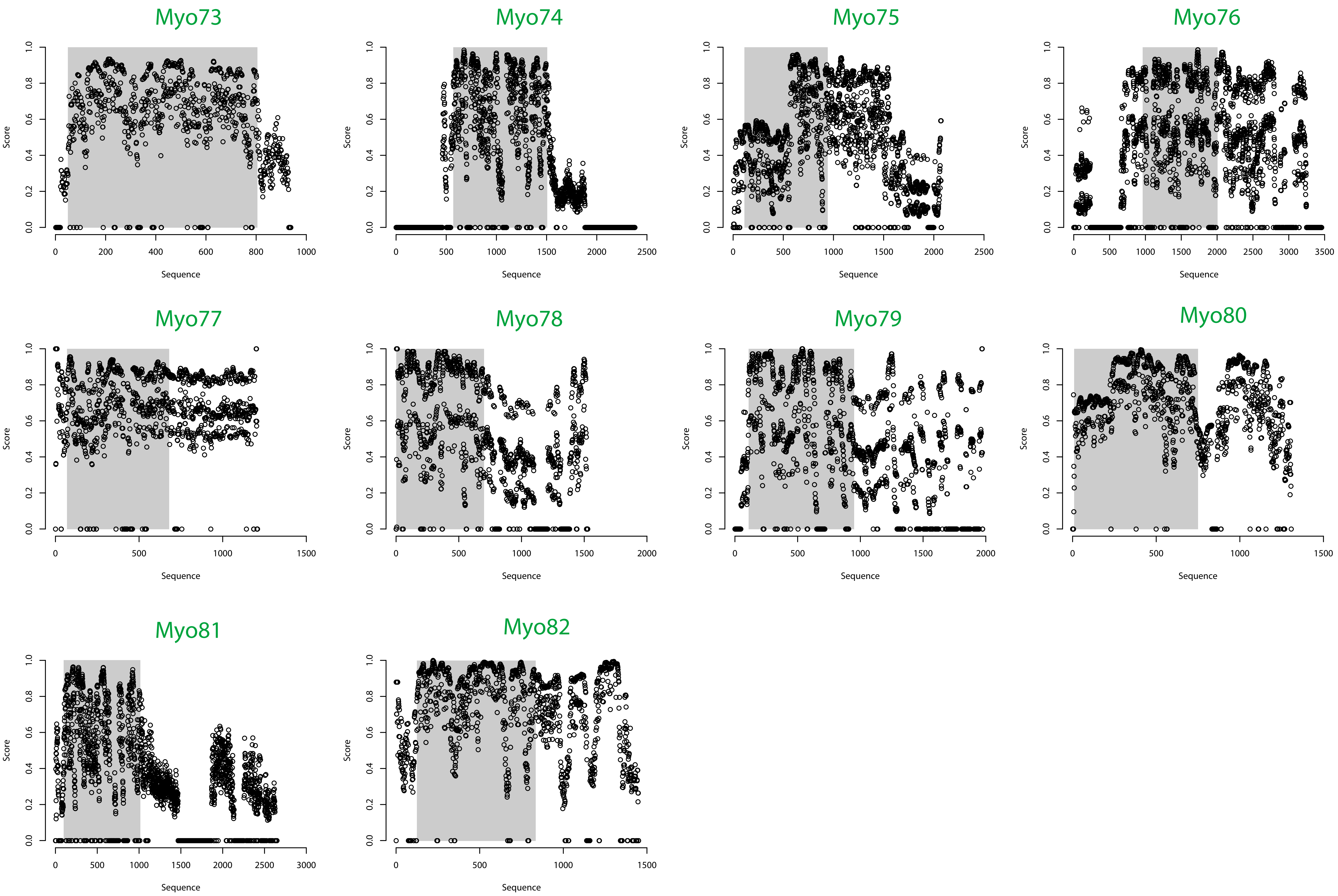

Fig. S4: Sequence conservation within myosin classes. Sequence conservation was calculated for each class separately. The residue conservation at alignment positions was calculated with the conservation code toolbox as implemented by (Capra and Singh 2007). Conservation was estimated with the property-entropy method, an entropy measurement refined with respect to chemical properties of amino acids. Scores were calculated with conservation of adjacent amino acids incorporated (window size 10). Except for window size and scoring method, standard parameters were used. Amino acids given as "X" are replaced by hyphens "-" by the software, which denote gap positions in the alignment. The myosin motor domain regions are indicated by grey areas.

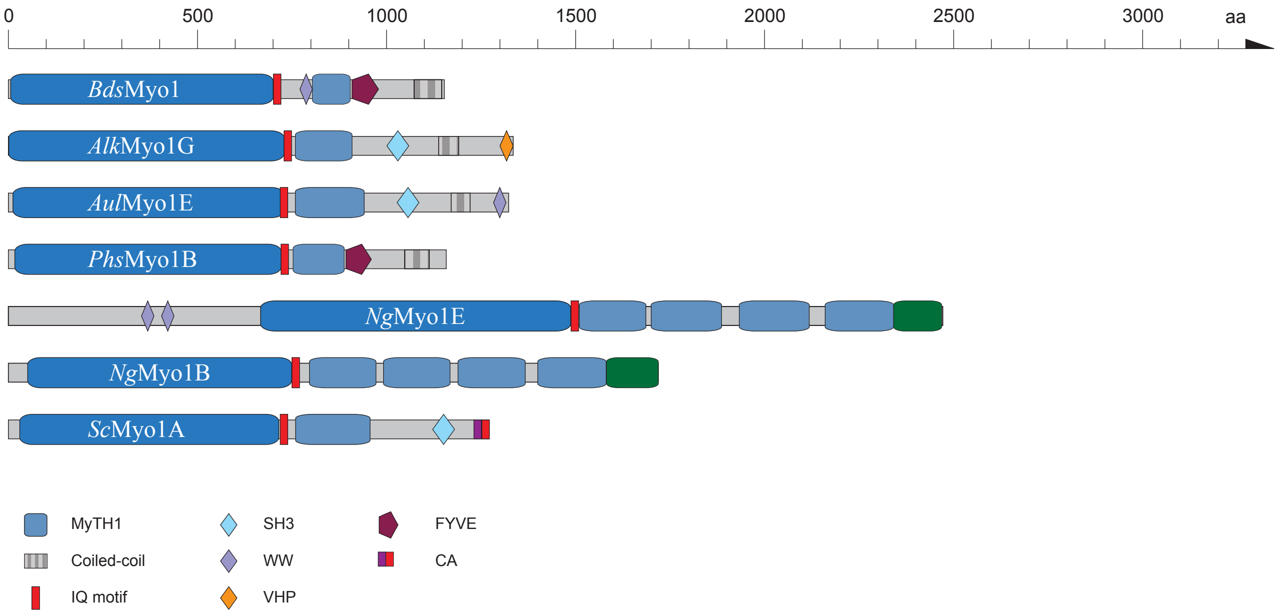

Fig. S5: Myosin-1 domain architecture diversity. The scheme shows examples of class-1 myosins with domain architectures deviating from the domain architecture shown in Fig. 2, which is representative for metazoan class-1 myosins. *Bodo saltans* has been chosen as representative for kinetoplastids, *Aplanochytrium kerguelense* and *Aurantiochytrium limacinum* as representatives for Labrinthulomycetes, *Phytophthora sojae* as representative for Oomycota, *Naegleria gruberi* as representative for Heterolobosea, and *Saccharomyces cerevisiae* as representative for fungi. The sequence name of the representative class-1 myosin is given in the motor domain of the respective myosin.
Regions not having assigned a defined domain do not necessarily indicate variable regions but rather missing domain definitions and might be highly conserved within the respective proteins. A color key to the domain names and symbols is given at the bottom.

The abbreviations for the domains are:
CA, central acidic; FYVE, zinc finger in Fab1, YOTB/ZK632.12, Vac1, and EEA1;
IQ motif, isoleucine-glutamine motif; MyTH1, myosin tail homology 1; SH3, src homology 3;
VHP, Villin headpiece domain; WW, tryptophan-tryptophan motif domain.

Species abbreviations are:
Alk: *Aplanochytrium kerguelense*; Aul: *Aurantiochytrium limacinum*; Bds: *Bodo saltans*;
Ng: *Naegleria gruberi*; Phs: *Phytophthora sojae*; Sc: *Saccharomyces cerevisiae*.

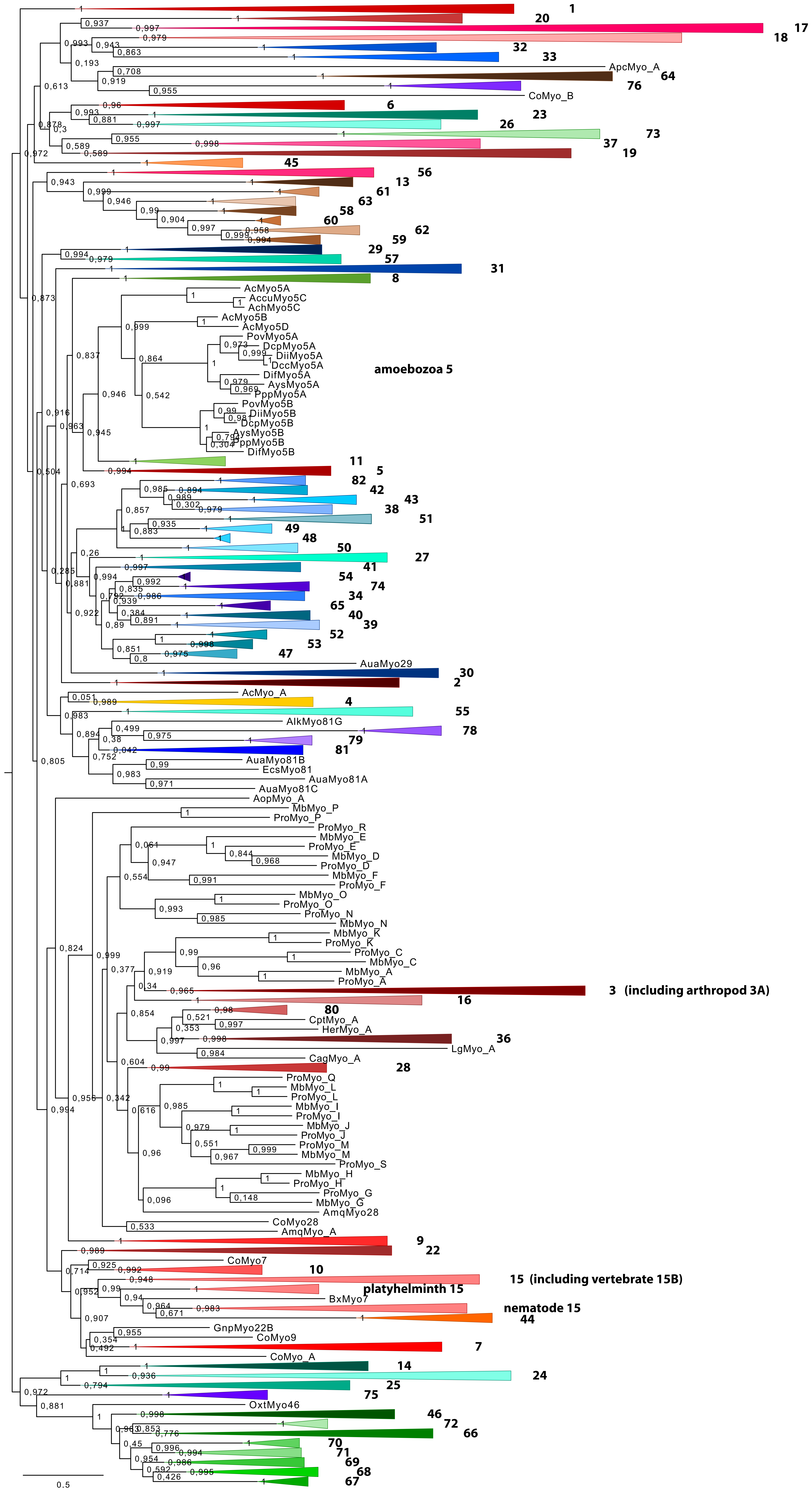

Fig. S6: Example phylogenetic tree of the myosins showing common origin of the class-3 myosins but polyphyly of the class-15 myosins. Maximum-likelihood topology generated under the WAG + Γ model as implemented in FastTree. The tree is based on a dataset of complete myosin motor domains after reducing redundancy (CD-Hit, 90% identity). Subsequently, the divergent class-77, ascomycote class-17B, and Panagrolaimoidea class-15 myosins, as well as all orphan myosins were removed. Then, all class-3, -16, -28, -36, -80, -7, -10, -15 (except Panagrolaimoidea), -22 myosins and all choanoflagellate, ichthyosporean and metazoan orphan myosins were added resulting in 3747 sequences. All branches with unambiguous class members have been collapsed for better presentation. The scale bar represents the estimated number of amino acid substitutions per site. In the phylogenetic tree of this dataset, all class-3 myosins group together, in contrast to the tree shown in Fig. 2, where the arthropod Myo3A myosins group somewhere else in the tree (which is also found in the tree shown in Fig. S7). However, the class-15 myosins appear polyphyletic: the most basal node comprising class-15 myosin also includes a nematode class-7 myosin from *Bursaphelenchus xylophilus* (BxMyo7) and all class-44 myosins. Although the class-44 myosins have a related domain architecture, they group outside the class-15 myosins in all other dataset and were therefore designated an own class. The BxMyo7 myosin, a Tylenchida nematode myosin, is a divergent Myo7 and usually groups within the other class-7 myosins. The vertebrate class-15B myosins (former class-35) group sister to the vertebrate class-15A myosins (not shown in detail here), but the platyhelminthes Myo15 and nematode Myo15 (former class-12) group outside the other class-15 myosins. A different type of Myo15-polyphyly is observed in the example tree shown in Fig. S7 which is based on a different dataset. In the Fig. S7 tree, the platyhelminthes Myo15 group together with the other class-15 myosins, but the vertebrate class-15B myosins group together with the nematode class-15 myosins at a completely different position in the tree.

Species abbreviaions are: Ac: *Acanthamoeba castellanii*; Accu: *Acanthamoeba culbertsoni*; Ach: *Acanthamoeba healyi*; Alk: *Aplanochytrium kerguelense*; Amq: *Amphimedon queenslandica*; Aop: *Amoebidium parasiticum*; Apc: *Aplysia californica*; Aua: *Aureococcus anophagefferens*; Ays: *Acytostelium subglobosum*; Bx: *Bursaphelenchus xylophilus*; Cag: *Crassostrea gigas*; Co: *Capsaspora owczarzaki*; Cpt: *Capitella teleta*; Dcc: *Dictyostelium citrinum*; Dcp: *Dictyostelium purpureum*; Dif: *Dictyostelium fasciculatum*; Dii: *Dictyostelium intermedium*; Ecs: *Ectocarpus siliculosus*; Gnp: *Gonapodya prolifera*; Her: *Helobdella robusta*; Lg: *Lottia gigantea*; Mb: *Monosiga brevicollis*; Pov: *Polysphondylium violaceum*; Ppp: *Polysphondylium pallidum*; Pro: *Proterospongia sp.*.

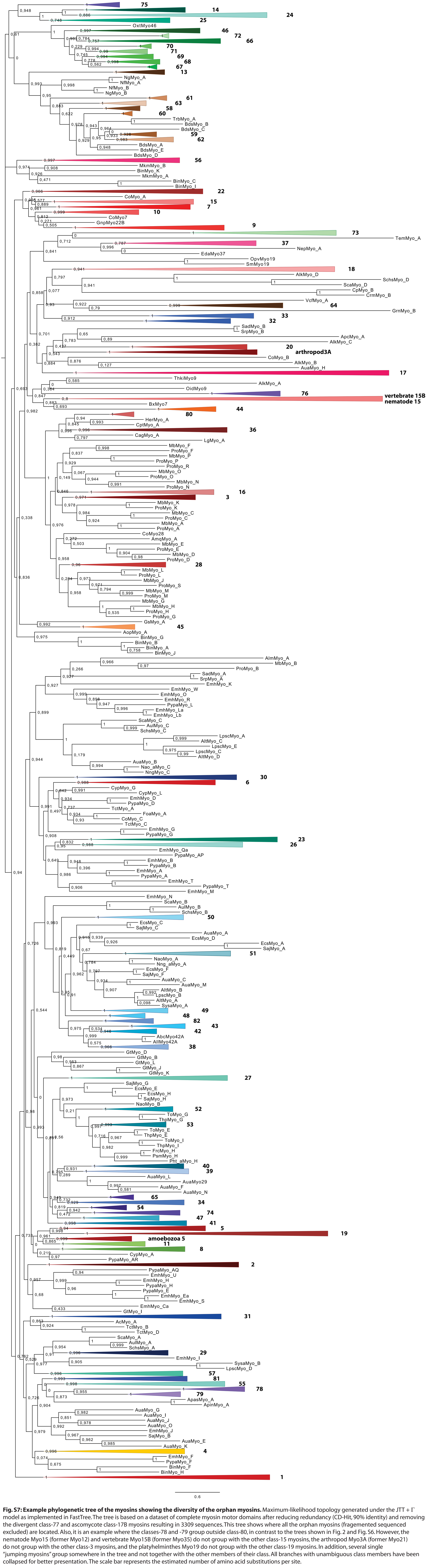

Fig. S7: Example phylogenetic tree of the myosins showing the diversity of the orphan myosins. Maximum-likelihood topology generated under the JTT + Γ model as implemented in FastTree. The tree is based on a dataset of complete myosin motor domains after reducing redundancy (CD-Hit, 90% identity) and removing the divergent class-77 and ascomycote class-17B myosins resulting in 3309 sequences. This tree shows where all the orphan myosins (fragemented sequenced excluded) are located. Also, it is an example where the classes-78 and -79 group outside class-80, in contrast to the trees shown in Fig. 2 and Fig. S6. However, the nematode Myo15 (former Myo12) and vertebrate Myo15B (former Myo35) do not group with the other class-15 myosins, the arthropod Myo3A (former Myo21) do not group with the other class-3 myosins, and the platyhelminthes Myo19 do not group with the other class-19 myosins. In addition, several single “jumping myosins” group somewhere in the tree and not together with the other members of their class. All branches with unambiguous class members have been collapsed for better presentation. The scale bar represents the estimated number of amino acid substitutions per site.

Species abbreviations are: Abc: *Albugo candida*; Ac: *Acanthamoeba castellanii*; Alk: *Aplanochytrium kerguelense*; All: *Albugo laibachii*; Alm: *Allomyces macrogynus*; Alt: *Alexandrium tamarense*; Amq: *Amphimedon queenslandica*; Aop: *Amoebidium parasiticum*; Apas: *Aphanomyces astaci*; Apc: *Aplysia californica*; Apin: *Aphanomyces invadans*; Aua: *Aureococcus anophagefferens*; Bds: *Bodo saltans*; Bin: *Bigelowiella natans*; Bx: *Bursaphelenchus xylophilus*; Cag: *Crassostrea gigas*; Co: *Capsaspora owczarzaki*; Cp: *Cryptosporidium parvum*; Cpt: *Capitella teleta*; Crm: *Cryptosporidium muris*; Cyp: *Cyanophora paradoxa*; Eda: *Edhazardia aedis*; Emh: *Emiliania huxleyi*; Foa: *Fonticula alba*; Gnp: *Gonapodya prolifera*; Grn: *Gregarina niphandrodes*; Gs: *Galdieria sulphuraria*; Gt: *Guillardia theta*; Her: *Helobdella robusta*; Lg: *Lottia gigantea*; Lpsc: *Lingulodinium polyedrum*; Mb: *Monosiga brevicollis*; Mkm: *Mikrocytos mackini*; Nao: *Nannochloropsis oceanica*; Nep: *Nematocida parisii*; Nf: *Naegleria fowleri*; Ng: *Naegleria gruberi*; Nng: *Nannochloropsis gaditana*; Oid: *Oikopleura dioica*; Opv: *Opisthorchis viverrini*; Oxt: *Oxytricha trifallax*; Pht: *Phaeodactylum tricornutum*; Pro: *Proterospongia sp.*; Pypa: *Prymnesium parvum*; Sad: *Saprolegnia diclina*; Saj: *Saccharina japonica*; Sca: *Schizochytrium aggregatum*; Schs: *Schizochytrium sp.*; Sm: *Schistosoma mansoni*; Srp: *Saprolegnia parasitica*; Sysa: *Symbiodinium sp.*; Tct: *Thecamonas trahens*; Tem: *Tremella mesenterica*; Thki: *Thelohanellus kitauei*; Thp: *Thalassiosira pseudonana*; To: *Thalassiosira oceanica*; Trb: *Trypanoplasma borreli*; Vcf: *Vavraia culicis 'floridensis'*.

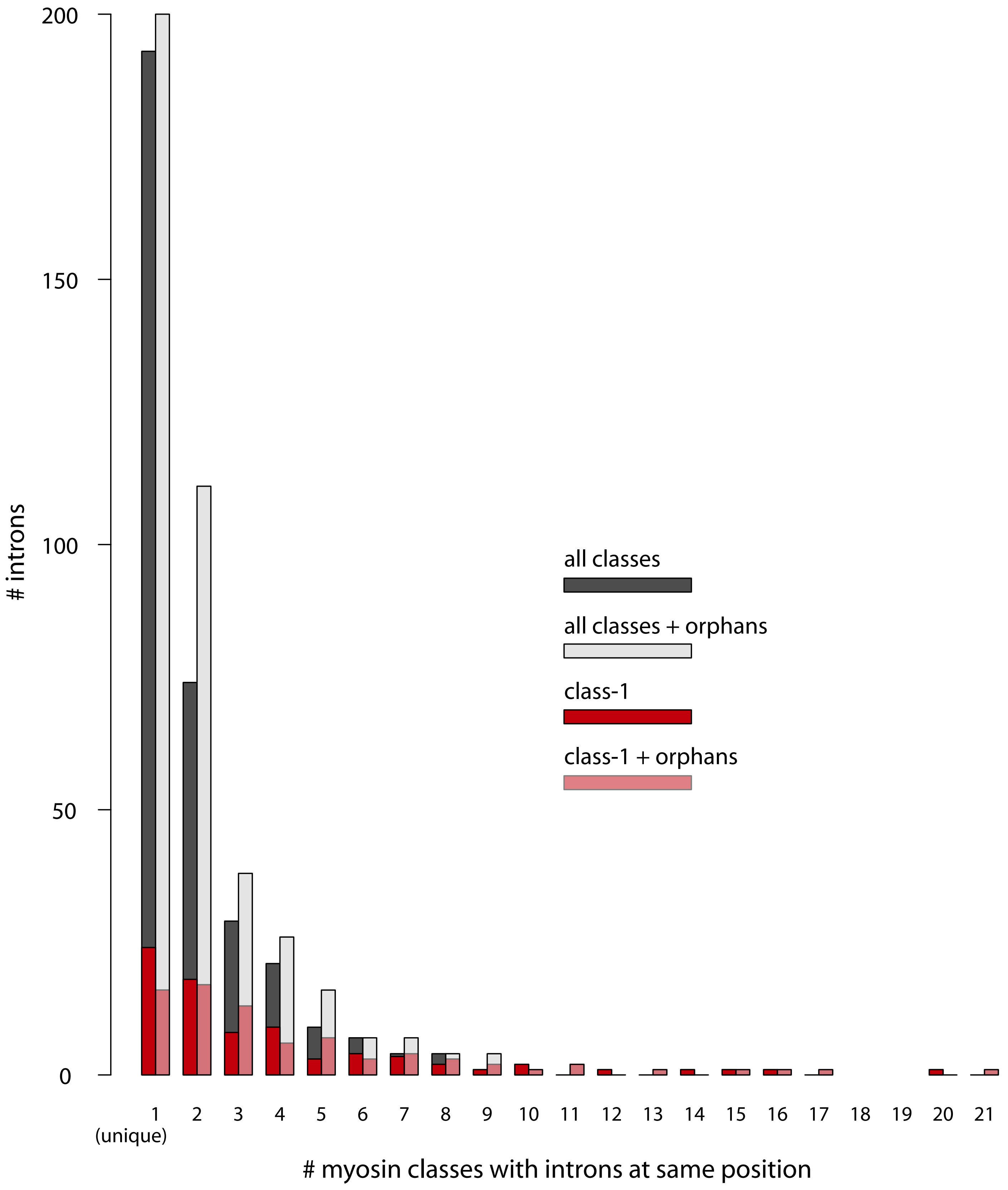

Fig. S8: Intron positions conserved across myosin classes. The bar chart contrasts intron positions conserved in myosin classes with intron positions conserved in myosin classes plus orphans. The comparison shows that most intron positions in orphans are shared with conserved intron positions in myosin classes (see the slight increase in the number of unique intron positions compared to the considerable increase in the numbers of shared intron positions). All introns shared between at least nine classes are always conserved in class-1 myosins. Other classes contain only one or a few but not all of these highly conserved intron positions in their intron position pattern. This together with the high percentage of other intron positions shared between class-1 and other myosins suggests that the ancestor of all myosin classes had an exon-intron pattern closely related to the class-1 intron position pattern.

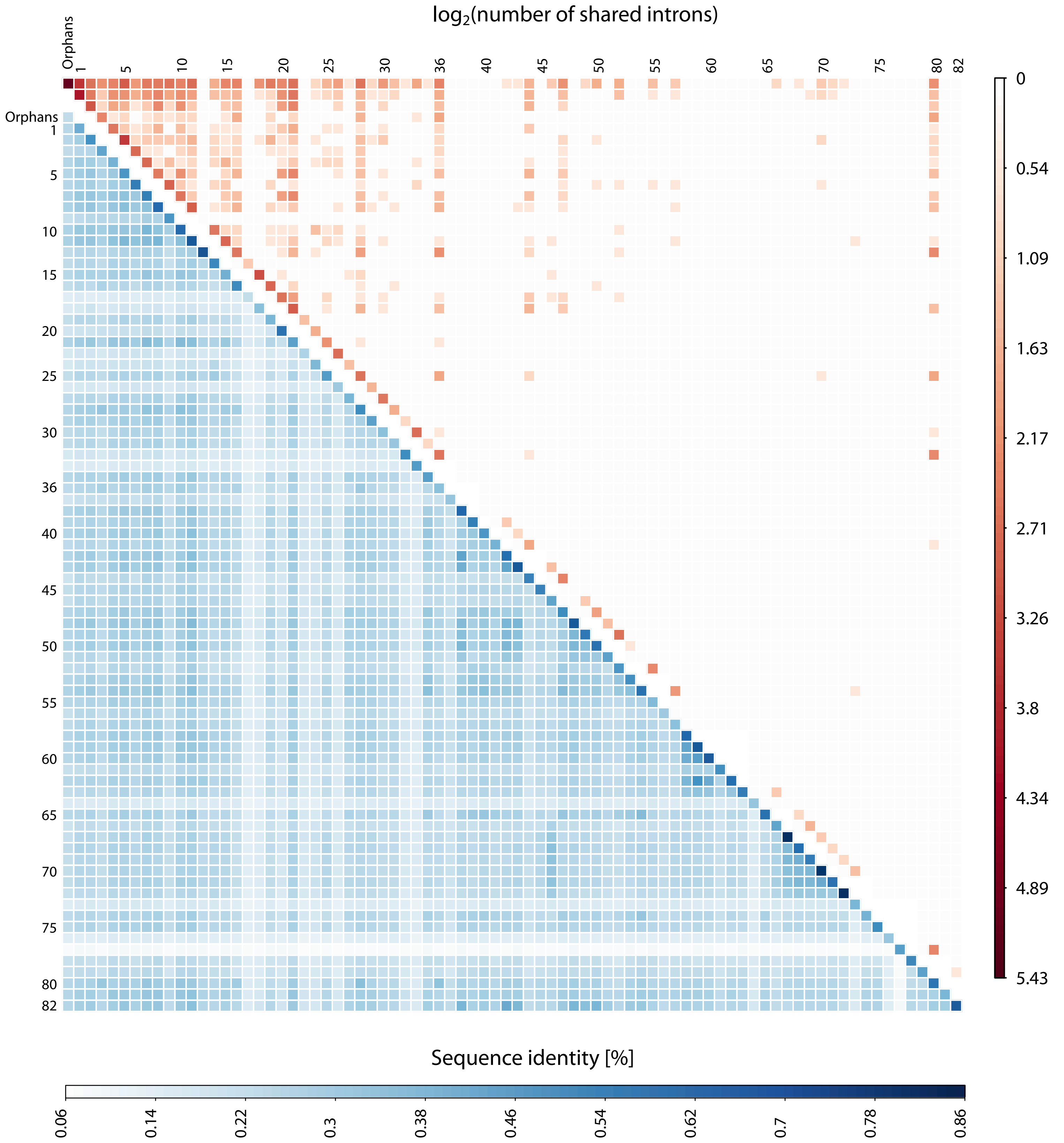

Fig. S9: Intron position conservation and sequence identity. The plot shows the number of conserved introns shared by classes (upper triangle) and the average sequence identity between myosin classes (lower triangle). For computing sequence identities, myosin motor domain sequences designated "Fragment" or "Pseudogene" were removed from the multiple sequence alignment resulting in 7313 sequences. A sequence identity matrix was calculated for the alignment using the method implemented in BioEdit (Tom Hall, http://www.mbio.ncsu.edu/bioedit/bioedit.html). Shortly, the computed numbers represent the ratio of identities to the length of the longer of the two sequences after positions where both sequences contain a gap are removed. The sequence identities were then averaged for each each matrix element. The myosins of the former classes-12, -21, and -35 are now part of other classes, and these class names were not reused.

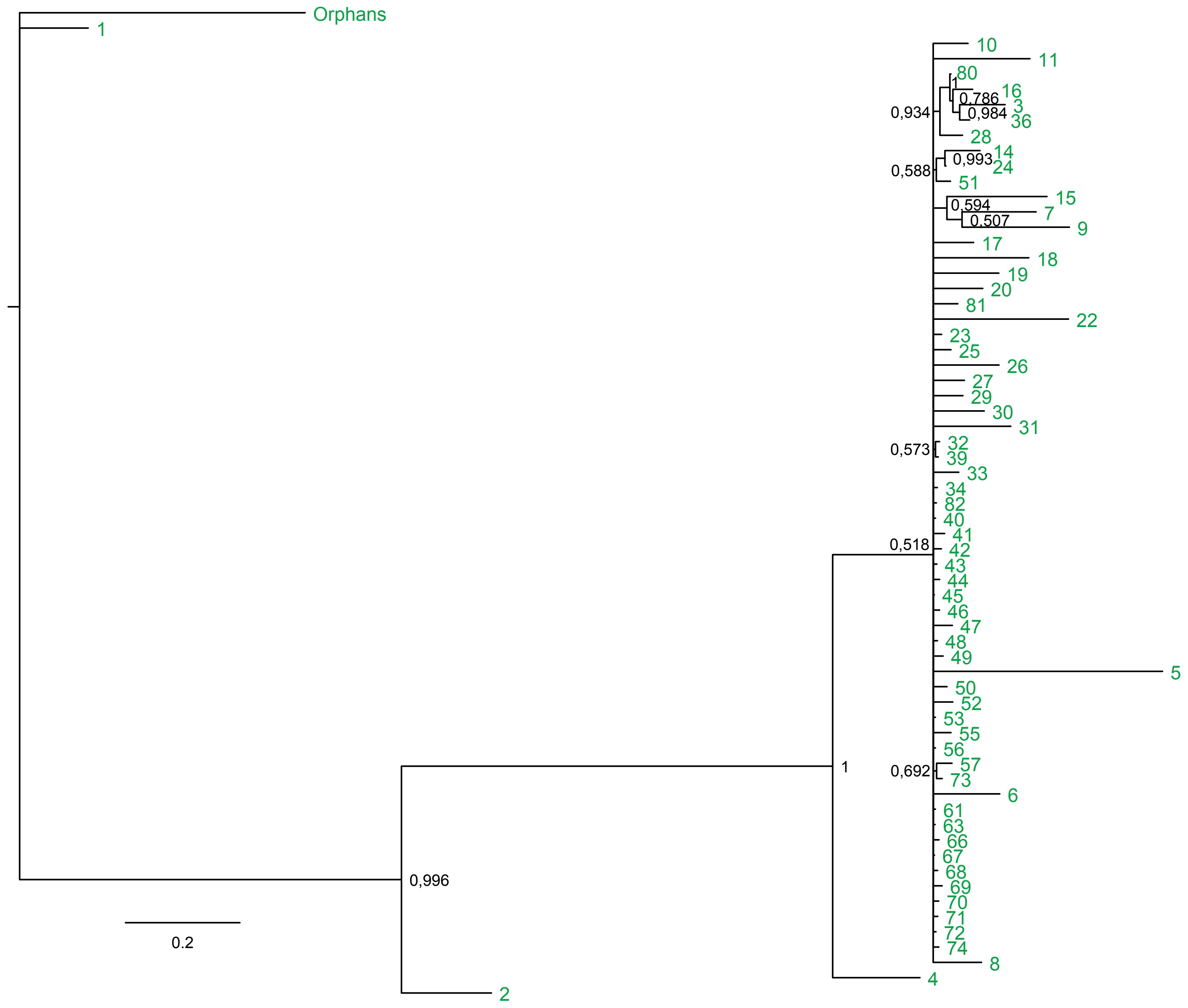

Fig. S10: Phylogeny of the myosin classes based on class-specific intron position patterns. Bayesian tree generated with MrBayes of the intron patterns of the myosin classes. For this analysis, we generated majority intron position patterns for the motor domains of each class. To be able to compare intron position patterns across classes, the motor domain sequences from the full myosin multiple sequence alignment were used. Sequences without introns within the motor domain, and thus also entire classes with myosins not containing any introns were not used in the analysis. As outgroup, we generated an intron position pattern of all orphan myosins. This intron position pattern of the orphan myosins is of course a mixed pattern of potentially 160 classes, spread all over the tree of the eukaryotes. Thus, this intron position pattern shares a few intron positions with almost all classes, but in comparison to every single class the majority of the intron positions are not shared. The tree shows that the intron position patterns do not group into an order of events (e.g. bifurcating nodes) supporting that most have developed independently. Some patterns strongly group together (e.g. class-3, -16, -28, -36, -80) indicating a common origin of these classes, which is also strongly supported by the phylogenetic trees of the motor protein MSAs. The class-1 myosin intron position pattern groups outside all other classes indicating that all other classes originated from class-1.

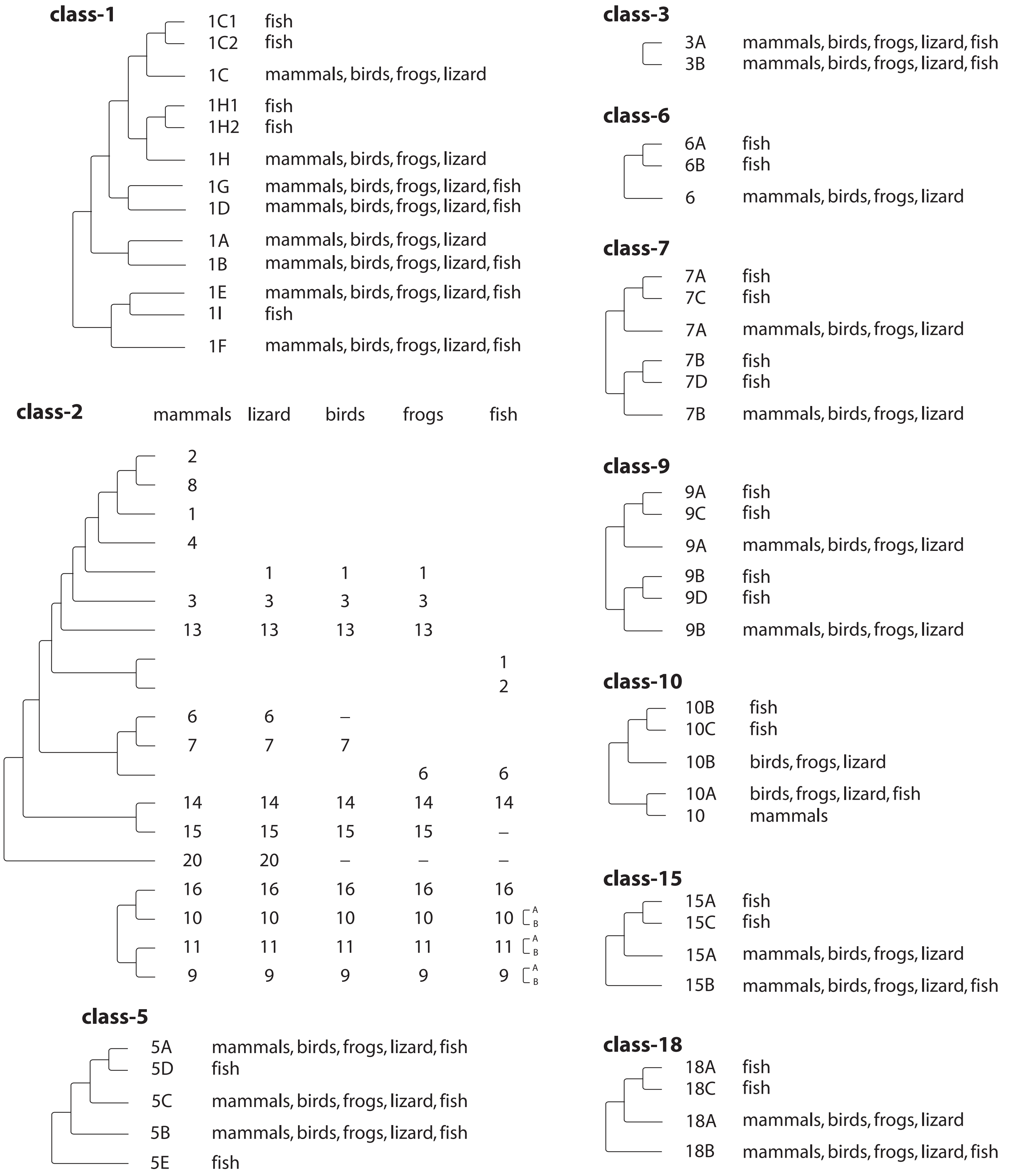

Fig. S11: Vertebrate myosin naming scheme. For each myosin class with duplicates present in vertebrates, the relation of orthologs and paralogs and their proposed naming is shown. Vertebrate class-2 myosins have always been distinguished by numbers, not letters as in the case of the other classes. To distinguish Myo2"1" (class-2 myosin variant "1") from Myo21 (class-21 myosin), the class-2 myosins are abbreviated as Mhc. Accordingly, the numbers in the naming scheme for class-2 myosins denote class-2 myosin variants. Mammalian Mhc1, Mhc2, Mhc4 and Mhc8 are very similar. Because there is no clear phylogenetic grouping of lizard, bird, and frog Mhcs to these variants (in contrast to clear phylogenetic relation to the other variants, e.g. Mhc3 and Mhc13), the corresponding Mhcs were all termed Mhc1 and gene duplicates further distinguished by letters. In case of the fish, there are no clear homologs of mammalian Mhc1, Mhc2, Mhc3, Mhc4, Mhc8 and Mhc13, but two clear subgroups which were termed Mhc1 and Mhc2 (each not related to the mammalian Mhc1 and Mhc2), and further duplicates are distinguished by letters. Dashes indicate absence of respective homologs in these taxa.

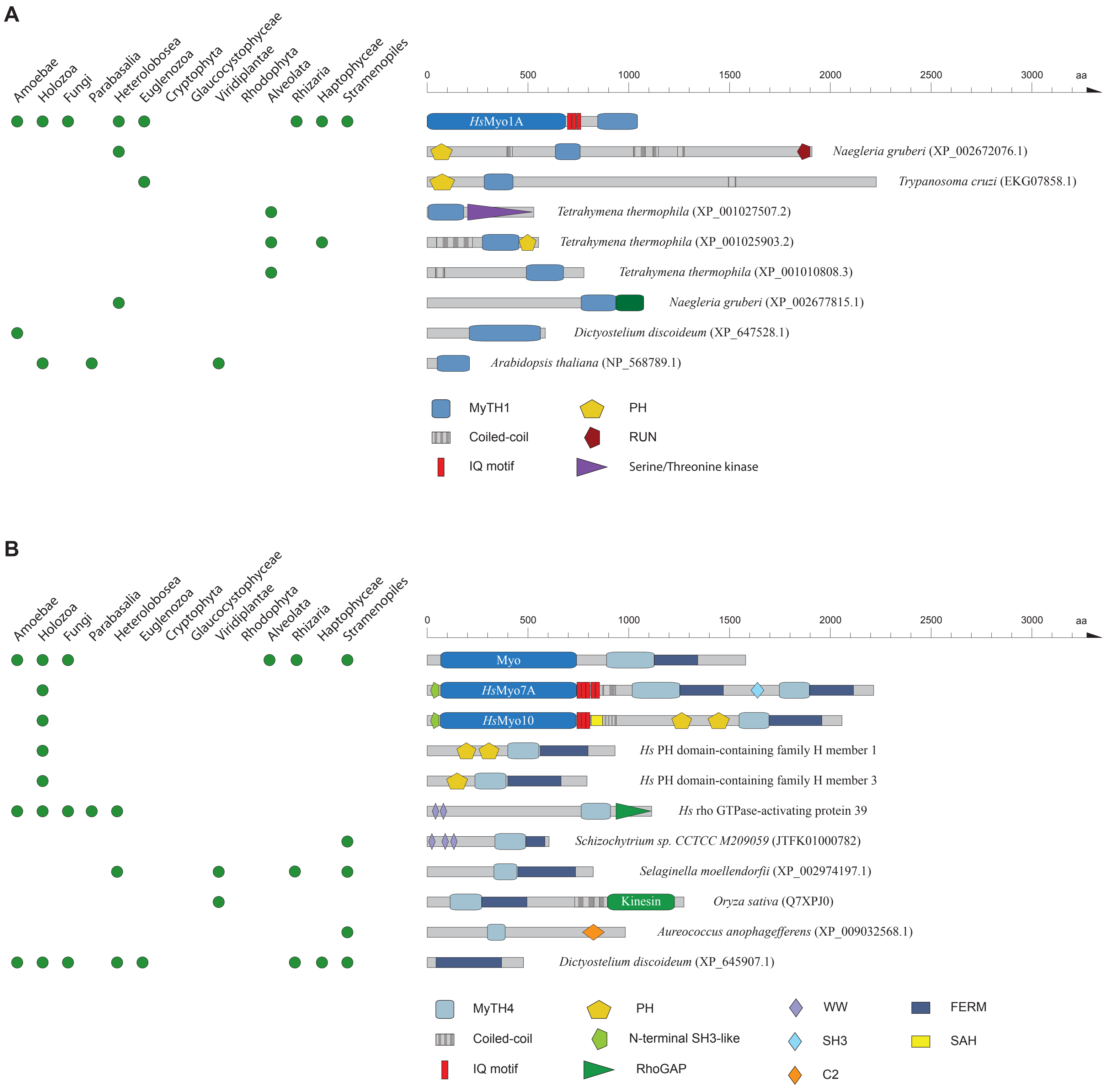

Fig. S12: Diversity and phyolgenetic distribution of selected MyTH1 and MyTH4 domain containing proteins. (A) Domain architecture schemes of representative proteins containing MyTH1 domains, and their phylogenetic distribution. (B) Domain architecture schemes of representative proteins containing MyTH4 domains, and their phylogenetic distribution.

(A) and (B): Regions not having assigned a defined domain do not necessarily indicate variable regions but rather missing domain definitions and might be highly conserved within the respective proteins. A color key to the domain names and symbols is given at the bottom of each panel. The presence of sequences with correponding domain architectures in corresponding taxa is indicated by green dots.

The abbreviations for the domains are:
C2, protein kinase C conserved region 2; FERM, band 4.1, ezrin, radixin, and moesin;
IQ motif, isoleucine-glutamine motif; MyTH1, myosin tail homology 1; MyTH4, myosin tail homology 4;
PH, pleckstrin homology; RhoGAP, Rho GTPase-activating protein; RUN, RPIP8, UNC-14 and NESCA;
SAH, single alpha helix; SH3, src homology 3; WW, tryptophan-tryptophan motif domain.

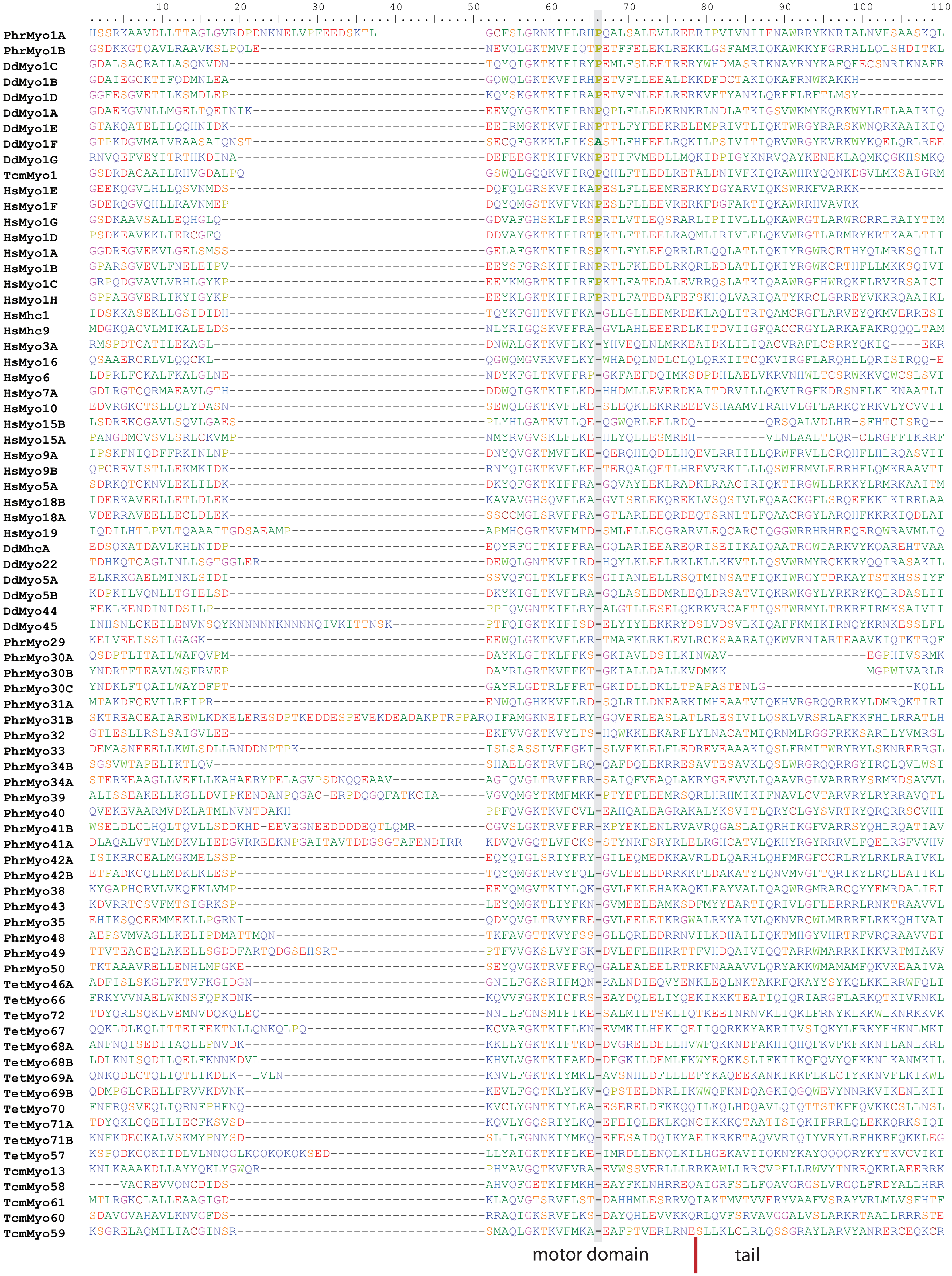

Fig. S13: Sequence alignment focusing around the lever-arm helix and showing representative class-1 myosins and myosins from various classes. The position of the invariant insertion of a single amino acid (mostly proline) in class-1 myosins at the base of the lever is highlighted.

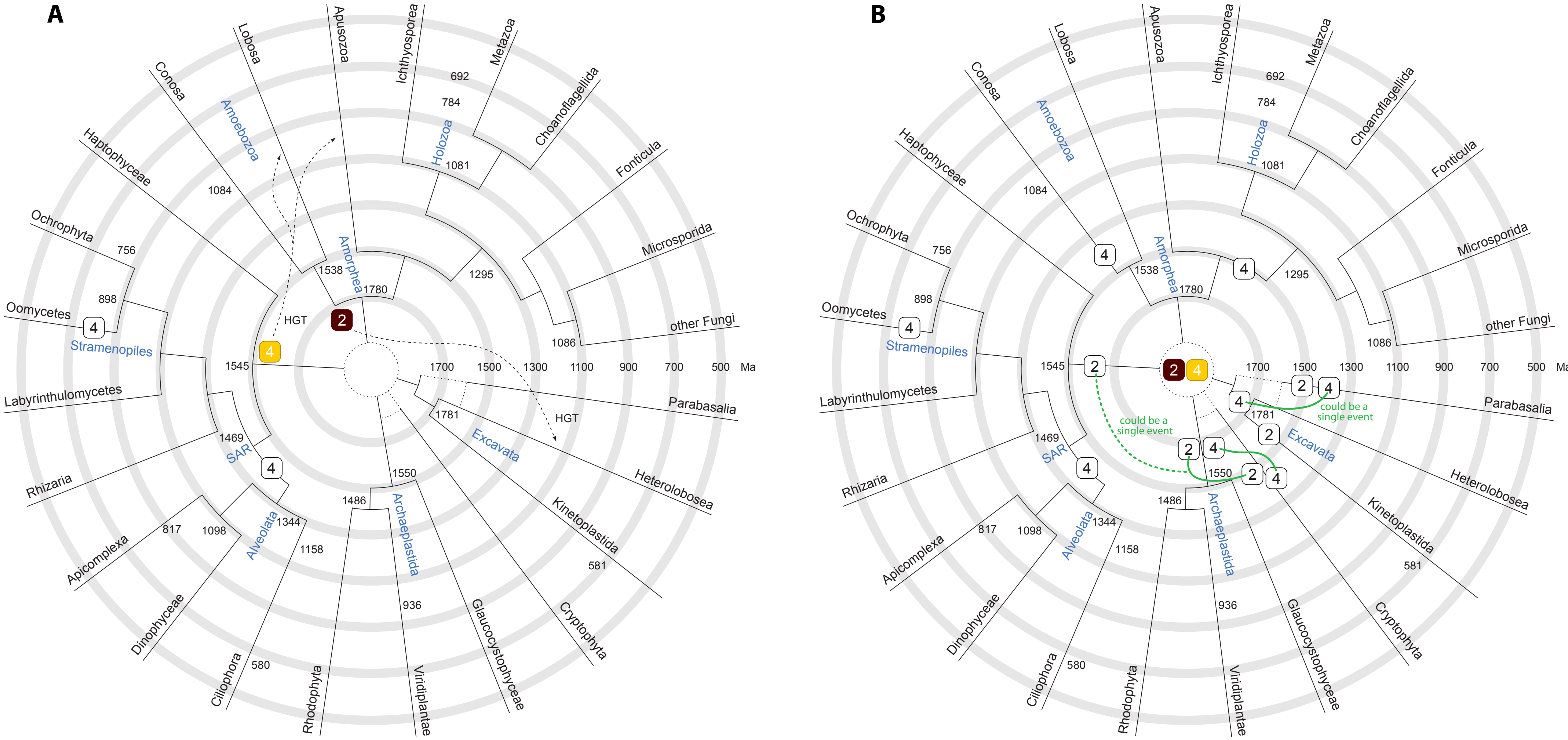

Fig. S14: Contrasting a HGT scenario with a gene loss scenario. This Figure presents the same phylogenetic tree as in Fig. 4. Only class-2 and class-4 gene gain and loss events are shown for clarity. Myosin class inventions are represented by colored boxes and white boxes mark myosin loss events. Controversial branchings are indicated by dotted lines. (A) In the HGT scenario, as shown in Fig. 4, a class-2 myosin would have been gained by the ancestor of the Amorphea. The class-2 myosin found in *Naegleria* species (Heterolobosea) would be gained by a HGT event from an amoebozoan species. This is supported by phylogenetic grouping of the Naegleria class-2 myosins to amoebozoan class-2 myosins. A class-4 myosin would have originated in the ancestor of the SAR/Haptophyceae. The class-4 myosins present in Centramoebida species (Lobosea) and *Thecamonas* (Apusozoa) . Putative horizontal gene transfer (HGT) events are shown by dashed arrows.
B) In a gene loss scenario, class-2 and class-4 would have been present in the LECA. Accordingly, class-2 and class-4 myosins must have been lost independently in many lineages as indicated. Some loss events could be considered single events if the respective lineages have a common ancestry. Such common events are indicated by green lines. If a common ancestry of the SAR/Haptophyceae and the Archaeplastida/Cryptophyta were assumed (Diaphoretickes hypothesis), the class-2 myosin loss events in both branches could even be joined to a single event (green dotted line).

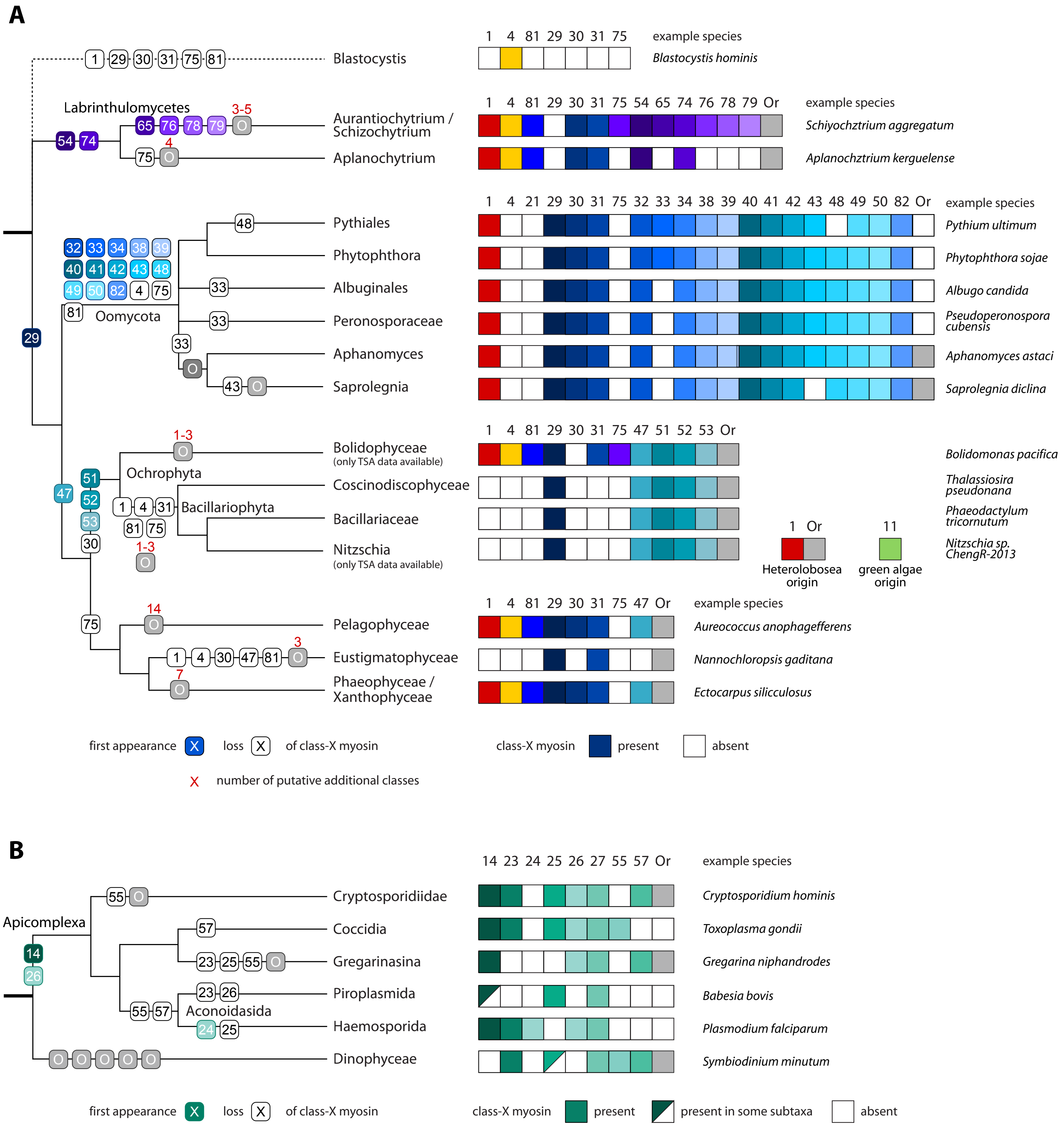

Fig. S15: Myosin gain and loss plotted onto the most widely accepted phylogenetic tree of the Alveolata and Stramenopiles.
(A) Stramenopiles myosin evolution. (B) Alveolate myosin evolution.

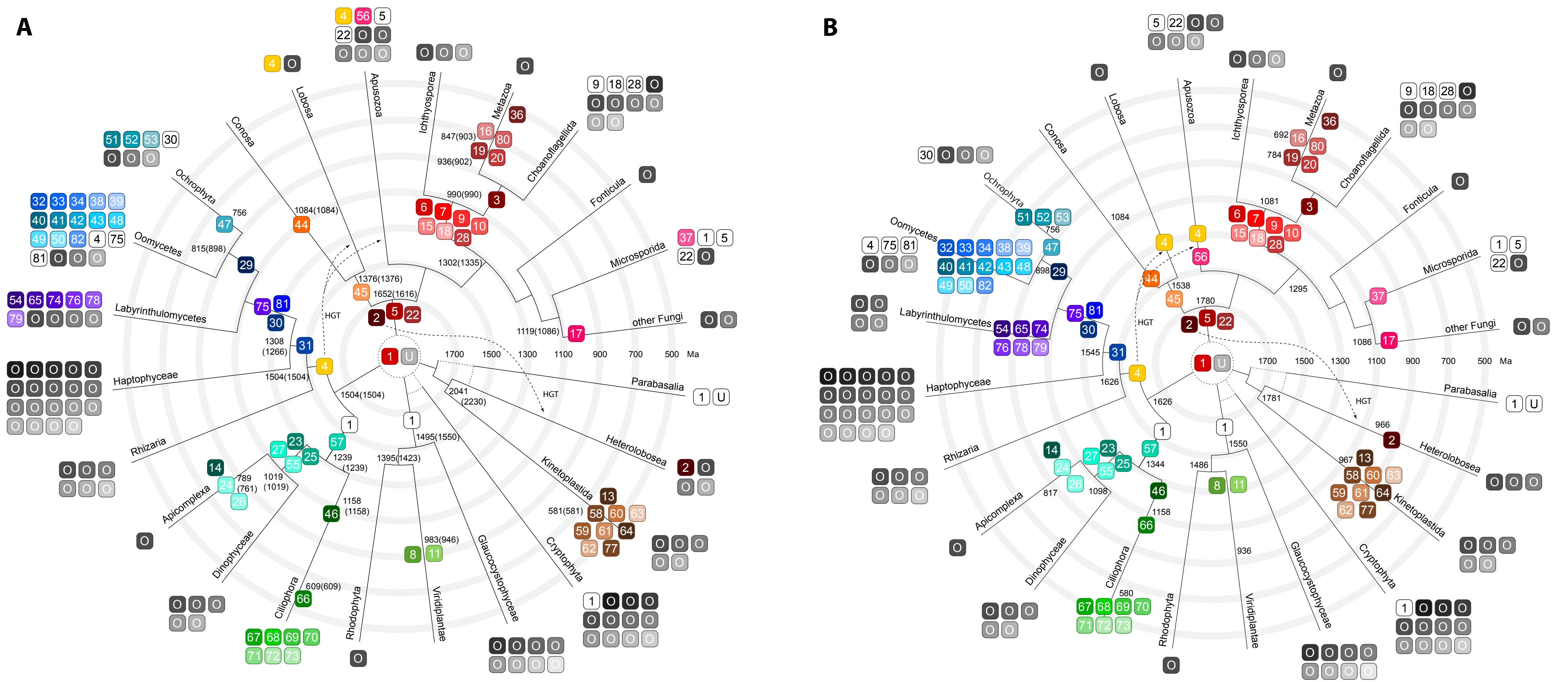

Fig. S16: Myosin evolution according to different molecular time estimates. This Figure presents the same phylogenetic tree as in Fig. 4. (A) In contrast to Fig. 4, the TimeTree Of Life divergence time estimates (Hedges et al. 2015) have been used as reference here. The TimeTree Of Life divergence time estimates are average time estimates from multiple studies. Because there are often considerable differences, we plotted both the median and the mean time estimates (the latter is given in brackets) at nodes and branches. (B) In contrast to Fig. 4, myosin innovation events have been placed at stems representing their first possible appearance. (A) and (B): Numbers at branches denote divergence times of splits that are not shown because of space limitations. Controversial branchings are indicated by dotted lines. Putative horizontal gene transfer (HGT) events are shown by dashed arrows. Myosin class inventions are represented by colored boxes, orphan myosins indicating potential further classes are shown in grey, and white boxes mark myosin loss events. Myosin classes and orphans, whose ancestry could not be assigned to nodes with known divergence times, were placed at branch ends. The supposed second myosin prototype in the LECA is indicated by an "U" in the center of the tree. The last common eukaryotic ancestor must have contained at least two myosins each containing a motor domain connected to a C-terminal IQ motif: a class-1 prototype myosin and an unknown myosin, from which all other classes evolved. The almost ubiquitous distribution of the class-1 myosins and the number of conserved intron positions shared between class-1 and all other myosins strongly suggest an ancient class-1 prototype motor. Because of the unique and invariant proline insertion at the base of the lever-arm helix of all class-1 myosins, it is very unlikely that new classes evolved from class-1 myosins several times and independently lost the proline insertion. Instead, assuming a second prototype myosin in the LECA without the class-1 specific proline insertion seems way more likely.
